# Supplementary material for: Water-promoted C-S bond formation reactions
Source: Nat Commun. 2018 Apr 3;9:1321. doi: 10.1038/s41467-018-03698-8 (PMC5883052; doi:10.1038/s41467-018-03698-8)
Supplement: Supplementary file 1 — Supplementary Information(PDF 4934 kb) [file 41467_2018_3698_MOESM1_ESM.pdf]

Water-promoted C-S bond formation reactions

Xie *et al*.

Supplementary Methods

Unless otherwise noted, all commercially available compounds were used as received. All solvents were purified according to standard procedures. The ^1H NMR and spectra was recorded at 400MHz, ^{13}C NMR was recorded at 101MHz. ^1H and ^{13}C NMR Chemical shifts were calibrated to tetramethylsilane as an external reference. Data are reported in the following order: chemical shift (δ) in ppm; multiplicities are indicated s (singlet), d (doublet), t (triplet), q (quartet), dd (doublet of doublets), m (multiplet), brs (broad singlet); coupling constants (*J*) are in Hertz (Hz). HRMS were obtained on an IonSpec FT-ICR mass spectrometer with ESI resource. Melting points were measured on a RY-I apparatus and are reported uncorrected. The starting materials sulfinic acids (*1*), allylic alcohols (2), potassium 4-(5-chloro-6'-methyl-[2,3'-bipyridin]-3-yl)benzenesulfinate (3) were readily prepared according to the related literatures. The starting materials hypotaurine and cysteine sulfinic acid were purchased from *J&K Chemical Ltd* (Shanghai).

Computational methods: All of the DFT calculations were performed with the Gaussian 09 series of programs (*4*). The geometry optimization of all the minima and transition states involved were performed at the B3LYP levels of theory with the 6-31+G(d) basis set used. The vibrational frequencies were computed at the same level of theory to check whether each optimized structure is an energy minimum or a transition state and to evaluate its zero-point vibrational energy (ZPVE). The computed structures are illustrated as was shown, S: yellow, C: gray, O: red, H: white.

Supplementary Table 1|Screening solvents I: reactions in organic solvents.

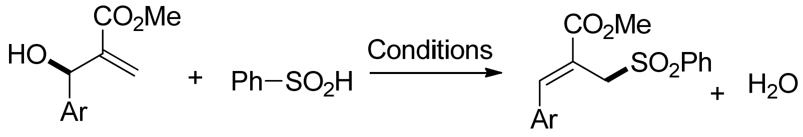

1a (Ar = 4-BrC$_6$H$_4$) **2a** **3a**

entry	solvent	Temp.(°C)	*t* (h)	yield (%)
1	Toluene	80	48	14
2	PhCl	80	48	18
3	1,4-dioxane	80	48	trace
4	THF	reflux	48	trace
5	DMF	80	48	21
6	*i*-PrOH	80	48	trace
7	EtOH	reflux	48	trace
8	CH_3CN	reflux	48	6
9	--	80	48	56
10	H_2O	80	48	21

Reaction conditions: **1a** (0.3 mmol) and **2a** (0.45 mmol) dissolved in solvent (2.0 mL) and then stirred under identity temperature. *NMR yields (using mesitylene as internal stand).

Supplementary Table 2|Screening solvents II: reactions in aqueous media.

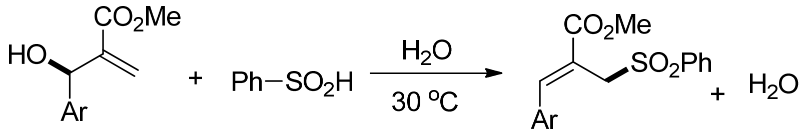

1a (Ar = 4-BrC$_6$H$_4$) **2a** **3a**

entry	solvent	Temp.(°C)	*t* (h)	yield (%)
11	H_2O/EtOH	80	3	98
12	H_2O	80	3	2
13	H_2O/CH_3CN	80	5	83
14	H_2O/EtOH	30	48	91
15	H_2O	30	48	25
16	H_2O/DMSO	30	48	87
17†	H_2O/EtOH	30	48	92
18‡	H_2O/EtOH	30	48	90
19§	PBS/EtOH	30	48	85
20*	H_2O/EtOH	30	48	72

Reaction conditions: **1a** (0.3 mmol) and **2a** (0.45 mmol) dissolved in solvent 2.0 mL (for the co-solvent, V/V = 1/1, deionized water) and then stirred under identity temperature. Isolated yield. † Aqueous media (H_2O/EtOH/ (V/V = 1/1) was prepared by using fresh distilled water and EtOH. ‡Reaction in plastic tube other than glass tube. §PBS pH 4.92. *aqueous media H_2O/EtOH/ (V/V = 3/1).

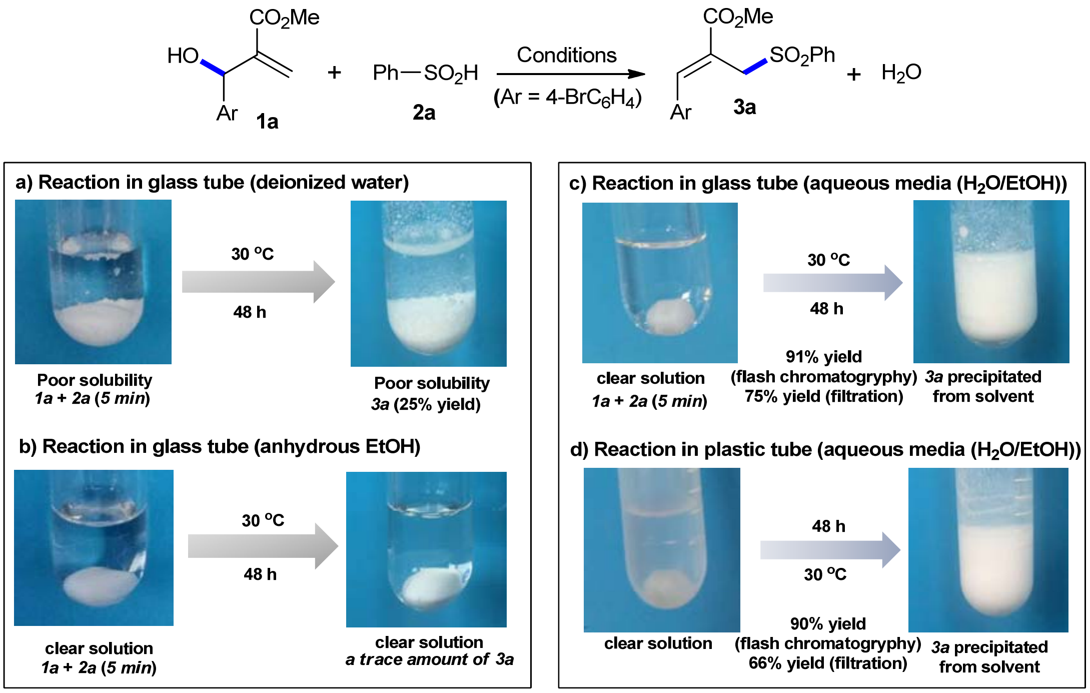

Supplementary Figure 1 | Reaction phenomenon and results in different solvents.

All the reactions were performed with **1a** (81 mg, 0.3 mmol) and **2a** (64 mg, 0.45 mmol) in solvent (2.0 mL) at 30 °C for 48h. **a** In glass tube with deionized water (2.0 mL) as solvent. **b** In glass tube with anhydrous ethanol (2.0 mL) as solvent. **c** In glass tube with aqueous media (ethanol 1.0 mL and deionized water 1.0 mL) as solvent. **d** In plastic tube with aqueous media (ethanol 1.0 mL and deionized water 1.0 mL) as solvent.

Supplementary Table 3|The influence of the amount water on reaction results.

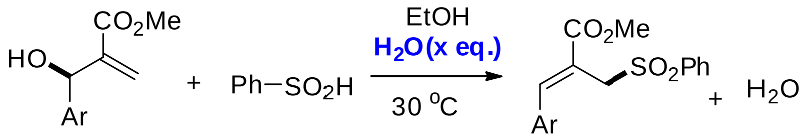

1a (Ar = 4-BrC$_6$H$_4$) **2a** **3a**

	1	2	3	4	5	6	7	8	9	10[b]	11
H_2O (eq.)	0	0.3	1.0	2.0	5.0	10	30	60	85	92	140
Yield (%)[a]	3	8	14	34	51	56	64	90	92	90	74

Reaction conditions: **1a** (0.4 mmol) and **2a** (0.6 mmol) dissolved in solvent 2.0 mL (mixture of anhydrous EtOH and deionized water) and then stirred at 30 °C for 48h. [a] Isolated yield. [b] anhydrous EtOH/deionized water = 1/1(V/V).

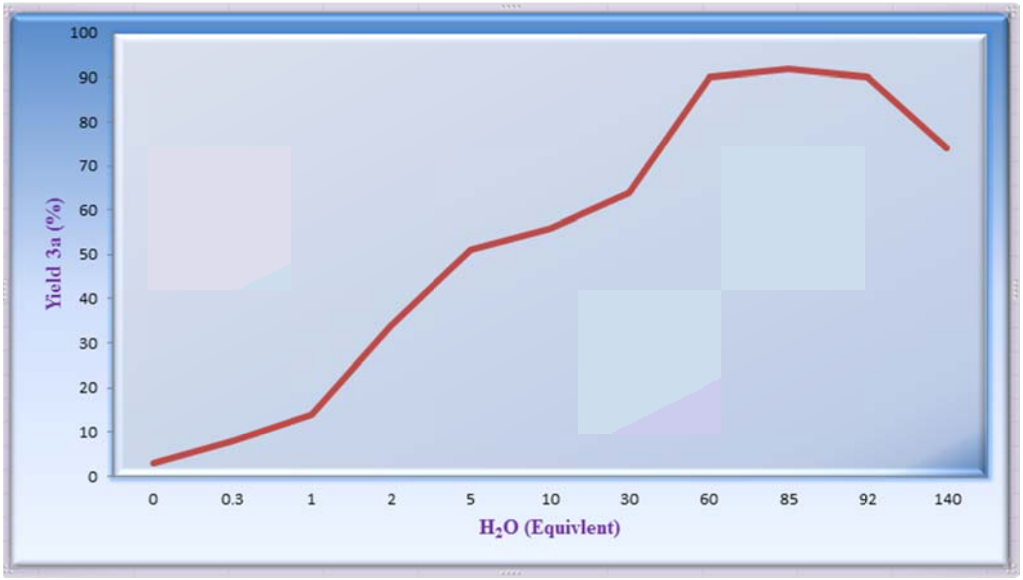

General Procedure for the allylic sulfones 3. Sulfinic acid **2** (0.45 mmol) and allylic alcohol **1** (0.3 mmol) was dissolved in aqueous media 2.0 mL (ethanol/deionized water, V/V =1/1) and stirred vigorously at 30 °C. After complete conversion, the desired allylic sulfones **3** precipitated from the solvent. Then the aqueous media was extracted with ethyl acetate (2*5 mL). The combined organic layers were dried over sodium sulfate, and concentrated by rotary evaporation to afford crude product, which was then purified *via* column chromatography (Petroleum ether (bp: 60-90 °C)/ethyl acetate = 5:1) to obtain the corresponding products **3**. Unless otherwise noted, the allylic sulfones were isolated following this method.

Practical purifications method without column chromatography. Sulfinic acid **2** (0.45 mmol) and allylic alcohol **1** (0.3 mmol) was suspended in aqueous media 2.0 mL (ethanol/deionized water, V/V =1/1) and stirred vigorously at 30 °C. After complete conversion, allylic sulfones **3** precipitated (In most cases, the products precipitated out as solid. For some case, such as **3e**, **3f**, **3h**, **3j**, **3q**, **3s** was precipitated as sticky oil, probably due to their lower melting point or lower yields.) from the solvent and then filtered and dried under vacuum to yield corresponding products 3 (As representative examples, **3a** was isolated in 75% yield, **3u** can be isolated in 84% yield by filtration). This method is powerful in gram-scale reactions and can be carried out in a higher concentration. **3f** and **3u** was generated from MBH alcohols and 1,3-diarylprop-2-en-1-ols respectively. In addition, both

of them were obtained in a lower yield than their analogues. Thus, the reactions for generating **3f** and **3u** conducted in the gram scale will be representative samples. In the gram scale reactions, **3u** can be isolated by this method conveniently. Due to the lower melting point, **3f** firstly precipitated as sticky oil (the purity of the sticky oil is over 95% by NMR), which was followed by recrystallization from 10 mL EtOH/H_2O (V/V = 1/1) and filtration to afford a white solid **3f**. (For the detail, see The procedure for gram scale reaction purified by filtration (**3f**), (**3u**)).

methyl (Z)-3-(4-bromophenyl)-2-((phenylsulfonyl)methyl)acrylate (3a)

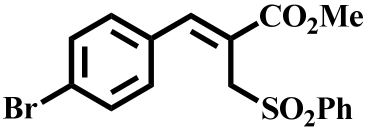

Following the general procedure, the reaction was conducted at 30 °C for 48h. **3a** was isolated as white solid (91% yield when purified via column chromatography; 66% yield purified via filtration when the reaction conducted in plastic tube; and 75% yield in glass tube). Mp: 96-98 °C. ^{1}H NMR (400 MHz, $CDCl_3$) δ = 7.89 – 7.86 (m, 2H), 7.85 – 7.83 (m, 1H), 7.64 (t, *J* = 7.5 Hz, 1H), 7.55 – 7.47 (m, 4H), 7.40 (d, *J* = 8.4 Hz, 2H), 4.44 (s, 2H), 3.58 (s, 3H). ^{13}C NMR (101 MHz, $CDCl_3$) δ = 166.62, 145.13, 139.24, 133.93, 132.51, 132.07, 130.77, 129.14, 128.54, 124.32, 121.49, 55.09, 52.53. HRMS (ESI/$[M+H]^+$) Calcd. for: $C_{17}H_{16}BrO_4S$ 394.9953, found 394.9949.

methyl (Z)-3-(4-bromophenyl)-2-(tosylmethyl)acrylate (3b)

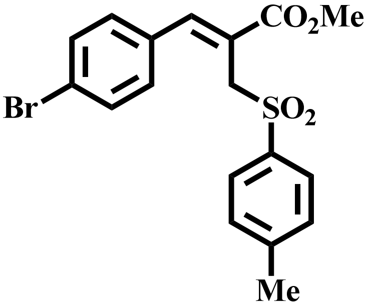

Following the general procedure, the reaction was conducted at 30 °C for 72h. **3b** was isolated as white solid. Mp: 112-114 °C. ^{1}H NMR (400 MHz, $CDCl_3$) δ = 7.85 (s, 1H), 7.71 (d, *J* = 8.2, 2H), 7.50 (d, *J* = 8.5, 2H), 7.37 (d, *J* = 8.4, 2H), 7.29 (d, *J* = 8.1, 2H), 4.43 (s, 2H), 3.61 (s, 3H), 2.44 (s, 3H). ^{13}C NMR (101 MHz, $CDCl_3$) δ 166.70, 144.98, 144.82, 136.25, 132.55, 131.97, 130.73, 129.71, 128.54, 124.19, 121.69, 55.03, 52.51, 21.68. HRMS (ESI/$[M+H]^+$) Calcd. for: $C_{18}H_{18}BrO_4S$ 409.0109, found 409.0111.

methyl (Z)-3-(4-bromophenyl)-2-(((4-chlorophenyl)sulfonyl)methyl)acrylate (3c)

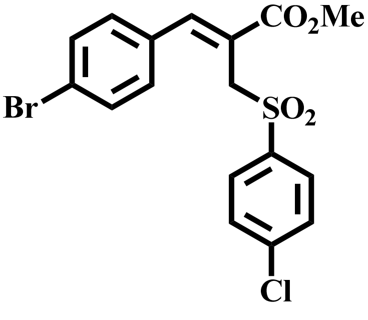

Following the general procedure, the reaction was conducted at 30 °C for 48h. **3c** was isolated as white

solid. Mp: 153-154 °C . ^{1}H NMR (400 MHz, $CDCl_3$) δ = 7.87 (s, 1H), 7.75 (d, *J* = 8.5, 2H), 7.53 (d, *J* = 8.4, 2H), 7.46 (d, *J* = 8.5, 2H), 7.35 (d, *J* = 8.5, 2H), 4.45 (s, 2H), 3.65 (s, 3H). ^{13}C NMR (101 MHz, $CDCl_3$) δ = 166.58, 145.27, 140.85, 137.54, 132.44, 132.15, 130.68, 130.08, 129.44, 124.44, 121.34, 54.94, 52.66. HRMS (ESI/[M+H]$^+$) Calcd. for: $C_{17}H_{15}BrClO_4S$ 428.9563, found 428.9567.

methyl (Z)-3-(4-bromophenyl)-2-((methylsulfonyl)methyl)acrylate (3d)

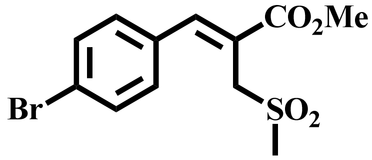

Following the general procedure, the reaction was conducted at 30 °C for 48h. **3d** was isolated as white solid. Mp: 103-105 °C. ^{1}H NMR (400 MHz, $CDCl_3$) δ = 8.06 (s, 1H), 7.59 (d, *J* = 8.5, 2H), 7.49 (d, *J* = 8.4, 2H), 4.30 (s, 2H), 3.89 (s, 3H), 3.02 (s, 3H). ^{13}C NMR (101 MHz, $CDCl_3$) δ 167.04, 146.02, 132.44, 132.26, 130.98, 124.72, 121.24, 54.31, 52.88, 42.71. HRMS (ESI/[M+H]$^+$) Calcd. for: $C_{12}H_{14}BrO_4S$ 332.9796, found 332.9789.

methyl (Z)-3-(4-bromophenyl)-2-((ethylsulfonyl)methyl)acrylate (3e)

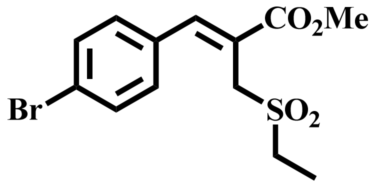

Following the general procedure, the reaction was conducted at 30 °C for 48h. **3e** was isolated as white solid. Mp: 78-79 °C. ^{1}H NMR (400 MHz, $CDCl_3$) δ = 8.04 (s, 1H), 7.59 (d, *J* = 8.4, 2H), 7.50 (d, *J* = 8.5, 2H), 4.25 (s, 2H), 3.88 (s, 3H), 3.12 (q, *J* = 7.4, 2H), 1.42 (t, *J* = 7.4, 3H). ^{13}C NMR (101 MHz, $CDCl_3$) δ 167.11, 145.77, 132.56, 132.20, 130.96, 124.60, 121.28, 52.82, 51.67, 49.09, 6.55. HRMS (ESI/[M+H]$^+$) Calcd. for: $C_{13}H_{16}BrO_4S$ 346.9953, found 346.9955.

methyl (Z)-3-phenyl-2-((phenylsulfonyl)methyl)acrylate (3f)

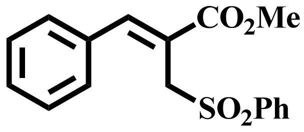

Following the general procedure, the reaction was conducted at 30 °C for 48h. **3f** was isolated as white solid. Mp: 64-66 °C. ^{1}H NMR (400 MHz, $CDCl_3$) δ = 7.95 (s, 1H), 7.94 – 7.82 (m, 2H), 7.61 (d, *J* = 7.5, 1H), 7.54 – 7.46 (m, 4H), 7.45 – 7.34 (m, 3H), 4.50 (s, 2H), 3.59 (s, 3H). ^{13}C NMR (101 MHz, $CDCl_3$) δ = 166.91, 146.47, 139.33, 133.79, 133.65, 129.76, 129.22, 129.06, 128.83, 128.57, 120.88, 55.15, 52.43. HRMS (ESI/[M+H]$^+$) Calcd. for: $C_{17}H_{17}O_4S$ 317.0848, found 317.0845.

The procedure for gram scale reaction purified by filtration (3f): benzenesulfinic acid (2.84g, 20 mmol) and methyl 2-(hydroxy(phenyl)methyl)acrylate (2.5g, 13 mmol) was suspended in aqueous media 50 mL (ethanol/deionized water, V/V =1/1) and stirred vigorously at 30 °C for 48h. Allylic sulfones **3f** precipitated as sticky oil (the purity of the sticky oil is over 95% by NMR), which was followed by recrystallization from 10 mL EtOH/H_2O (V/V = 1/1) afforded a white solid **3f** (3.01g).

ethyl (Z)-2-((phenylsulfonyl)methyl)-3-(p-tolyl)acrylate (3g)

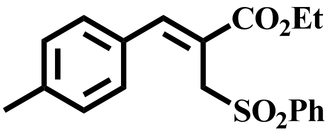

Following the general procedure, the reaction was conducted at 30 °C for 48h. **3g** was isolated as white solid. Mp: 74-76 °C. ^{1}H NMR (400 MHz, $CDCl_3$) δ = 7.92 (s, 1H), 7.88 (d, *J* = 7.8, 2H), 7.61 (t, *J* = 7.4, 1H), 7.50 (t, *J* = 7.6, 2H), 7.44 (d, *J* = 7.9, 2H), 7.20 (d, *J* = 7.9, 2H), 4.51 (s, 2H), 4.02 (q, *J* = 7.1, 2H), 2.38 (s, 3H), 1.22 (t, *J* = 7.1, 3H). ^{13}C NMR (101 MHz, $CDCl_3$) δ 166.64, 146.31, 140.21, 139.50, 133.73, 130.92, 129.54, 129.45, 129.00, 128.60, 120.04, 61.48, 55.27, 21.44, 14.11. HRMS (ESI/[M+H]$^+$) Calcd. for: $C_{19}H_{21}O_4S$ 345.1161, found 345.1154.

ethyl (Z)-3-(4-fluorophenyl)-2-((phenylsulfonyl)methyl)acrylate (3h)

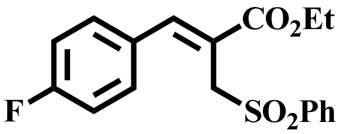

Following the general procedure, the reaction was conducted at 30 °C for 48h. **3h** was isolated as white solid. Mp: 50-52 °C. ^{1}H NMR (400 MHz, $CDCl_3$) δ = 7.91 (s, 1H), 7.90 – 7.85 (m, 2H), 7.63 (t, *J* = 7.5, 1H), 7.59 – 7.48 (m, 4H), 7.08 (t, *J* = 8.6, 2H), 4.47 (s, 2H), 4.02 (q, *J* = 7.1, 2H), 1.22 (t, *J* = 7.1, 3H). ^{13}C NMR (101 MHz, $CDCl_3$) δ = 166.33, 163.44 (d, $J_{C\text{-}F}$ = 251.4 Hz), 144.97, 139.37, 133.88, 131.46 (d, $J_{C\text{-}F}$ = 8.5 Hz), 129.87 (d, $J_{C\text{-}F}$ = 3.3 Hz), 129.09, 128.56, 120.92, 115.97 (d, $J_{C\text{-}F}$ = 21.7 Hz), 61.63, 55.10, 14.08. HRMS (ESI/[M+H]$^+$) Calcd. for: $C_{18}H_{18}FO_4S$ 349.0910, found 349.0898.

Ethyl (Z)-3-(4-nitrophenyl)-2-((phenylsulfonyl)methyl)acrylate (3i)

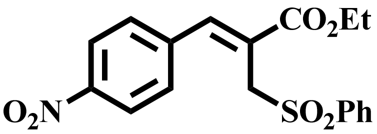

Following the general procedure, the reaction was conducted at 30 °C for 48h. **3i** was isolated as white solid. Mp:115-116 °C. ^{1}H NMR (400 MHz, $CDCl_3$) δ = 8.25 (d, *J* = 8.5, 2H), 7.98 (s, 1H), 7.87 (d, *J* = 7.8, 2H), 7.72 – 7.63 (m, 3H), 7.54 (t, *J* = 7.6, 2H), 4.42 (s, 2H), 4.09 (q, *J* = 7.1, 2H), 1.26 (t, *J* = 7.1, 3H). ^{13}C NMR (101 MHz, $CDCl_3$) δ =165.63, 148.06, 143.26, 140.12, 139.15, 134.10, 129.92, 129.26, 128.52, 124.52, 123.94, 62.07, 54.91, 14.06. HRMS (ESI/[M+H]$^+$) Calcd. for: $C_{18}H_{18}NO_6S$ 376.0855, found 376.0858.

ethyl (Z)-2-((phenylsulfonyl)methyl)-3-(4-(trifluoromethyl)phenyl)acrylate (3j)

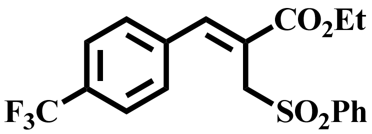

Following the general procedure, the reaction was conducted at 30 °C for 48h. **3j** was isolated as white solid. Mp: 66-67 °C. ^{1}H NMR (400 MHz, $CDCl_3$) δ = 7.96 (s, 1H), 7.87 – 7.81 (m, 2H), 7.64 (dd, *J* = 7.8, 4.2, 3H), 7.58 (d, *J* = 8.3, 2H), 7.50 (t, *J* = 7.8, 2H), 4.44 (s, 2H), 3.63 (s, 3H). ^{13}C NMR (101 MHz,

$CDCl_3$) δ = 166.36, 144.48, 139.10, 137.17, 133.99, 131.20 (q, J_{C-F} = 32.8 Hz), 129.27, 129.18, 128.82 (d, J_{C-F} = 14.8 Hz), 128.48, 125.70 (q, J_{C-F} = 3.7 Hz), 123.78 (q, J_{C-F} = 273.7 Hz), 122.43, 54.84, 52.64.HRMS (ESI/$[M+H]^+$) Calcd. for: $C_{19}H_{18}F_3O_4S$ 399.0878, found 399.0874.

ethyl (Z)-3-(4-cyanophenyl)-2-((phenylsulfonyl)methyl)acrylate (3k)

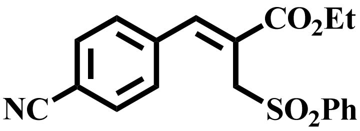

Following the general procedure, the reaction was conducted at 30 °C for 48h. **3k** was isolated as white solid. Mp: 106-108 °C. ^1H NMR (400 MHz, $CDCl_3$) δ = 7.93 (s, 1H), 7.86 (d, J = 7.4, 2H), 7.72 – 7.60 (m, 5H), 7.53 (t, J = 7.8, 2H), 4.41 (s, 2H), 4.07 (q, J = 7.1, 2H), 1.25 (t, J = 7.1, 3H). ^{13}C NMR (101 MHz, $CDCl_3$) δ 165.72, 143.68, 139.16, 138.23, 134.07, 132.48, 129.63, 129.23, 128.52, 124.05, 118.28, 113.05, 62.01, 54.92, 14.06. HRMS (ESI/$[M+H]^+$) Calcd. for: $C_{19}H_{18}NO_4S$ 356.0957, found 356.0950.

ethyl (Z)-3-(4-formylphenyl)-2-((phenylsulfonyl)methyl)acrylate (3l)

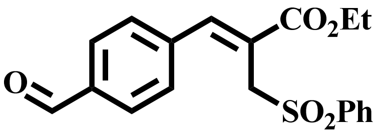

Following the general procedure, the reaction was conducted at 30 °C for 48h. **3l** was isolated as white solid. Mp: 90-92 °C. ^1H NMR (400 MHz, $CDCl_3$) δ = 10.03 (s, 1H), 7.99 (s, 1H), 7.94 (s, 1H), 7.92 – 7.84 (m, 3H), 7.81 (d, J = 7.6, 1H), 7.65 – 7.56 (m, 2H), 7.50 (t, J = 7.6, 2H), 4.47 (s, 2H), 4.10 (q, J = 7.1, 2H), 1.27 (t, J = 7.1, 3H). ^{13}C NMR (101 MHz, $CDCl_3$) δ = 191.61, 166.00, 144.32, 139.17, 136.73, 134.77, 134.60, 133.94, 130.48, 130.18, 129.64, 129.16, 128.56, 122.97, 61.88, 54.88, 14.10.HRMS (ESI/$[M+H]^+$) Calcd. for: $C_{19}H_{19}O_5S$ 359.0953, found 359.0946.

ethyl (Z)-3-(3-bromophenyl)-2-((phenylsulfonyl)methyl)acrylate (3m)

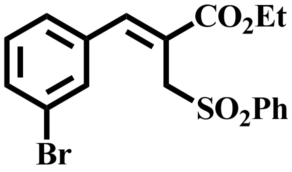

Following the general procedure, the reaction was conducted at 30 °C for 48h. **3m** was isolated as white solid. Mp:107-108 °C. ^1H NMR (400 MHz, $CDCl_3$) δ = 7.85 – 7.80 (m, 3H), 7.67-7.61 (m, 1H), 7.52 – 7.46 (m, 3H), 7.45 – 7.38 (m, 2H), 7.28 – 7.21 (m, 1H), 4.45 (s, 2H), 4.12 (q, J = 7.1, 2H), 1.27 (t, J = 7.1, 3H). ^{13}C NMR (101 MHz, $CDCl_3$) δ 166.05, 144.05, 138.98, 135.74, 133.92, 132.40, 131.74, 130.28, 129.12, 128.52, 127.29, 122.85, 61.82, 54.78, 14.11. HRMS (ESI/$[M+H]^+$) Calcd. for: $C_{18}H_{18}BrO_4S$ 409.0109, found 409.0097.

ethyl (Z)-3-(2-chlorophenyl)-2-((phenylsulfonyl)methyl)acrylate (3n)

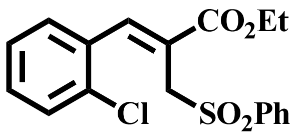

Following the general procedure, the reaction was conducted at 30 °C for 48h. **3n** was isolated as oil. ^{1}H NMR (400 MHz, $CDCl_3$) δ = 8.01 (s, 1H), 7.83 (dd, *J* = 8.3, 1.1 Hz, 2H), 7.65 – 7.57 (m, 2H), 7.49 (t, *J* = 7.7 Hz, 2H), 7.39 – 7.34 (m, 1H), 7.33 – 7.26 (m, 2H), 4.40 (s, 2H), 4.09 (q, *J* = 7.1, 2H), 1.25 (t, *J* = 7.1, 3H). ^{13}C NMR (101 MHz, $CDCl_3$) δ 165.91, 142.89, 139.30, 134.04, 133.80, 132.33, 130.59, 129.99, 129.75, 129.12, 128.44, 127.05, 123.34, 61.76, 54.89, 14.09. HRMS (ESI/$[M+H]^+$) Calcd. for: $C_{18}H_{18}ClO_4S$ 365.0614, found 365.0609.

ethyl (Z)-3-(2,4-dichlorophenyl)-2-((phenylsulfonyl)methyl)acrylate (3o)

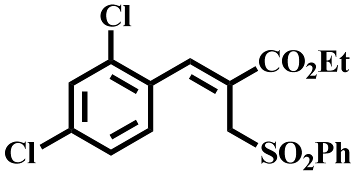

Following the general procedure, the reaction was conducted at 30 °C for 48h. **3o** was isolated as white solid. Mp: 92-94 °C.^{1}H NMR (400 MHz, $CDCl_3$) δ = 7.95 (s, 1H), 7.89 – 7.80 (m, 2H), 7.70 – 7.60 (m, 2H), 7.56-7.49 (m, 2H), 7.40 (d, *J* = 1.9, 1H), 7.31 (dd, *J* = 8.3, 1.8, 1H), 4.35 (s, 2H), 4.07 (q, *J* = 7.1, 2H), 1.25 (t, *J* = 7.1, 3H). ^{13}C NMR (101 MHz, $CDCl_3$) δ 165.68, 141.78, 139.28, 136.02, 134.87, 133.91, 130.89, 129.70, 129.18, 128.46, 127.48, 123.83, 61.88, 54.94, 14.06. HRMS (ESI/[M+H]+) Calcd. for: $C_{18}H_{17}Cl_2O_4S$ 399.0225, found 399.0237.

ethyl (E)-3-(naphthalen-1-yl)-2-((phenylsulfonyl)methyl)acrylate (3p)

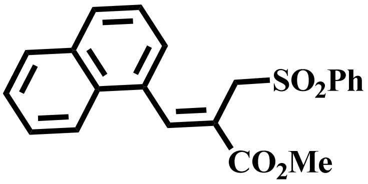

Following the general procedure, the reaction was conducted at 30 °C for 48h. **3p** was isolated as white solid. Mp:92-93 °C. ^{1}H NMR (400 MHz, $CDCl_3$) δ = 8.44 (s, 1H), 7.83 (d, *J* = 8.0, 2H), 7.69 (t, *J* = 7.5, 3H), 7.57 – 7.41 (m, 4H), 7.36 (t, *J* = 7.4, 1H), 7.31-7.24 (m, 2H), 4.46 (s, 2H), 3.74 (s, 3H). ^{13}C NMR (101 MHz, $CDCl_3$) δ 166.65, 144.59, 133.31, 131.01, 130.63, 129.82, 128.86, 128.59, 128.13, 126.71, 126.45, 126.36, 125.31, 124.27, 123.41, 55.02, 52.62. HRMS (ESI/[M+H]+) Calcd. for: $C_{21}H_{19}O_4S$ 367.1004, found 367.1021.

ethyl (E)-2-((phenylsulfonyl)methyl)-3-(thiophen-2-yl)acrylate (3q)

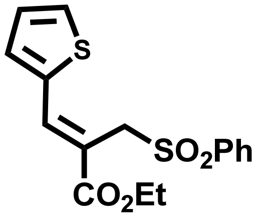

Following the general procedure, the reaction was conducted at 30 °C for 48h. **3q** was isolated as white solid. Mp: 84-86 °C. ^{1}H NMR (400 MHz, CDCl$_3$) δ = 8.06 (s, 1H), 7.92 (d, *J* = 7.6, 2H), 7.60 (d, *J* = 6.9, 1H), 7.52 (d, *J* = 7.5, 4H), 7.15 – 7.05 (m, 1H), 4.65 (s, 2H), 3.98 (q, *J* = 7.1, 2H), 1.18 (t, *J* = 7.1, 3H). ^{13}C NMR (101 MHz, CDCl$_3$) δ 166.33, 139.43, 138.03, 136.84, 134.14, 133.80, 130.79, 128.98, 128.69, 127.80, 116.47, 61.50, 55.95, 14.11. HRMS (ESI/[M+H]+) Calcd. for: $C_{16}H_{17}O_4S_2$ 337.0568, found 337.0565.

3-(4-chlorophenyl)-2-((phenylsulfonyl)methyl)acrylonitrile (3r)

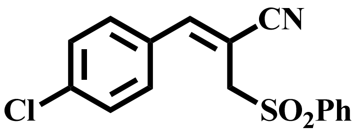

Following the general procedure, the reaction was conducted at 30 °C for 48h. **3r** was isolated as white solid. Mp:141-142 °C. ^{1}H NMR (400 MHz, CDCl$_3$) δ = 7.96 – 7.88 (m, 2H), 7.79 – 7.69 (m, 1H), 7.68 – 7.58 (m, 4H), 7.41 (d, *J* = 8.6, 2H), 7.06 (s, 1H), 4.04 (s, 2H). ^{13}C NMR (101 MHz, CDCl$_3$) δ 150.41, 137.78, 137.47, 134.77, 130.83, 130.53, 129.67, 129.43, 128.79, 116.86, 98.56, 61.25. HRMS (ESI/[M+H]+) Calcd. for: $C_{16}H_{13}ClNO_2S$ 318.0356, found 318.0353.

methyl (Z)-2-((phenylsulfonyl)methyl)hex-2-enoate (3s)

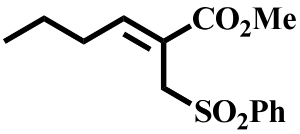

Following the general procedure, the reaction was conducted at 30 °C for 48h. **3s** was isolated as white solid. Mp: 37-39°C. ^{1}H NMR (400 MHz, CDCl$_3$) δ = 7.89 – 7.83 (m, 2H), 7.68 – 7.60 (m, 1H), 7.54 (t, *J* = 7.7, 2H), 7.13 (t, *J* = 7.6, 1H), 4.25 (s, 2H), 3.49 (s, 3H), 2.19 (q, *J* = 7.5, 2H), 1.46 (dd, *J* = 14.8, 7.4, 2H), 0.92 (t, *J* = 9.0, 5.8, 3H). ^{13}C NMR (101 MHz, CDCl$_3$) δ 166.07, 151.80, 138.85, 133.75, 129.02, 128.74, 120.59, 54.09, 52.07, 31.48, 21.58, 13.86. HRMS (ESI/[M+H]+) Calcd. for: $C_{14}H_{19}O_4S$ 283.1004, found 283.0996.

(Z)-2-(4-chlorobenzylidene)-3-(phenylsulfonyl)cyclohexan-1-one (3t)

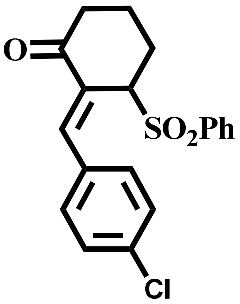

Following the general procedure, the reaction was conducted at 30 °C for 24h. **3t** was isolated as white solid. Mp: 139-140 °C. ^{1}H NMR (400 MHz, CDCl$_3$) δ = 7.90 (t, *J* = 4.2, 1H), 7.66 (d, *J* = 7.9, 2H), 7.58 (t, *J* = 7.3, 1H), 7.44 (t, *J* = 7.7, 2H), 7.32 – 7.20 (m, 4H), 5.74 (s, 1H), 2.68 – 2.46 (m, 2H), 2.38 (dd, *J* = 8.2, 5.4, 2H), 1.97 (dq, *J* = 12.2, 5.9, 2H). ^{13}C NMR (101 MHz, CDCl$_3$) δ 195.90, 150.75, 138.05, 134.91, 133.81, 132.68, 131.31, 130.87, 128.91, 128.85, 128.80, 64.31, 37.65, 26.52, 22.18. HRMS

(ESI/[M+H]+) Calcd. for: $C_{19}H_{18}ClO_3S$ 361.0665, found 361.0661.

(E)-(3-(phenylsulfonyl)prop-1-ene-1,3-diyl)dibenzene (3u)

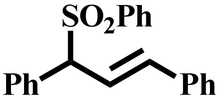

Following the general procedure, the reaction was conducted at 30 °C for 3h. **3u** was isolated as white solid (92% yield *via* column chromatography; 84% yield via filtration). Mp: 149-150 °C. ^{1}H NMR (400 MHz, $CDCl_3$) δ = 7.67 (d, J = 7.9, 2H), 7.56 (t, J = 7.4, 1H), 7.41 (t, J = 7.5, 2H), 7.38 – 7.24 (m, 10H), 6.63 – 6.45 (m, 2H), 4.83 (d, J = 8.5, 1H). ^{13}C NMR (101 MHz, $CDCl_3$) δ 138.21, 137.35, 135.86, 133.65, 132.26, 129.72, 129.32, 128.97, 128.74, 128.70, 128.66, 128.53, 126.79, 119.96, 75.41. HRMS (ESI/[M+H]+) Calcd. for: $C_{21}H_{19}O_2S$ 335.1100, found 335.1091.

The Procedure for gram scale reaction (3u): Benzenesulfinic acid (2.13g, 15 mmol) and (E)-1,3-diphenylprop-2-en-1-ol (2.1g, 10 mmol) was suspended in aqueous media 40 mL (ethanol/deionized water, V/V =1/1) and stirred vigorously at 30 °C for 3h. Corresponding allylic sulfone precipitated from the solvent and then filtered and dried under vacuum to yield pure **3u** as white solid (3.10g, 93% yield. The purity of precipitates **3u** was same as isolated by column chromatography).

(E)-(3-tosylprop-1-ene-1,3-diyl)dibenzene (3v)

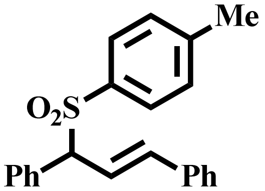

Following the general procedure, the reaction was conducted at 30 °C for 6h. **3v** was isolated as white solid. Mp: 162-164 °C. ^{1}H NMR (400 MHz, $CDCl_3$) δ = 7.58 – 7.49 (m, 2H), 7.40 – 7.24 (m, 10H), 7.20 (d, J = 8.0, 2H), 6.70 – 6.42 (m, 2H), 4.82 (d, J = 7.9, 1H), 2.39 (s, 3H). ^{13}C NMR (101 MHz, $CDCl_3$) δ 144.63, 138.00, 135.96, 134.43, 132.50, 129.73, 129.34, 128.89, 128.71, 128.65, 128.48, 126.79, 120.22, 75.37, 21.68. HRMS (ESI/[M+H]+) Calcd. for: $C_{22}H_{21}O_2S$ 349.1262, found 349.1278.

(E)-(3-((4-chlorophenyl)sulfonyl)prop-1-ene-1,3-diyl)dibenzene (3w)

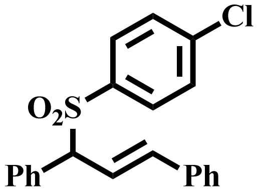

Following the general procedure, the reaction was conducted at 30 °C for 6h. **3w** was isolated as white solid. Mp:138-139 °C. ^{1}H NMR (400 MHz, $CDCl_3$) δ = 7.59-7.55 (m, 2H), 7.40 – 7.31 (m, 11H), 7.31 – 7.27 (m, 1H), 6.61 – 6.54 (m, 2H), 4.83 (dd, J = 4.9, 3.3, 1H). ^{13}C NMR (101 MHz, $CDCl_3$) δ = 140.45, 138.56, 135.91, 135.71, 132.04, 130.76, 129.70, 129.16, 129.02, 128.87, 128.73, 128.69, 126.83, 119.48, 75.52. HRMS (ESI/[M+H]+) Calcd. for: $C_{21}H_{18}ClO_2S$ 369.0716, found 369.0723.

(E)-(3-(methylsulfonyl)prop-1-ene-1,3-diyl)dibenzene (3x)

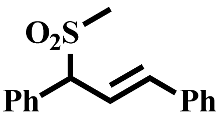

Following the general procedure, the reaction was conducted at 30 °C for 20h. **3x** was isolated as white solid. Mp: 106-107 °C. ^{1}H NMR (400 MHz, $CDCl_3$) δ = 7.54 (d, *J* = 6.6, 2H), 7.48-7.40 (m, 5H), 7.38 – 7.27 (m, 3H), 6.79 (d, *J* = 15.8, 1H), 6.65 (dd, *J* = 15.7, 9.1, 1H), 4.86 (d, *J* = 9.0, 1H), 2.81 (s, 3H). ^{13}C NMR (101 MHz, $CDCl_3$) δ 138.13, 135.56, 132.20, 129.46, 129.31, 129.21, 128.74, 126.91, 120.13, 73.84, 38.69. HRMS (ESI/[M+H]+) Calcd. for: $C_{16}H_{17}O_2S$ 273.0949, found 273.0952.

(E)-(3-(ethylsulfonyl)prop-1-ene-1,3-diyl)dibenzene (3y)

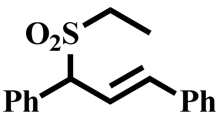

Following the general procedure, the reaction was conducted at 30 °C for 20h. **3y** was isolated as white solid. Mp: 104-105 °C. ^{1}H NMR (400 MHz, $CDCl_3$) δ = 7.54 (d, *J* = 6.4, 2H), 7.48 – 7.37 (m, 5H), 7.36-7.27 (m, 3H), 6.76 (d, *J* = 15.8, 1H), 6.64 (dd, *J* = 15.8, 9.1, 1H), 4.87 (d, *J* = 9.0, 1H), 2.93 (q, *J* = 7.5, 2H), 1.36 (t, *J* = 7.5, 3H). ^{13}C NMR (101 MHz, $CDCl_3$) δ = 137.73, 135.64, 132.21, 129.48, 129.17, 128.73, 128.70, 126.89, 120.31, 71.58, 45.19, 6.48. HRMS (ESI/[M+H]+) Calcd. for: $C_{17}H_{19}O_2S$ 287.1106, found 287.1107.

(E)-4,4'-(3-(phenylsulfonyl)prop-1-ene-1,3-diyl)bis(methylbenzene) (3z)

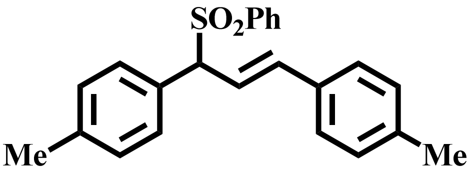

Following the general procedure, the reaction was conducted at 30 °C for 12h. **3z** was isolated as white solid. Mp: 68-70 °C. ^{1}H NMR (400 MHz, $CDCl_3$) δ = 7.68 (d, *J* = 7.5, 2H), 7.55 (t, *J* = 7.4, 1H), 7.40 (t, *J* = 7.7, 2H), 7.25-7.19 (m, 4H), 7.17 – 7.05 (m, 4H), 6.55 – 6.38 (m, 2H), 4.79 (d, *J* = 8.7, 1H), 2.32 (d, *J* = 3.0, 6H). ^{13}C NMR (101 MHz, $CDCl_3$) δ = 138.92, 138.46, 137.89, 137.46, 133.58, 133.18, 129.61, 129.46, 129.36, 129.16, 128.67, 126.69, 119.10, 75.19, 21.31, 21.26. HRMS (ESI/$[M+H]^+$) Calcd. for: $C_{23}H_{23}O_2S$ 363.1419, found 363.1411.

(E)-4,4'-(3-(phenylsulfonyl)prop-1-ene-1,3-diyl)bis(fluorobenzene) (3aa)

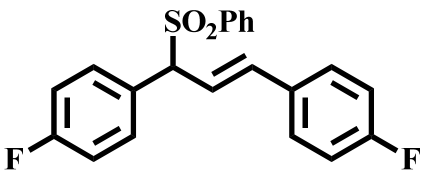

Following the general procedure, the reaction was conducted at 30 °C for 12h. **3aa** was isolated as white solid. Mp:125-126 °C. ^{1}H NMR (400 MHz, $CDCl_3$) δ = 7.66 (d, *J* = 7.5 Hz, 2H), 7.58 (t, *J* = 7.4 Hz, 1H), 7.43 (t, *J* = 7.7 Hz, 2H), 7.35 – 7.27 (m, 4H), 7.06-6.97 (m, 4H), 6.46 (d, *J* = 7.2 Hz, 2H), 4.81 (d, *J* = 7.0, 1H). ^{13}C NMR (101 MHz, $CDCl_3$) δ = 164.21 (d, J_{C-F} = 17.3 Hz), 161.74 (d, J_{C-F} = 17.0 Hz), 137.20, 137.11, 133.83, 131.90 (d, J_{C-F} = 3.3 Hz), 131.42 (d, J_{C-F} = 8.4 Hz), 129.23, 128.82,

128.44 (d, J_{C-F} = 8.2 Hz), 128.06 (d, J_{C-F} = 3.3 Hz), 119.34, 115.88 (d, J_{C-F} = 11.6 Hz), 115.66 (d, J_{C-F} = 11.7 Hz), 74.42. HRMS (ESI/$[M+H]^+$) Calcd. for: $C_{21}H_{17}F_2O_2S$ 371.0917, found 371.0925.

(E)-2,2'-(3-(phenylsulfonyl)prop-1-ene-1,3-diyl)bis(fluorobenzene) (3ab)

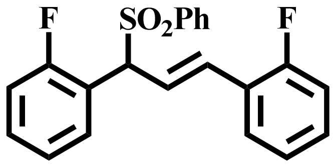

Following the general procedure, the reaction was conducted at 30 °C for 6h. **3ab** was isolated as white solid. Mp: 134-135 °C. ^1H NMR (400 MHz, $CDCl_3$) δ = 7.77 – 7.64 (m, 3H), 7.59 (t, *J* = 7.5, 1H), 7.52 – 7.38 (m, 3H), 7.37 – 7.19 (m, 3H), 7.12 (t, *J* = 7.3, 1H), 7.07 – 6.91 (m, 2H), 6.78 – 6.60 (m, 2H), 5.33 (d, *J* = 7.9, 1H). ^{13}C NMR (101 MHz, $CDCl_3$) δ = 161.66 (d, J_{C-F} = 18.6 Hz), 159.18 (d, J_{C-F} = 21.2 Hz), 137.25, 133.84, 131.70, 130.10, 129.20, 128.73, 127.90, 124.60, 124.23, 123.60, 121.50, 119.70, 115.96, 115.72, 115.46, 67.0. HRMS (ESI/$[M+H]^+$) Calcd. for:$C_{21}H_{17}F_2O_2S$ 371.0917 found 371.0935.

(E)-3,3'-(3-(phenylsulfonyl)prop-1-ene-1,3-diyl)bis(methylbenzene) (3ac)

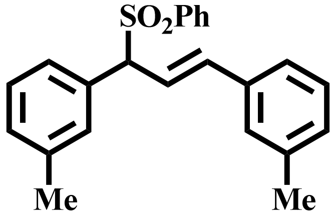

Following the general procedure, the reaction was conducted at 30 °C for 3h. **3ac** was isolated as white solid. Mp:110-111 °C. ^1H NMR (400 MHz, $CDCl_3$) δ = 7.71-7.67 (m, 2H), 7.60 – 7.54 (m, 1H), 7.43 (t, *J* = 7.8, 2H), 7.25 – 7.05 (m, 8H), 6.56 (dd, *J* = 15.7, 9.1, 1H), 6.43 (d, *J* = 15.7, 1H), 4.78 (d, *J* = 9.1, 1H), 2.33 (d, *J* = 7.8, 6H). ^{13}C NMR (101 MHz, $CDCl_3$) δ = 138.46, 138.24, 138.14, 137.46, 135.86, 133.60, 132.12, 130.39, 129.72, 129.36, 129.30, 128.65, 128.60, 128.53, 127.38, 126.82, 124.04, 119.96, 75.47, 21.41, 21.39. HRMS (ESI/$[M+H]^+$) Calcd. for: $C_{23}H_{23}O_2S$ 363.1419, found 363.1408

(E)-(2-methyl-3-(phenylsulfonyl)prop-1-ene-1,3-diyl)dibenzene (3ad)

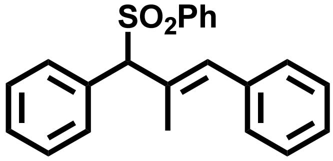

Following the general procedure, the reaction was conducted at 30 °C for 48h. **3ad** was isolated as white solid. Mp:88-89 °C. ^1H NMR (400 MHz, $CDCl_3$) δ = 7.86 – 7.76 (m, 2H), 7.63 – 7.52 (m, 3H), 7.47 (t, *J* = 7.7, 2H), 7.38-7.34 (m, 3H), 7.30 (t, *J* = 7.4, 2H), 7.22 (t, *J* = 7.3, 1H), 7.08 (d, *J* = 7.3, 2H), 6.60 (s, 1H), 4.83 (s, 1H), 1.98 (d, *J* = 1.2, 3H).^{13}C NMR (101 MHz, $CDCl_3$) δ 138.45, 136.70, 133.82, 133.52, 131.87, 130.72, 130.05, 128.96, 128.89, 128.77, 128.73, 128.66, 128.12, 127.07, 79.48, 16.63.HRMS (ESI/$[M+H]^+$) Calcd. for: $C_{22}H_{21}O_2S$ 349.1262,found 349.1253

(3-(phenylsulfonyl)prop-1-ene-1,1-diyl)dibenzene (3ae)

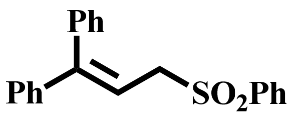

Following the general procedure, the reaction was conducted at 30 °C for 48h. **3ae** was isolated as white solid. Mp:107-109 °C.^{1}H NMR (400 MHz, $CDCl_3$) δ = 7.79 (d, *J* = 7.5, 2H), 7.64 (t, *J* = 7.5, 1H), 7.50 (t, *J* = 7.8, 2H), 7.32 – 7.20 (m, 6H), 7.17-7.12 (m, 2H), 6.66 (d, *J* = 7.8, 2H), 6.14 (t, *J* = 7.9, 1H), 3.92 (d, *J* = 7.9, 2H).^{13}C NMR (101 MHz, $CDCl_3$) δ = 149.78, 140.75, 138.64, 137.76, 133.67, 129.21, 129.12, 128.52, 128.39, 128.33, 128.29, 127.79, 127.42, 114.04, 57.53.HRMS (ESI/$[M+H]^+$) Calcd. for: $C_{21}H_{19}O_2S$ 335.1106, found 335.1113.

(E)-(3-(cyclopropylsulfonyl)prop-1-ene-1,3-diyl)dibenzene (3af)

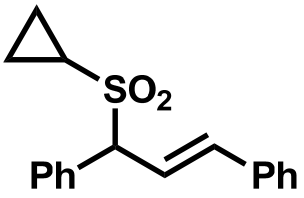

Following the general procedure, the reaction was conducted at 30 °C for 24h. **3af** was isolated as white solid. Mp:159-161 °C. ^{1}H NMR (400 MHz, $CDCl_3$) δ = 7.56 (dd, *J* = 7.7, 1.8 Hz, 2H), 7.48 – 7.36 (m, 5H), 7.36 – 7.23 (m, 3H), 6.79 (d, *J* = 15.7 Hz, 1H), 6.66 (dd, *J* = 15.7, 9.1 Hz, 1H), 4.87 (dd, *J* = 9.1, 2.1 Hz, 1H), 2.24 (ddd, *J* = 8.0, 3.9, 1.6 Hz, 1H), 1.31 – 1.19 (m, 1H), 1.20 – 1.10 (m, 1H), 1.00 – 0.83 (m, 2H). ^{13}C NMR (101 MHz, $CDCl_3$) δ = 137.83, 135.89, 132.60, 129.72, 129.15, 129.08, 128.79, 128.68, 126.94, 120.39, 73.13, 28.12, 5.28, 5.09. HRMS (ESI/$[M+H]^+$) Calcd. for: $C_{18}H_{19}O_2S$ 299.1106, found 299.1110.

(E)-2-((1,3-diphenylallyl)sulfonyl)thiophene (3ag)

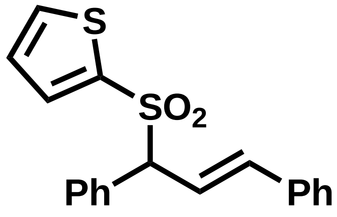

Following the general procedure, the reaction was conducted at 30 °C for 24h. **3ag** was isolated as white solid. Mp:195-197 °C. ^{1}H NMR (400 MHz, $CDCl_3$) δ = 7.63 (dd, *J* = 4.9, 1.3, 1H), 7.42 – 7.22 (m, 11H), 7.02 (ddd, *J* = 4.8, 3.8, 0.8, 1H), 6.66 – 6.54 (m, 2H), 4.93 (dd, *J* = 5.5, 2.8, 1H). ^{13}C NMR (101 MHz, $CDCl_3$) δ = 138.41, 138.01, 135.77, 135.39, 134.59, 132.30, 129.59, 129.08, 128.79, 128.64, 128.57, 127.51, 126.82, 119.81, 76.49. HRMS (ESI/$[M+H]^+$) Calcd. for: $C_{19}H_{17}O_2S_2$ 341.0664, found 341.0654.

(E)-(3-((3-chloropropyl)sulfonyl)prop-1-ene-1,3-diyl)dibenzene (3ah)

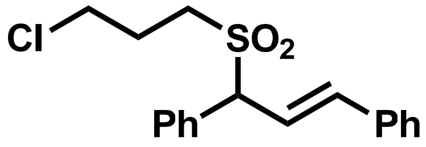

Following the general procedure, the reaction was conducted at 30 °C for 24h. **3ah** was isolated as white solid. Mp:115-117 °C. ^{1}H NMR (400 MHz, $CDCl_3$) δ 7.53 (dd, *J* = 7.9, 1.7 Hz, 2H), 7.45 – 7.38 (m, 5H), 7.35 – 7.24 (m, 3H), 6.77 (d, *J* = 15.7 Hz, 1H), 6.62 (dd, *J* = 15.7, 9.2 Hz, 1H), 4.89 (d, *J* = 9.1 Hz, 1H), 3.68 (t, *J* = 5.9 Hz, 2H), 3.13 – 2.94 (m, 2H), 2.07 – 1.97 (m, 2H). ^{13}C NMR (101 MHz,

CDCl$_3$) δ = 137.99, 135.56, 131.98, 129.49, 129.23, 129.14, 128.70, 126.88, 120.06, 72.55, 60.53, 47.60, 24.77. HRMS (ESI/[M+H]$^+$) Calcd. for: $C_{18}H_{20}O_2SCl$ 335.0873, found 335.0872.

(E)-5-(4,4-dimethyl-1-(phenylsulfonyl)pent-2-en-1-yl)benzo[d][1,3]dioxole (3ai)

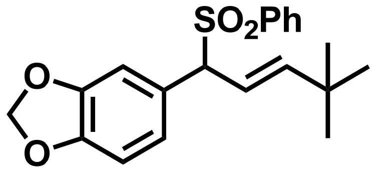

Following the general procedure, the reaction was conducted at 30 °C for 30h. **3ai** was isolated as colorless oil. ^{1}H NMR (400 MHz, CDCl$_3$) δ = 7.77 – 7.70 (m, 2H), 7.61 – 7.55 (m, 1H), 7.51 – 7.42 (m, 2H), 6.90 (d, *J* = 1.1, 1H), 6.74 (d, *J* = 1.0, 2H), 5.95 (q, *J* = 1.5, 2H), 5.67 (dd, *J* = 15.4, 9.5, 1H), 5.47 (dd, *J* = 15.4, 0.7, 1H), 4.52 (d, *J* = 9.4, 1H), 0.89 (s, 9H). ^{13}C NMR (101 MHz, CDCl$_3$) δ = 150.89, 148.24, 147.96, 137.68, 133.54, 129.38, 128.74, 125.64, 123.88, 116.50, 109.91, 108.48, 101.43, 74.79, 33.62, 29.05. HRMS (ESI/[M+H]$^+$) Calcd. for: $C_{20}H_{23}O_4S$ 359.1312, found 359.1324.

(E)-(2-((1,3-diphenylallyl)sulfonyl)ethyl)-l4-azane, 4-methylbenzenesulfonate salt (4)

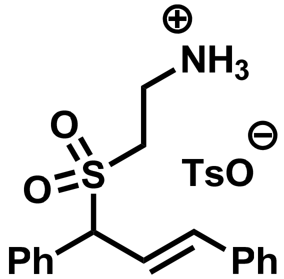

Following the general procedure, TsOH (2.5 equivalents) was added, otherwise no reaction occurred. The reaction was conducted at 30 °C for 48h. **4** was isolated as white solid. Mp: 94-96 °C. ^{1}H NMR (400 MHz, DMSO-*d*$_6$) δ = 7.61 (d, *J* = 6.8 Hz, 2H), 7.57 (d, *J* = 7.3 Hz, 2H), 7.54 – 7.49 (m, 2H), 7.47 – 7.41 (m, 3H), 7.40 – 7.34 (m, 2H), 7.34 – 7.28 (m, 1H), 7.13 (d, *J* = 7.5 Hz, 2H), 6.93 (d, *J* = 15.6 Hz, 1H), 6.76 (dd, *J* = 15.6, 9.8 Hz, 1H), 5.59 (d, *J* = 9.6, 1H), 3.75 (brs, 3H), 3.45 – 3.36 (m, 2H), 3.16 – 3.12 (m, 2H), 2.29 (s, 3H). ^{13}C NMR (101 MHz, DMSO) δ = 145.95, 138.03, 136.02, 132.46, 130.36, 129.31, 129.20, 129.06, 128.58, 127.40, 125.97, 121.33, 71.23, 48.46, 33.47, 21.26. HRMS (ESI/[M+H]$^+$) Calcd. for: $C_{24}H_{28}NO_5S_2$ 474.1403, found 474.1399.

(2*R*)-2-amino-3-(((E)-1,3-diphenylallyl)sulfonyl)propanoic acid (5)

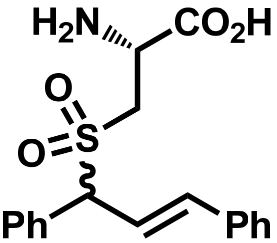

Following the general procedure, the reaction was conducted at 30 °C for 12h. **5** was isolated as white solid (dr = 76/24). Mp: over 300 °C. The major isomer ^{1}H NMR (400 MHz, DMSO-*d*$_6$) δ 7.99 (s, 2H), 7.64 – 7.60 (m, 2H), 7.58 – 7.54 (m, 2H), 7.47 – 7.41 (m, 3H), 7.41 – 7.35 (m, 2H), 7.34 – 7.29 (m, 1H), 6.96 (d, *J* = 15.7 Hz, 1H), 6.75 (dd, *J* = 15.7, 9.9 Hz, 1H), 5.87 (d, *J* = 9.8 Hz, 1H), 3.91 (dd, *J* =

14.8, 3.3 Hz, 1H), 3.76 (dd, J = 9.1, 3.3 Hz, 1H). ^{13}C NMR (101 MHz, DMSO-D_6) δ = 166.83, 137.37, 135.56, 131.71, 129.95, 129.81, 128.60, 128.49, 126.78, 121.39, 69.90, 50.56, 47.86. HRMS (ESI/[M+H]$^+$) Calcd. for: $C_{18}H_{20}NO_4S$ 346.1113, found 346.1116.

(E)-5-chloro-3-(4-((1,3-diphenylallyl)sulfonyl)phenyl)-6'-methyl-2,3'-bipyridine (6)

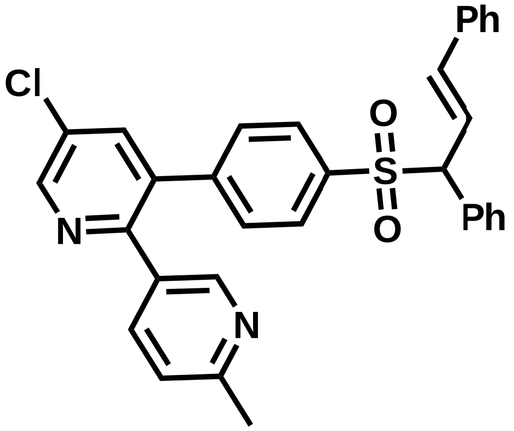

Following the general procedure, 6.0 equivalents HCl (37% in water) was added. The reaction was conducted at 30 °C for 72h. **6** was isolated as white solid. Mp:201-202 °C. ^{1}H NMR (400 MHz, $CDCl_3$) δ = 8.70 (d, J=2.0, 1H), 8.39 (s, 1H), 7.67 (d, J = 2.1, 1H), 7.60 (d, J = 8.2, 2H), 7.44 (dd, J = 8.0, 1.9, 1H), 7.41 – 7.28 (m, 10H), 7.24 (d, J = 8.2, 2H), 7.01 (d, J = 8.0, 1H), 6.62 – 6.57 (m, 2H), 4.84 (d, J = 6.8, 1H), 2.55 (s, 3H). ^{13}C NMR (101 MHz, $CDCl_3$) δ = 158.56, 152.39, 149.89, 148.37, 143.63, 138.52, 137.83, 137.17, 137.03, 135.76, 135.35, 132.04, 131.20, 131.06, 129.80, 129.73, 129.67, 129.09, 128.82, 128.70, 128.66, 126.79, 122.55, 119.43, 75.47, 29.71, 24.33. HRMS (ESI/[M+Na]$^+$) Calcd. for: $C_{32}H_{25}ClN_2O_2SNa$ 559.1223, found 559.1254.

Supplementary Table 4|1a.

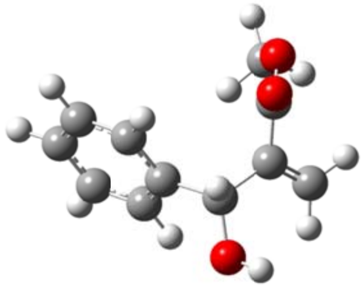

1a

Standard orientation:

Center Number	Atomic Number	Atomic Type	Coordinates (Angstroms) X	Y	Z
1	6	0	1.458849	0.685817	0.116478
2	6	0	2.069937	1.247796	1.165083
3	6	0	0.270787	1.320646	-0.613380
4	6	0	-1.007310	0.539758	-0.336432
5	6	0	-1.404486	-0.485223	-1.204321

6	6	0	-2.552617	-1.237034	-0.934637
7	6	0	-3.318253	-0.963415	0.201069
8	6	0	-2.930173	0.065902	1.065660
9	6	0	-1.778555	0.810327	0.802336
10	6	0	1.993225	-0.562103	-0.534834
11	8	0	2.282714	-0.569759	-1.713605
12	8	0	2.177345	-1.685780	0.194353
13	6	0	1.595630	-1.863429	1.500138
14	1	0	2.928997	0.788903	1.646280
15	1	0	1.732184	2.202042	1.559666
16	1	0	-0.817770	-0.695472	-2.095793
17	1	0	-2.850727	-2.028122	-1.617993
18	1	0	-4.215155	-1.541798	0.407909
19	1	0	-3.527588	0.291389	1.945709
20	1	0	-1.483921	1.616206	1.467815
21	1	0	1.515454	-2.944070	1.629673
22	1	0	2.249125	-1.449705	2.273049
23	1	0	0.605263	-1.407200	1.563261
24	8	0	0.062553	2.674044	-0.221020
25	1	0	0.815936	3.200897	-0.531948
26	1	0	0.485110	1.264634	-1.688414

Supplementary Table 5 | Sulfinic acid 2

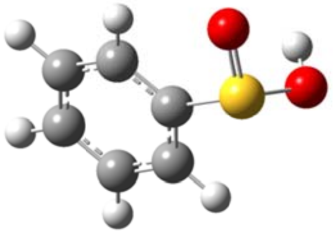

2

Standard orientation:

Center Number	Atomic Number	Atomic Type	Coordinates (Angstroms) X	Y	Z
1	6	0	0.848485	-1.205659	-0.304254
2	6	0	2.235158	-1.153572	-0.150916
3	6	0	2.863993	0.065465	0.128889
4	6	0	2.109131	1.235167	0.254831
5	6	0	0.719806	1.194239	0.100602
6	6	0	0.104519	-0.029241	-0.165494
7	16	0	-1.702207	-0.072819	-0.430999
8	8	0	-2.179121	1.298458	-0.069893

9	8	0	-2.080828	-1.155727	0.808724
10	1	0	0.352130	-2.149587	-0.515385
11	1	0	2.824096	-2.061588	-0.249524
12	1	0	3.944098	0.102369	0.244222
13	1	0	2.600407	2.180889	0.468001
14	1	0	0.110601	2.089325	0.188420
15	1	0	-1.802977	-0.776551	1.667645

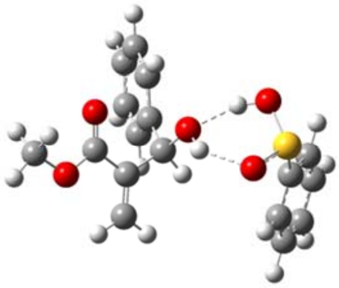

1a+2(hydrogen bonded)

Supplementary Table 6|1a+2(hydrogen bonded).

Standard orientation:

Center Number	Atomic Number	Atomic Type	Coordinates (Angstroms) X	Y	Z
1	6	0	-3.808450	0.799047	-0.339364
2	6	0	-4.294909	1.496116	0.768846
3	6	0	-4.249646	0.914398	2.040650
4	6	0	-3.714363	-0.366039	2.208679
5	6	0	-3.222204	-1.071417	1.105949
6	6	0	-3.264779	-0.476291	-0.156818
7	16	0	-2.725092	-1.454748	-1.603634
8	8	0	-1.890082	-0.277443	-2.416522
9	1	0	-3.830393	1.249059	-1.327785
10	1	0	-4.705881	2.493839	0.639505
11	1	0	-4.633330	1.459086	2.899383
12	1	0	-3.682602	-0.818737	3.196498
13	1	0	-2.799490	-2.065780	1.217183
14	1	0	-1.034526	-0.105868	-1.927409
15	8	0	-1.753630	-2.462443	-1.028307
16	1	0	-0.065735	-1.486591	-0.728815
17	8	0	0.346596	-0.614594	-0.916470
18	6	0	0.817678	-0.025211	0.301181
19	1	0	-0.026117	0.067361	0.999833
20	6	0	1.836132	-0.931046	0.988266

21	6	0	1.591729	-1.428923	2.209593
22	1	0	0.666234	-1.196695	2.731717
23	1	0	2.301027	-2.072467	2.717882
24	6	0	3.102472	-1.258005	0.255432
25	8	0	3.405971	-0.812165	-0.832362
26	8	0	3.890677	-2.121098	0.941225
27	6	0	5.125509	-2.482082	0.296582
28	1	0	5.743556	-1.595217	0.132780
29	1	0	5.616144	-3.173405	0.982503
30	1	0	4.926428	-2.965523	-0.663553
31	6	0	1.324294	1.381102	0.011644
32	6	0	1.359948	2.314781	1.056058
33	6	0	1.743148	1.771247	-1.265553
34	6	0	1.813058	3.617013	0.834033
35	1	0	1.028726	2.023833	2.051540
36	6	0	2.187733	3.076661	-1.490419
37	1	0	1.732778	1.048122	-2.072790
38	6	0	2.227291	4.002473	-0.444085
39	1	0	1.834018	4.329550	1.655007
40	1	0	2.510551	3.367838	-2.486936
41	1	0	2.575988	5.016578	-0.622960

Supplementary Table 7|TS.

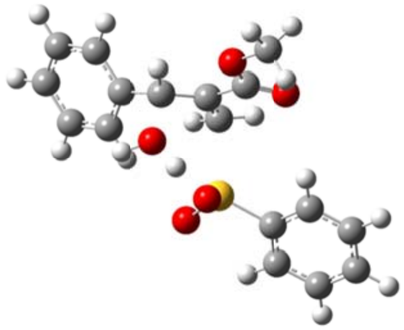

TS

Standard orientation:

Center Number	Atomic Number	Atomic Type	Coordinates (Angstroms) X	Y	Z
1	6	0	-0.931102	1.080397	-0.535058
2	6	0	-0.255851	0.079819	-1.244055
3	6	0	-3.037709	-0.302624	0.023770
4	6	0	-2.675711	-1.660314	0.090864
5	6	0	-3.614305	-2.653801	-0.199320
6	6	0	-4.918980	-2.311222	-0.564301

7	6	0	-5.289393	-0.964909	-0.625843
8	6	0	-4.358358	0.029632	-0.322780
9	6	0	-0.289289	2.400804	-0.492185
10	8	0	0.799537	2.664315	-0.989001
11	8	0	-1.029911	3.341551	0.158121
12	6	0	-0.444573	4.650418	0.242842
13	1	0	0.523846	0.395593	-1.930678
14	1	0	-1.669691	-1.959387	0.374239
15	1	0	-3.318267	-3.697710	-0.140684
16	1	0	-5.644193	-3.088171	-0.791471
17	1	0	-6.304111	-0.688176	-0.899586
18	1	0	-4.654032	1.075526	-0.364172
19	1	0	-1.166748	5.254215	0.794230
20	1	0	-0.280116	5.063113	-0.756335
21	1	0	0.508877	4.609560	0.776616
22	6	0	-2.090364	0.825042	0.301844
23	1	0	-2.615988	1.737717	0.562608
24	1	0	-0.763102	-0.843451	-1.506936
25	8	0	0.868525	-0.251481	1.574403
26	1	0	-0.437182	0.165163	1.772961
27	8	0	-1.470133	0.514466	1.885911
28	1	0	-1.991372	-0.183879	2.323663
29	8	0	0.821857	-2.488646	0.320926
30	16	0	1.122979	-1.020775	0.254154
31	6	0	2.911346	-0.839894	0.028884
32	6	0	3.695340	-1.989438	-0.073556
33	6	0	3.456826	0.441072	-0.094003
34	6	0	5.071501	-1.849236	-0.274692
35	1	0	3.228757	-2.965839	0.014850
36	6	0	4.832067	0.564965	-0.301889
37	1	0	2.821284	1.321104	-0.040275
38	6	0	5.637936	-0.576063	-0.388075
39	1	0	5.698325	-2.734523	-0.344400
40	1	0	5.272960	1.553583	-0.398746
41	1	0	6.707799	-0.471568	-0.549921

Supplementary Table 8|Z-3a.

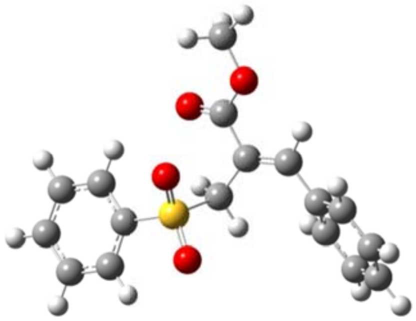

***Z*-3a**

Standard orientation:

Center Number	Atomic Number	Atomic Type	Coordinates (Angstroms) X	Y	Z
1	6	0	2.220845	0.993826	-0.187162
2	6	0	0.891724	1.056436	-0.430778
3	6	0	-0.007070	-0.112475	-0.698114
4	6	0	3.122227	-0.171107	-0.235513
5	6	0	3.097599	-1.116429	-1.277410
6	6	0	3.995508	-2.184420	-1.291903
7	6	0	4.932986	-2.330586	-0.265613
8	6	0	4.982349	-1.387311	0.765242
9	6	0	4.096598	-0.310270	0.771057
10	1	0	-0.744564	0.116160	-1.470461
11	1	0	0.525163	-1.030200	-0.939232
12	1	0	2.401397	-0.992931	-2.102027
13	1	0	3.967320	-2.899090	-2.110408
14	1	0	5.627953	-3.166070	-0.275590
15	1	0	5.714027	-1.487491	1.562615
16	1	0	4.141880	0.423423	1.572433
17	1	0	2.698040	1.928987	0.097701
18	6	0	0.183193	2.372471	-0.417139
19	8	0	-0.947631	2.523831	-0.850941
20	8	0	0.916439	3.384028	0.086787
21	6	0	0.271340	4.670395	0.122173
22	1	0	-0.630788	4.622098	0.737229
23	1	0	1.004157	5.345883	0.564354
24	1	0	0.007224	4.992941	-0.888515
25	16	0	-0.981464	-0.559640	0.839741
26	6	0	-2.654594	-0.861029	0.232204
27	6	0	-3.457861	0.227246	-0.121941
28	6	0	-3.115382	-2.175892	0.145452
29	6	0	-4.754180	-0.017241	-0.578674

30	1	0	-3.072056	1.239136	-0.044098
31	6	0	-4.417604	-2.405008	-0.308004
32	1	0	-2.467280	-2.994233	0.442602
33	6	0	-5.233056	-1.329098	-0.670154
34	1	0	-5.391501	0.817427	-0.857922
35	1	0	-4.793542	-3.422512	-0.373695
36	1	0	-6.245152	-1.511724	-1.022125
37	8	0	-0.446421	-1.844690	1.332234
38	8	0	-1.018211	0.618753	1.725535

Supplementary Table 9|H_2O.

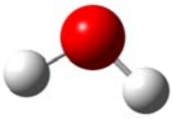

Standard orientation:

Center Number	Atomic Number	Atomic Type	Coordinates (Angstroms) X	Y	Z
1	8	0	0.000000	0.117254	0.000000
2	1	0	0.771370	-0.469015	0.000000
3	1	0	-0.771370	-0.469016	0.000000

Supplementary Table 10|TS'.

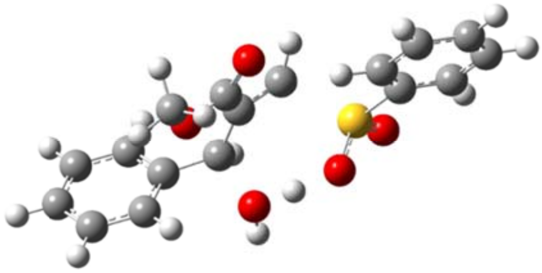

TS'

Standard orientation:

Standard orientation:

Center Number	Atomic Number	Atomic Type	Coordinates (Angstroms) X	Y	Z
1	6	0	-0.753372	-0.028119	-0.767942
2	6	0	0.369645	-0.602125	-1.424950
3	6	0	-0.694595	1.410656	-0.537020

4	8	0	0.244384	2.127184	-0.881085
5	8	0	-1.776702	1.917693	0.109423
6	6	0	-1.764573	3.334053	0.339766
7	1	0	0.982048	0.069453	-2.022332
8	1	0	-2.706758	3.550603	0.845817
9	1	0	-1.704202	3.878193	-0.606900
10	1	0	-0.916035	3.615676	0.969983
11	6	0	-1.722765	-0.932178	-0.173032
12	1	0	0.265823	-1.601051	-1.849475
13	8	0	1.223383	-0.934275	1.382629
14	1	0	-0.102640	-1.110577	1.419676
15	8	0	-1.218292	-1.212517	1.407565
16	1	0	-1.478367	-2.102028	1.714116
17	8	0	2.224419	-2.683066	-0.204371
18	16	0	1.868355	-1.250944	0.011998
19	6	0	3.400174	-0.291211	0.042283
20	6	0	4.612638	-0.978116	-0.038381
21	6	0	3.339336	1.103649	0.104209
22	6	0	5.801383	-0.243885	-0.027624
23	1	0	4.613727	-2.061906	-0.100842
24	6	0	4.536710	1.822376	0.106468
25	1	0	2.382701	1.619241	0.128049
26	6	0	5.763331	1.151858	0.045906
27	1	0	6.754736	-0.763184	-0.079338
28	1	0	4.508714	2.907755	0.151256
29	1	0	6.690475	1.719313	0.048000
30	6	0	-3.197516	-0.629272	-0.052088
31	6	0	-3.797123	-0.080577	1.087690
32	6	0	-4.002080	-0.954823	-1.154069
33	6	0	-5.174934	0.147312	1.117621
34	1	0	-3.184074	0.176055	1.943841
35	6	0	-5.377521	-0.715433	-1.128290
36	1	0	-3.548031	-1.387484	-2.043312
37	6	0	-5.969657	-0.164612	0.011200
38	1	0	-5.627171	0.573925	2.009560
39	1	0	-5.984378	-0.967561	-1.994113
40	1	0	-7.041234	0.015592	0.038253
41	1	0	-1.591011	-1.934388	-0.587956

Supplementary Table 11|E-3a.

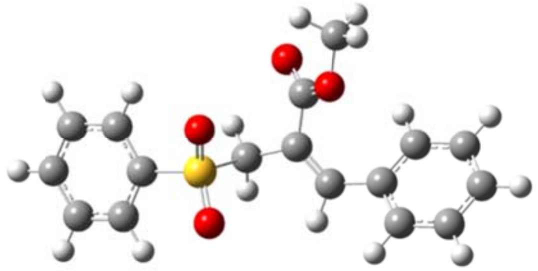

***E*-3a**

Standard orientation:

Center Number	Atomic Number	Atomic Type	Coordinates (Angstroms) X	Y	Z
1	6	0	1.624645	-0.888727	-0.524255
2	6	0	0.750020	0.141344	-0.568988
3	6	0	-0.702841	-0.114230	-0.865220
4	1	0	-1.156394	0.743357	-1.368039
5	1	0	-0.863869	-1.021822	-1.451218
6	6	0	1.068791	1.589366	-0.348970
7	8	0	0.510994	2.489338	-0.953941
8	8	0	1.992184	1.792418	0.602326
9	6	0	2.307271	3.166430	0.894627
10	1	0	1.411915	3.691466	1.237067
11	1	0	3.057660	3.124893	1.684191
12	1	0	2.704992	3.665462	0.006438
13	16	0	-1.656364	-0.368954	0.700112
14	6	0	-3.376113	-0.329307	0.156686
15	6	0	-4.026436	0.903732	0.055201
16	6	0	-4.021797	-1.527367	-0.158225
17	6	0	-5.352527	0.932930	-0.381270
18	1	0	-3.506506	1.816095	0.331380
19	6	0	-5.349178	-1.484550	-0.592196
20	1	0	-3.497346	-2.470802	-0.042811
21	6	0	-6.010707	-0.257900	-0.706368
22	1	0	-5.872677	1.883681	-0.459751
23	1	0	-5.866971	-2.408880	-0.833681
24	1	0	-7.043806	-0.230069	-1.042871
25	8	0	-1.362685	-1.734426	1.179093
26	8	0	-1.420884	0.800894	1.566557
27	1	0	1.186135	-1.880997	-0.633818
28	6	0	3.088429	-0.898686	-0.372446
29	6	0	3.681183	-1.961836	0.333840
30	6	0	3.927389	0.063330	-0.963045

31	6	0	5.065757	-2.034488	0.488932
32	1	0	3.046035	-2.724047	0.779427
33	6	0	5.312031	-0.018949	-0.823037
34	1	0	3.495141	0.864436	-1.555276
35	6	0	5.886704	-1.062087	-0.089041
36	1	0	5.503023	-2.854212	1.053059
37	1	0	5.945401	0.727967	-1.295030
38	1	0	6.966490	-1.123096	0.019496

Supplementary Table 12|2”.

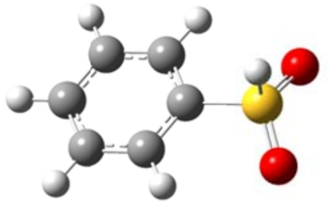

2’’

Standard orientation:

Center Number	Atomic Number	Atomic Type	Coordinates (Angstroms) X	Y	Z
1	6	0	0.792236	1.221433	0.041293
2	6	0	2.186975	1.213799	-0.029948
3	6	0	2.881030	-0.000064	-0.062258
4	6	0	2.186932	-1.213896	-0.029969
5	6	0	0.792201	-1.221435	0.041240
6	6	0	0.113902	0.000025	0.077388
7	16	0	-1.677186	-0.000002	0.196249
8	8	0	-2.181663	1.288645	-0.297878
9	8	0	-2.182080	-1.288521	-0.297808
10	1	0	0.235031	2.153018	0.048943
11	1	0	2.729758	2.154272	-0.065956
12	1	0	3.966337	-0.000076	-0.119261
13	1	0	2.729656	-2.154402	-0.065994
14	1	0	0.234967	-2.153001	0.048833
15	1	0	-1.870486	0.000056	1.552464

Supplementary Table 13| 2’+H2O (hydrogen bonded).

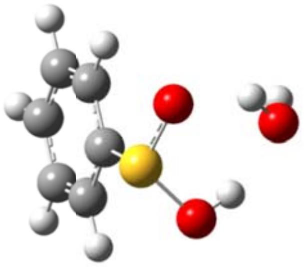

2’

Standard orientation:

Center Number	Atomic Number	Atomic Type	Coordinates (Angstroms) X	Y	Z
1	6	0	1.185434	-1.238449	0.217995
2	6	0	2.516128	-0.998314	0.566962
3	6	0	3.086737	0.259110	0.339150
4	6	0	2.327399	1.281049	-0.237687
5	6	0	0.993785	1.050969	-0.590363
6	6	0	0.432355	-0.203996	-0.346752
7	16	0	-1.279220	-0.524472	-0.902620
8	8	0	-1.843962	-1.306591	0.446121
9	1	0	0.733015	-2.208872	0.401964
10	1	0	3.106555	-1.791582	1.017862
11	1	0	4.123760	0.440070	0.609537
12	1	0	2.771848	2.256563	-0.417126
13	1	0	0.387486	1.833249	-1.037833
14	1	0	-1.943644	-0.632137	1.176601
15	8	0	-1.905102	0.850416	-0.989291
16	1	0	-2.259949	1.391654	0.859019
17	8	0	-2.261914	1.036689	1.775715
18	1	0	-1.614745	1.556287	2.275702

Supplementary Table 14 | 1a+2’ (hydrogen bonded).

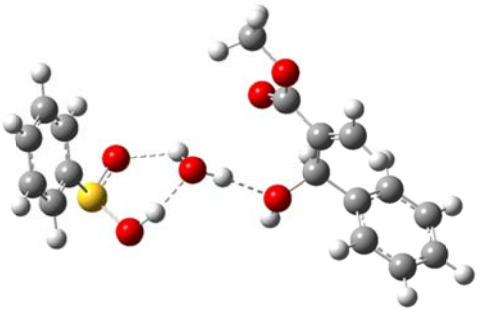

1a+2’ (hydrogen bonded)

Standard orientation:

Center Number	Atomic Number	Atomic Type	Coordinates (Angstroms) X	Y	Z
1	8	0	-2.476976	-2.335960	-0.039277
2	1	0	-1.871269	-1.580256	0.247507
3	8	0	-2.786261	-0.705369	-2.023248
4	1	0	-1.256899	0.092880	-0.737909
5	6	0	2.691384	1.377296	0.291370
6	6	0	3.385467	1.821485	1.346745
7	1	0	4.196789	1.241131	1.774626
8	1	0	3.160662	2.781174	1.799182
9	6	0	1.606762	2.199603	-0.335708
10	8	0	1.118931	1.949660	-1.423907
11	8	0	1.234033	3.252151	0.417384
12	6	0	0.182287	4.072531	-0.127671
13	1	0	-0.724827	3.478046	-0.261373
14	1	0	0.023348	4.860014	0.609348
15	1	0	0.488027	4.495123	-1.088352
16	6	0	4.252162	-0.626623	-0.068108
17	6	0	4.386815	-1.473574	1.040712
18	6	0	5.366380	-0.395451	-0.886845
19	6	0	5.616651	-2.073616	1.326876
20	1	0	3.523092	-1.666519	1.670614
21	6	0	6.597080	-0.989476	-0.598557
22	1	0	5.269518	0.252961	-1.755646
23	6	0	6.725006	-1.831297	0.510546
24	1	0	5.707450	-2.731329	2.187718
25	1	0	7.451615	-0.803212	-1.244095
26	1	0	7.680204	-2.300115	0.732609
27	6	0	2.928726	0.041240	-0.395867
28	1	0	2.877368	0.222111	-1.476517
29	8	0	1.807914	-0.796967	-0.023024
30	1	0	1.830885	-1.598636	-0.572390
31	8	0	-0.958866	-0.144156	0.162947
32	1	0	0.008778	-0.288610	0.096038
33	16	0	-3.505812	-1.743082	-1.193592
34	6	0	-4.618712	-0.770705	-0.116252
35	6	0	-5.226448	-1.386767	0.981836
36	6	0	-4.919119	0.545861	-0.465849
37	6	0	-6.134778	-0.659636	1.753732
38	1	0	-4.977152	-2.411877	1.242543
39	6	0	-5.829152	1.266836	0.313739
40	1	0	-4.432058	0.993697	-1.327150

41	6	0	-6.436802	0.665511	1.419428
42	1	0	-6.604486	-1.125272	2.616334
43	1	0	-6.064163	2.296254	0.055277
44	1	0	-7.146344	1.227560	2.021333

Supplementary Table 15| TS".

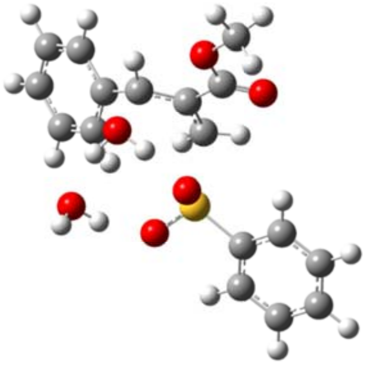

TS"

Standard orientation:

Center Number	Atomic Number	Atomic Type	Coordinates (Angstroms) X	Y	Z
1	6	0	-0.937327	1.223399	-0.617494
2	6	0	-0.191273	0.349174	-1.400903
3	6	0	-3.018207	-0.267380	-0.243189
4	6	0	-2.617115	-1.598708	-0.453351
5	6	0	-3.543959	-2.555975	-0.866805
6	6	0	-4.880366	-2.205158	-1.079622
7	6	0	-5.293666	-0.889180	-0.858142
8	6	0	-4.371591	0.066742	-0.430788
9	6	0	-0.381757	2.586608	-0.465128
10	8	0	0.634692	2.985111	-1.013443
11	8	0	-1.127987	3.381359	0.346743
12	6	0	-0.632410	4.716391	0.542770
13	1	0	0.596181	0.768396	-2.017921
14	1	0	-1.594571	-1.906713	-0.262788
15	1	0	-3.219345	-3.582788	-1.011563
16	1	0	-5.596850	-2.956127	-1.402124
17	1	0	-6.332859	-0.607935	-1.006643
18	1	0	-4.699864	1.087907	-0.251497
19	1	0	-1.346286	5.192679	1.215972
20	1	0	-0.583894	5.250070	-0.410294
21	1	0	0.363480	4.689664	0.992897
22	6	0	-2.103557	0.840756	0.146488

23	1	0	-2.645232	1.695190	0.534211
24	1	0	-0.590719	-0.608201	-1.713304
25	8	0	1.132913	0.286937	1.401298
26	1	0	-0.499090	0.498073	1.753938
27	8	0	-1.500880	0.378460	1.816304
28	1	0	-1.588478	-0.600216	2.018475
29	8	0	0.901729	-2.095816	0.500708
30	16	0	1.302446	-0.653149	0.209891
31	6	0	3.091451	-0.691555	-0.085239
32	6	0	3.721326	-1.912065	-0.333884
33	6	0	3.788788	0.519005	-0.134721
34	6	0	5.090848	-1.919449	-0.615008
35	1	0	3.146892	-2.832047	-0.285916
36	6	0	5.155067	0.497669	-0.421893
37	1	0	3.272620	1.455710	0.055674
38	6	0	5.805240	-0.718491	-0.660801
39	1	0	5.597924	-2.863542	-0.797281
40	1	0	5.711851	1.430426	-0.456832
41	1	0	6.869021	-0.728841	-0.884144
42	8	0	-1.122566	-2.245297	2.369873
43	1	0	-0.324887	-2.332263	1.788403
44	1	0	-0.807182	-2.375778	3.276860

Supplementary Table 16| Computed Energies

Structure	E_0	H_{298}	G_{298}	TCGFE
**1a**	-651.845364	-651.830743	-651.887403	0.167012
**2**	-780.739956	-780.730776	-780.773704	0.075386
**1a+2** (hydrogen bonded)	-1432.611008	-1432.587051	-1432.669260	0.262885
**TS**	-1432.564153	-1432.541289	-1432.617341	0.265445
***Z*-3a**	-1356.200708	-1356.179379	-1356.252913	0.244525
**TS’**	-1432.557333	-1432.534446	-1432.611006	0.264062
***E*-3a**	-1356.198791	-1356.177396	-1356.251566	0.243766
**H_2O**	-76.401475	-76.397696	-76.419790	0.002782
**2’**	-857.154978	-857.143349	-857.192078	0.097632
**2’’**	-780.712054	-780.703543	-780.745228	0.076999
**1a+2’** (hydrogen bonded)	-1509.024369	-1508.997478	-1509.087920	0.282673
**TS’’**	-1508.983153	-1508.957728	-1509.039265	0.290251

E_0：Sum of electronic and zero-point Energies

H_{298}：Sum of electronic and thermal Enthalpies

G_{298}：Sum of electronic and thermal Free Energies

TCGFE：Thermal Correction to Gibbs Free Energy

Supplementary Figure 2 | ^{1}H NMR (400 MHz, $CDCl_3$) spectra for compound **3a**.

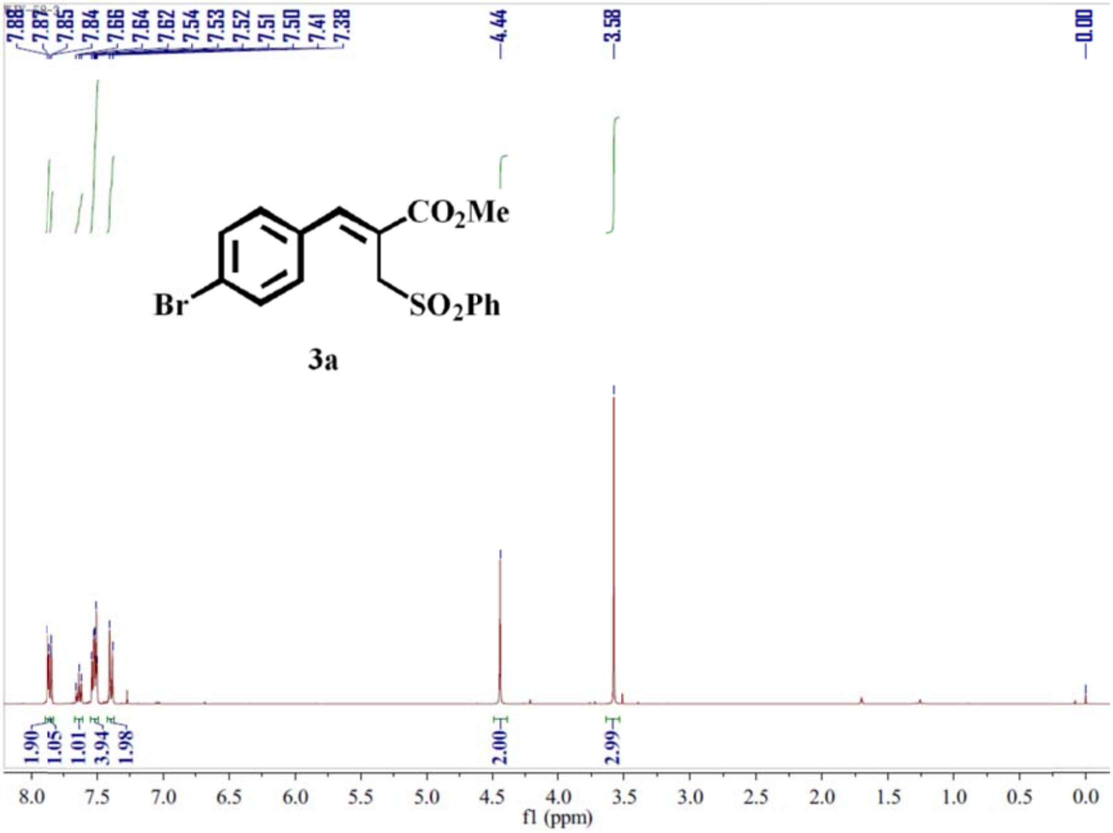

Supplementary Figure 3 | ^{13}C NMR (101 MHz, $CDCl_3$) spectra for compound **3a**.

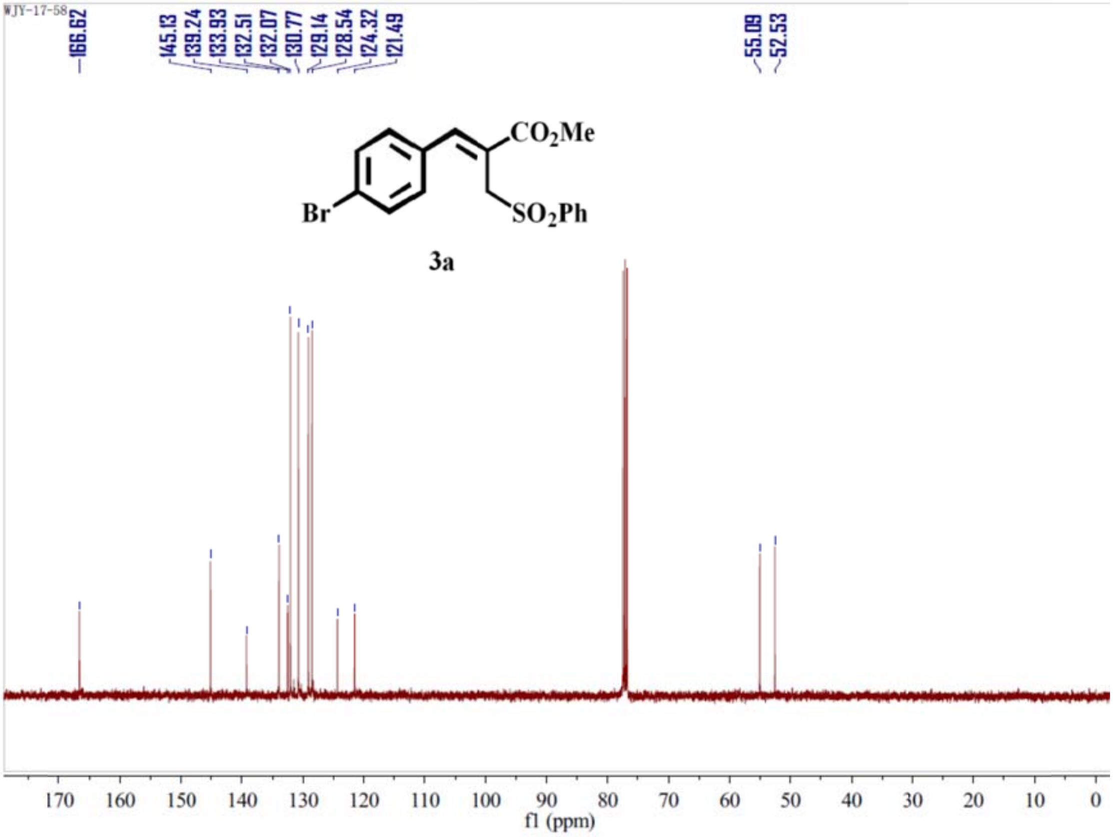

Supplementary Figure 4 | ^{1}H NMR (400 MHz, $CDCl_3$) spectra for compound **3b**.

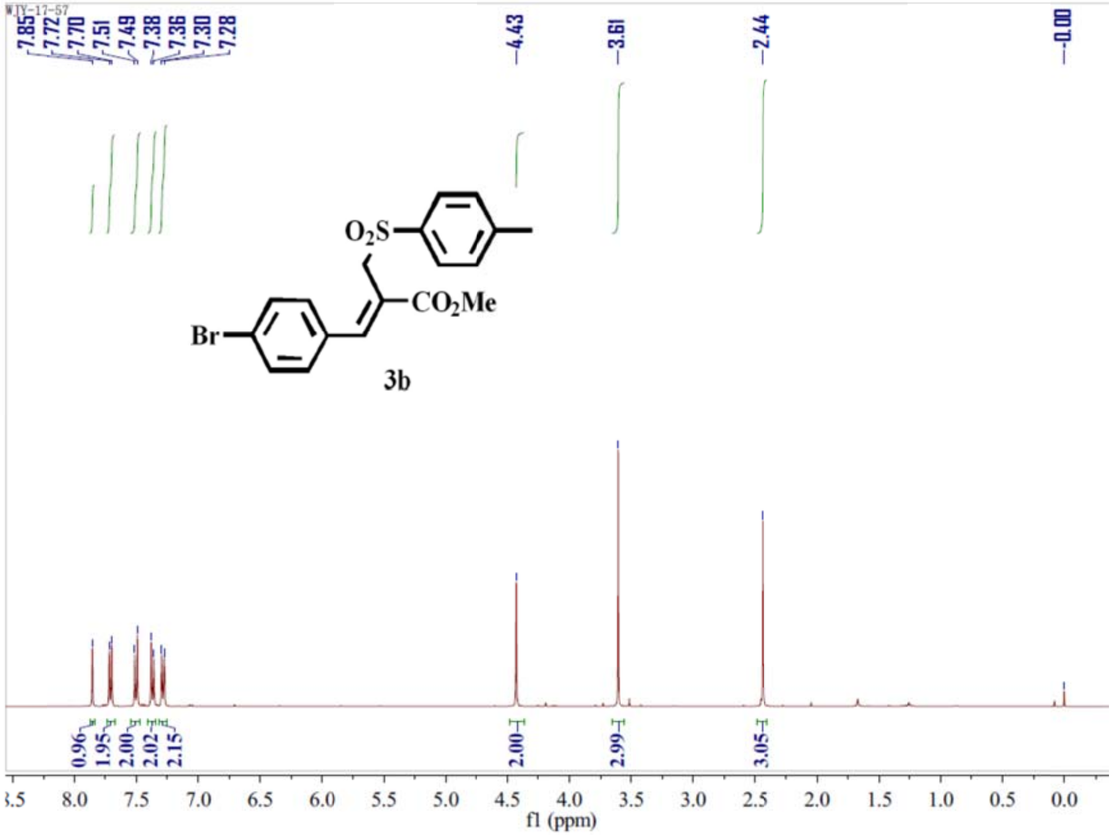

Supplementary Figure 5 | ^{13}C NMR (101 MHz, $CDCl_3$) spectra for compound **3b**.

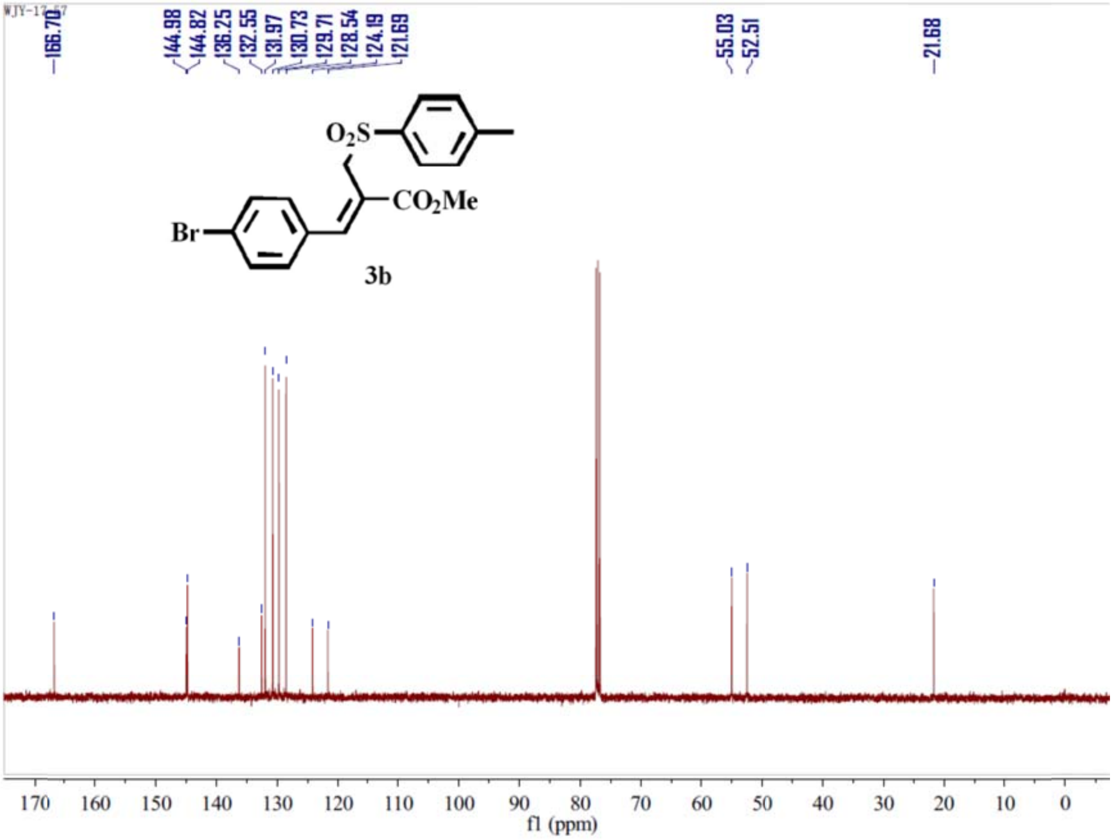

Supplementary Figure 6 | ^{1}H NMR (400 MHz, $CDCl_3$) spectra for compound **3c**.

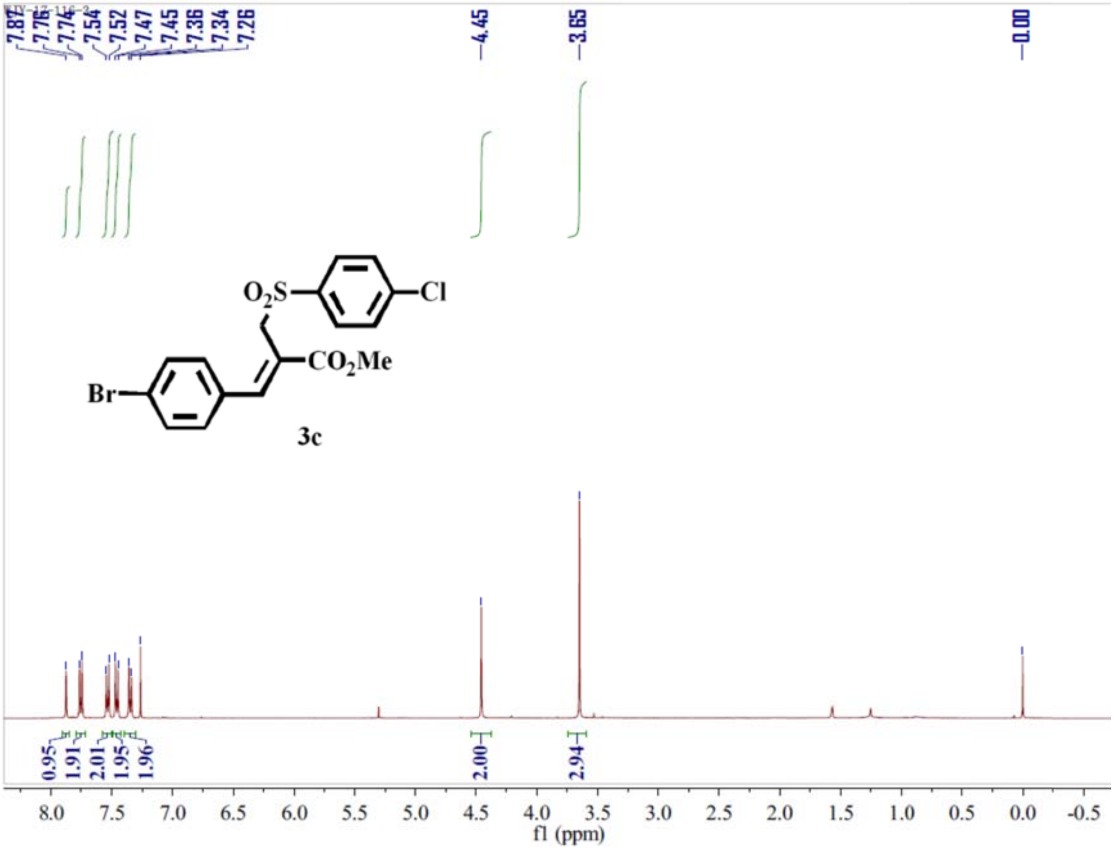

Supplementary Figure 7 | ^{13}C NMR (101 MHz, $CDCl_3$) spectra for compound **3c**.

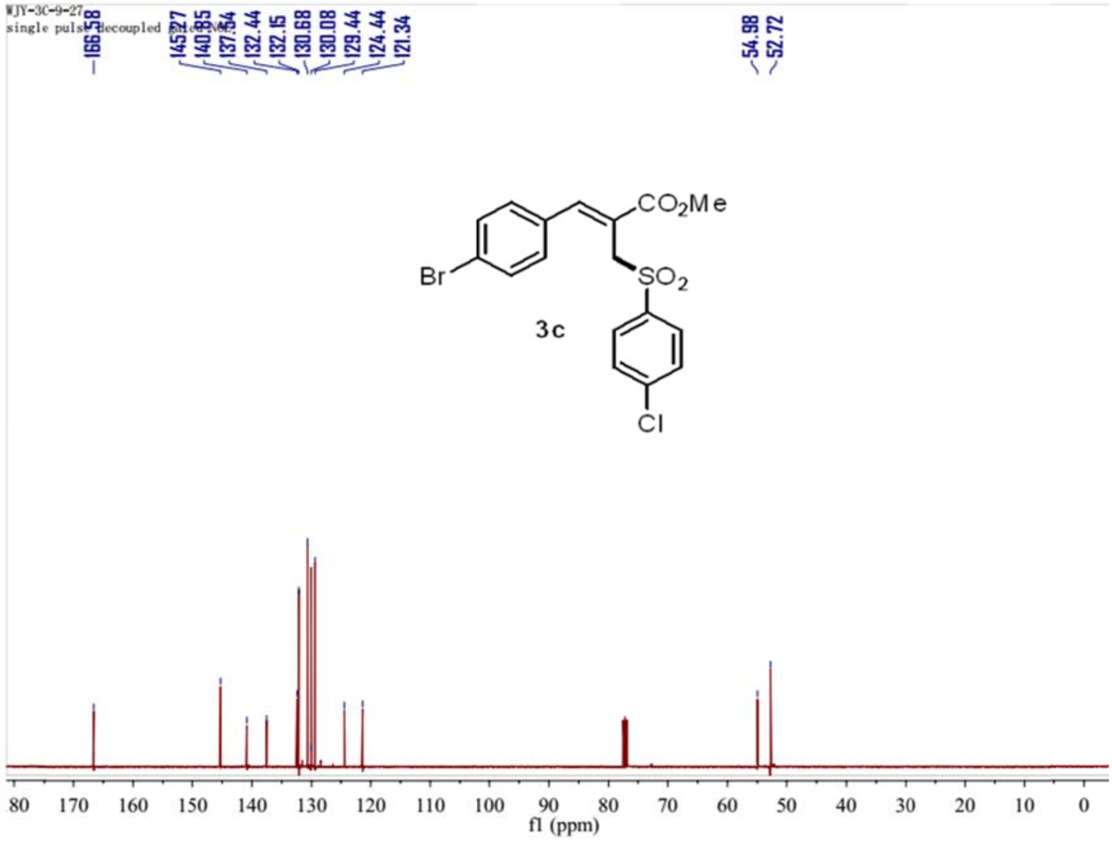

Supplementary Figure 8 | ^{1}H NMR (400 MHz, $CDCl_3$) spectra for compound **3d**.

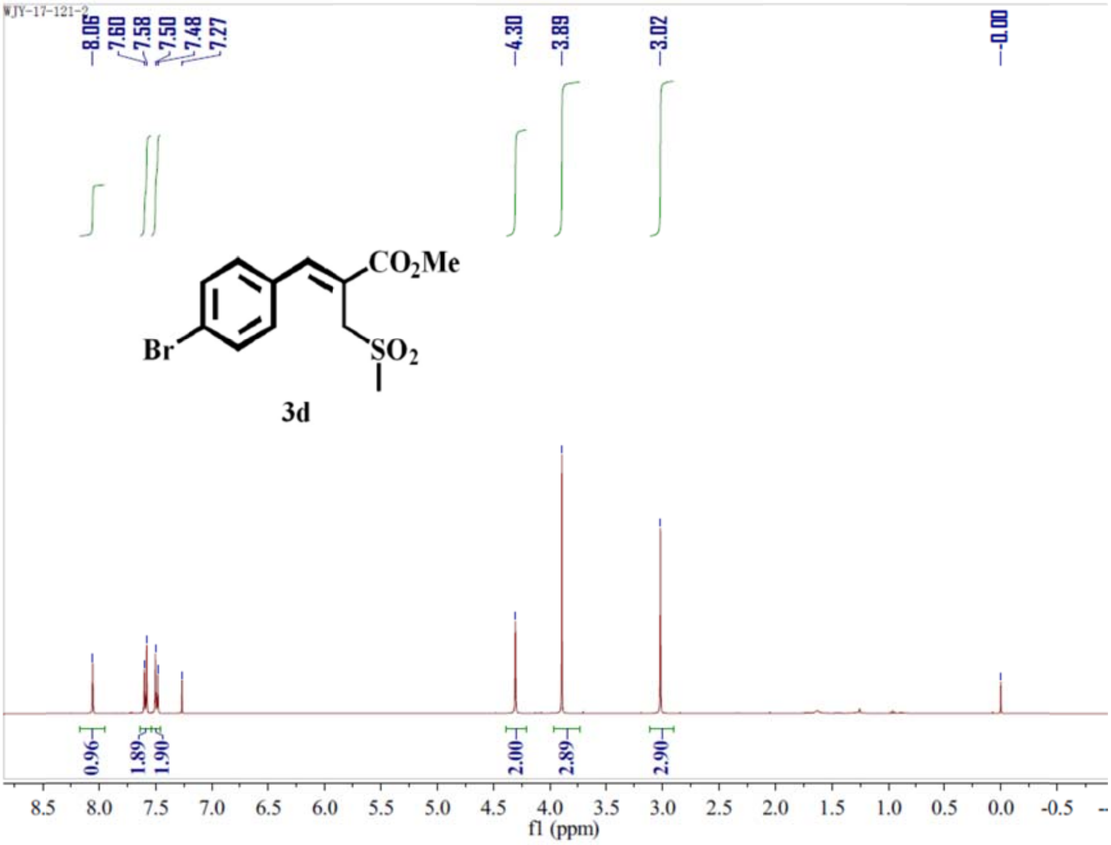

Supplementary Figure 9 | ^{13}C NMR (101 MHz, $CDCl_3$) spectra for compound **3d**.

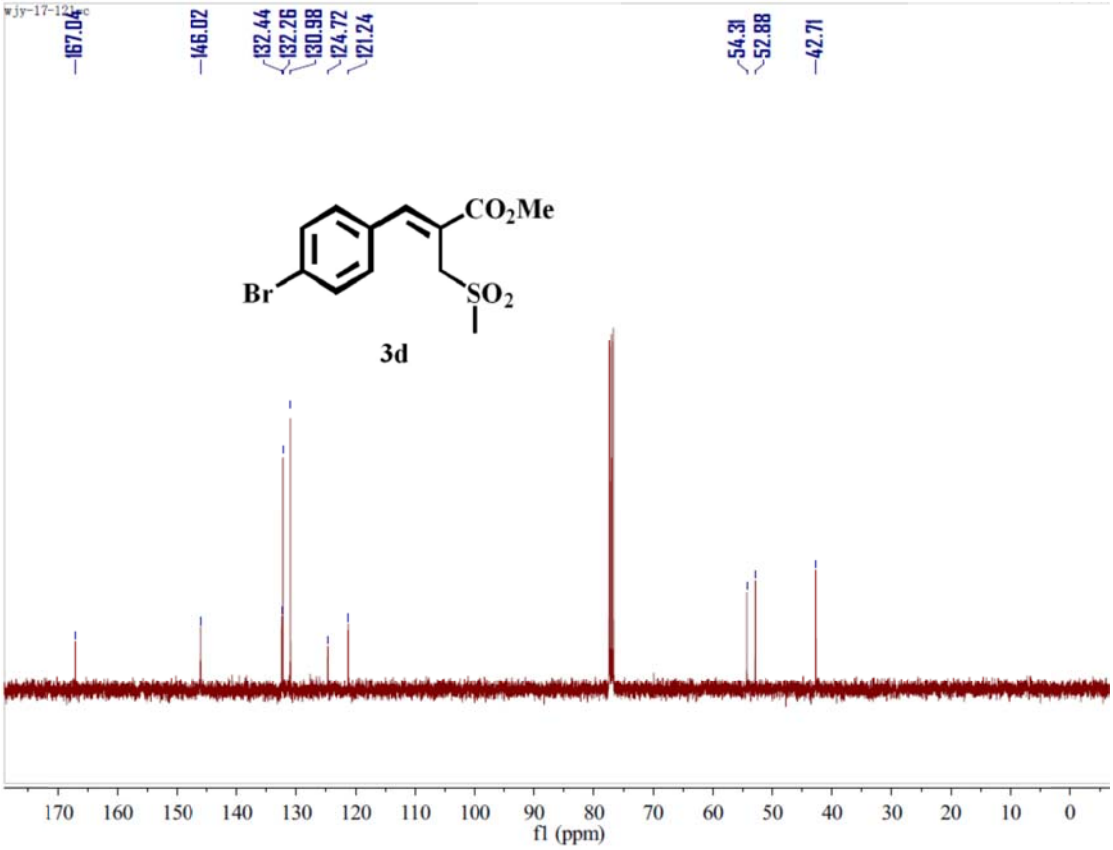

Supplementary Figure 10 | ^{1}H NMR (400 MHz, $CDCl_3$) spectra for compound **3e**.

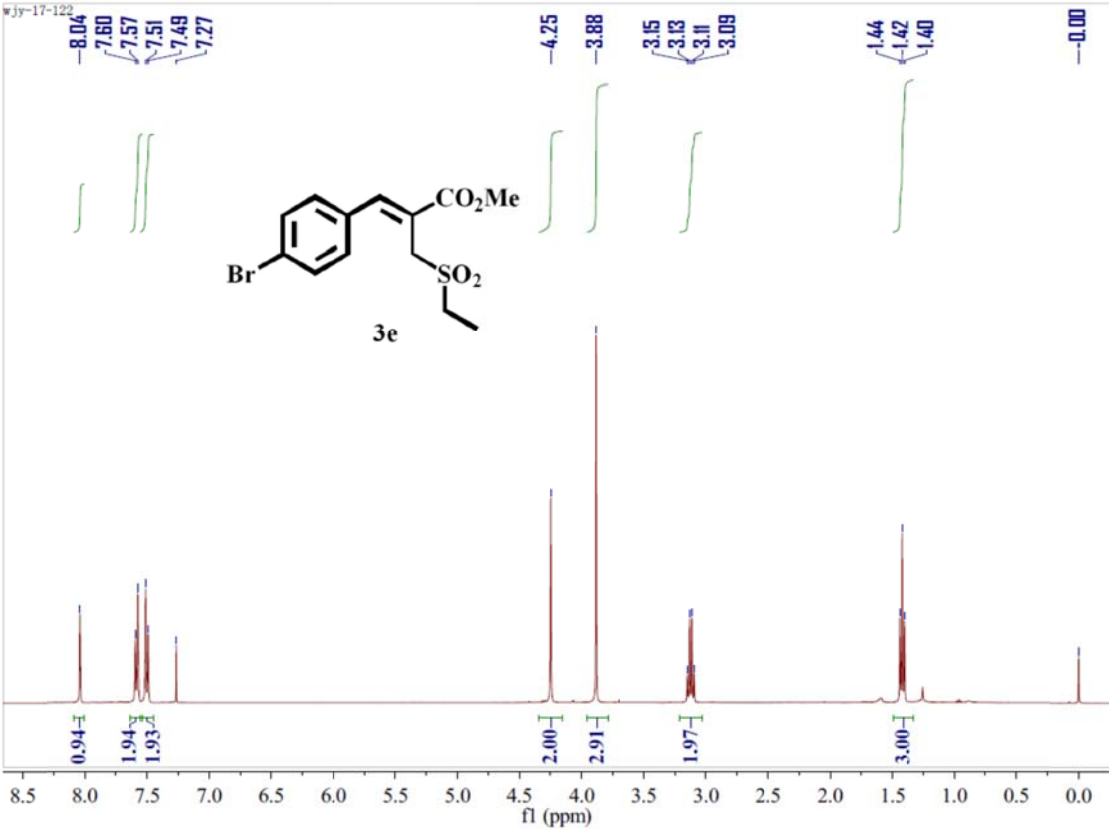

Supplementary Figure 11 | ^{13}C NMR (101 MHz, $CDCl_3$) spectra for compound **3e**.

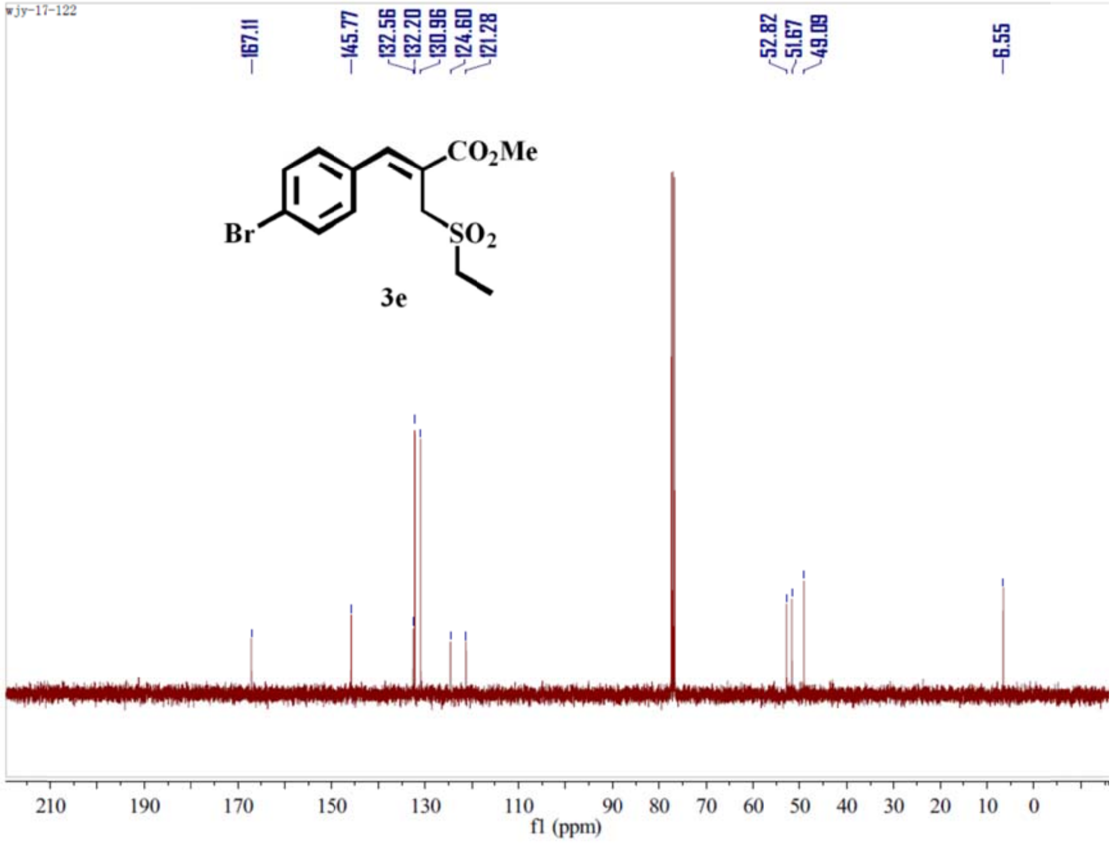

Supplementary Figure 12 | ^{1}H NMR (400 MHz, $CDCl_3$) spectra for compound **3f**.

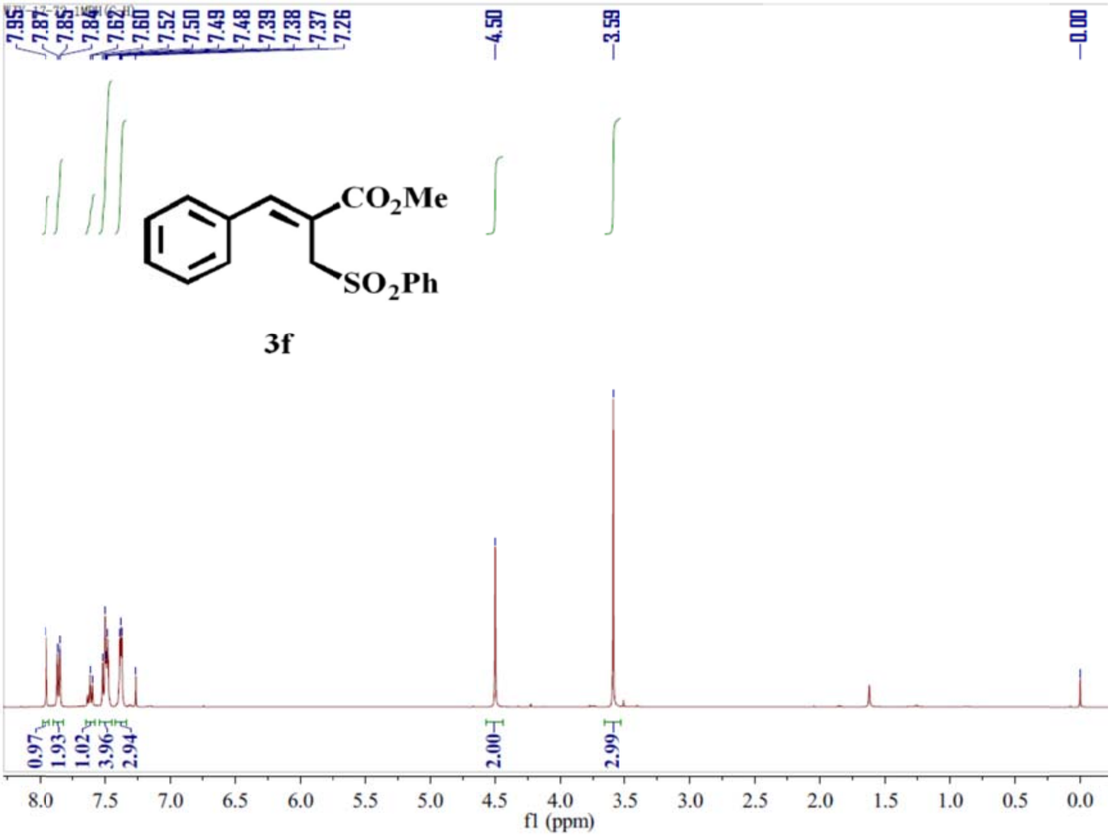

Supplementary Figure 13 | ^{13}C NMR (101 MHz, $CDCl_3$) spectra for compound **3f**.

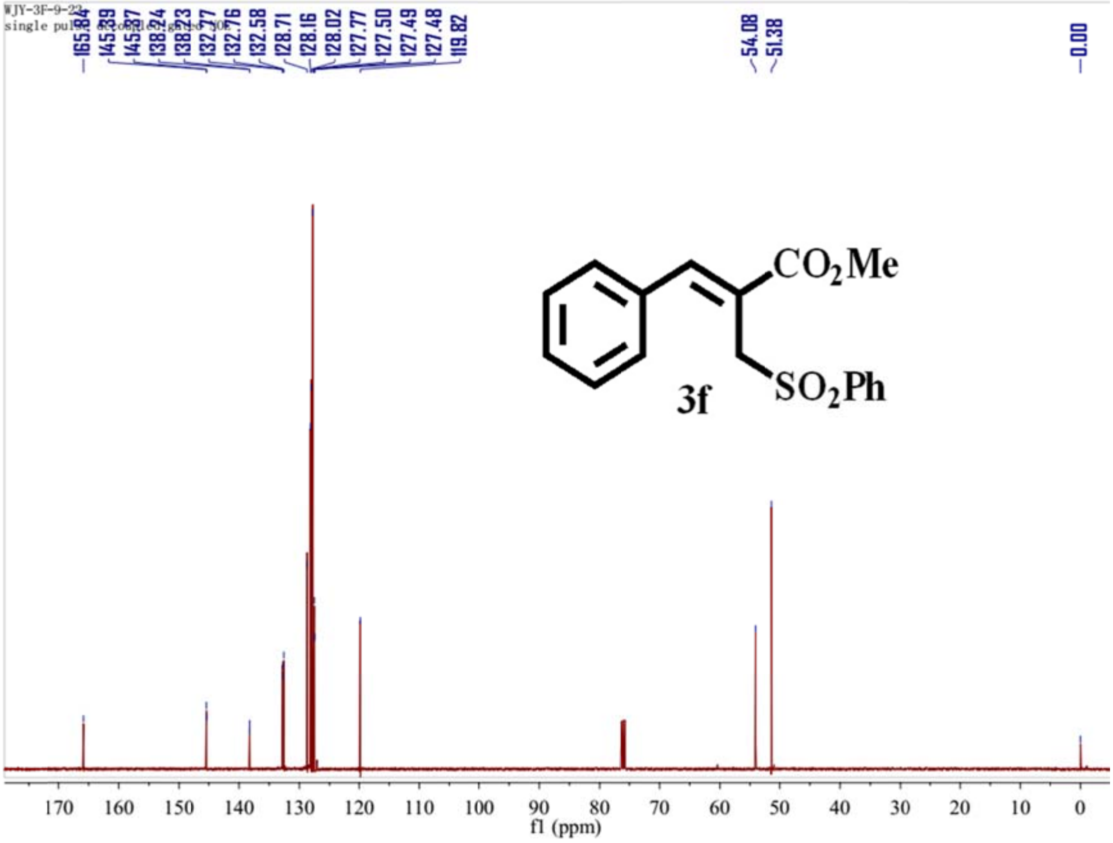

Supplementary Figure 14 | ^{1}H NMR (400 MHz, $CDCl_3$) spectra for compound **3g**.

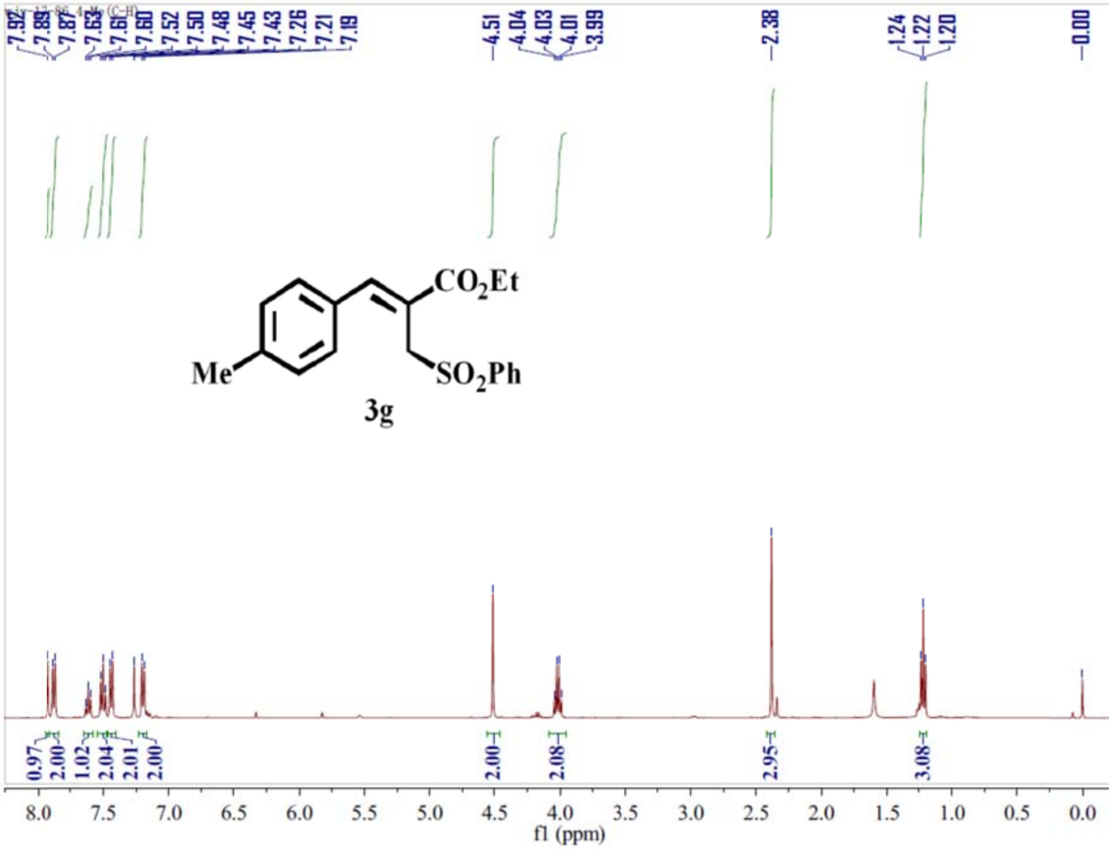

Supplementary Figure 15 | ^{13}C NMR (101 MHz, $CDCl_3$) spectra for compound **3g**.

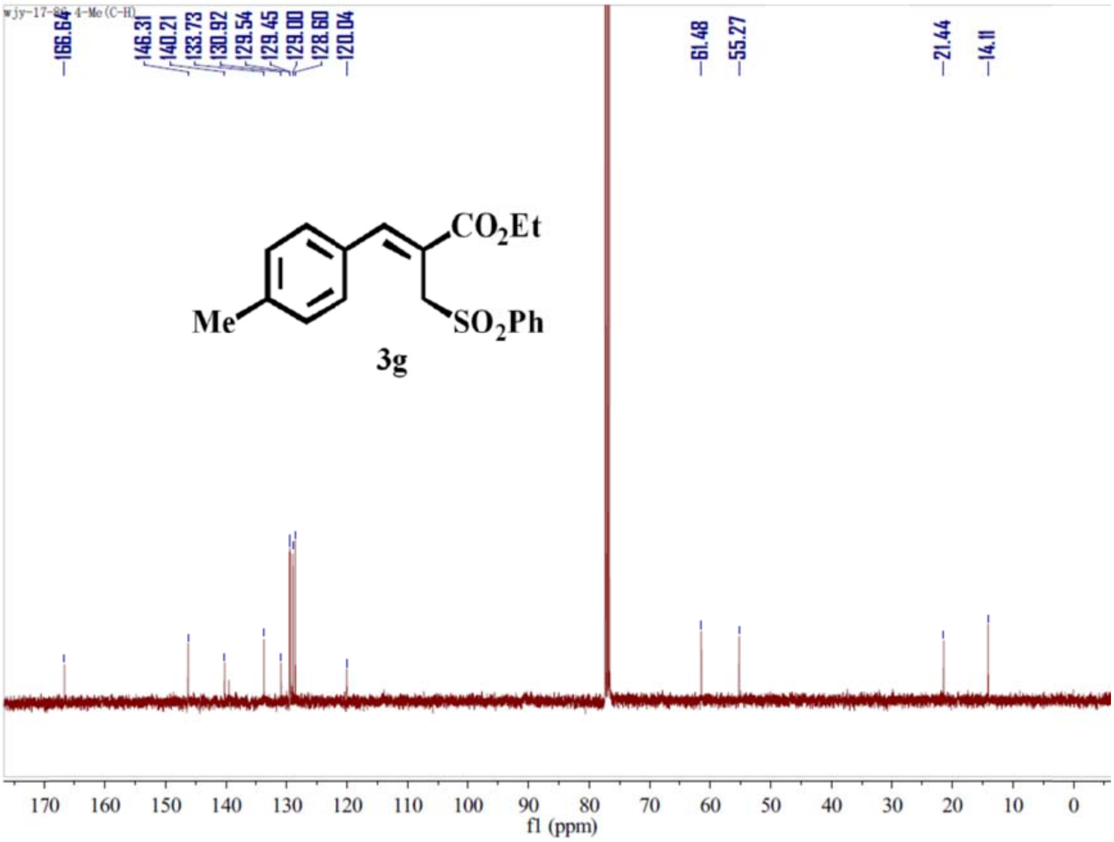

Supplementary Figure 16 | ^{1}H NMR (400 MHz, $CDCl_3$) spectra for compound **3h**.

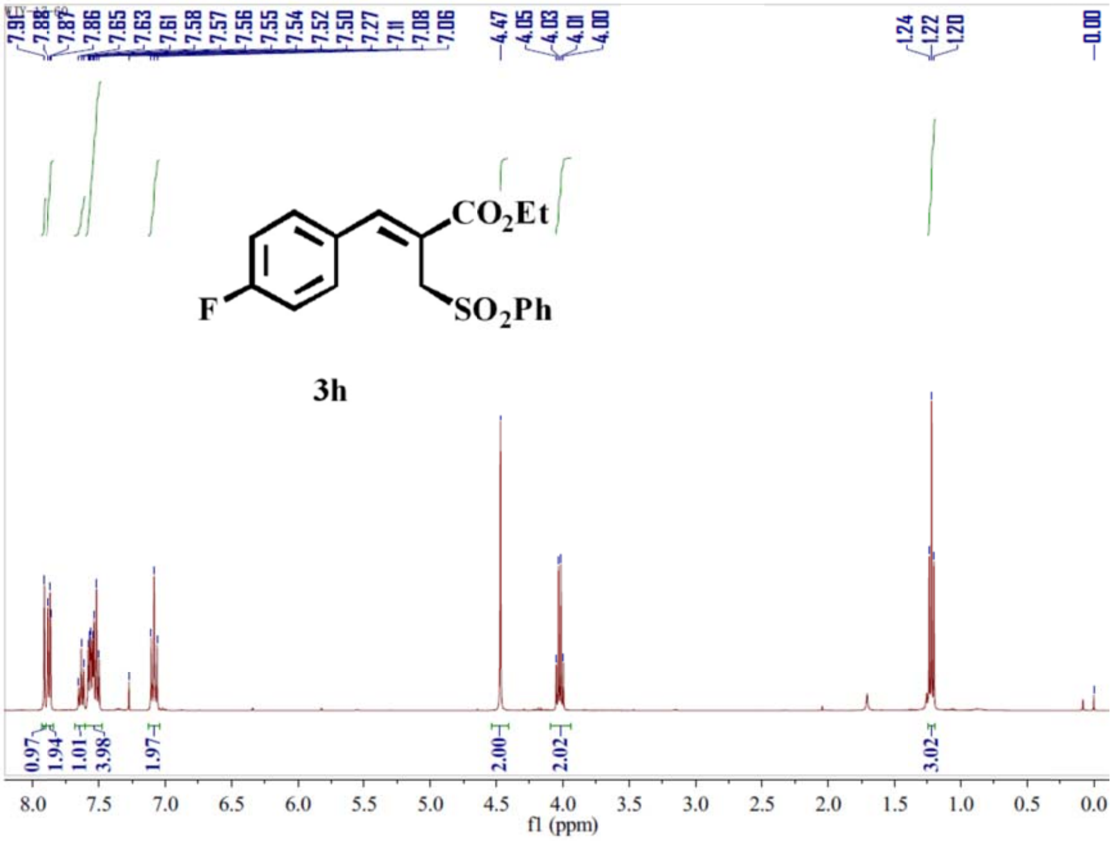

Supplementary Figure 17 | ^{13}C NMR (101 MHz, $CDCl_3$) spectra for compound **3h**.

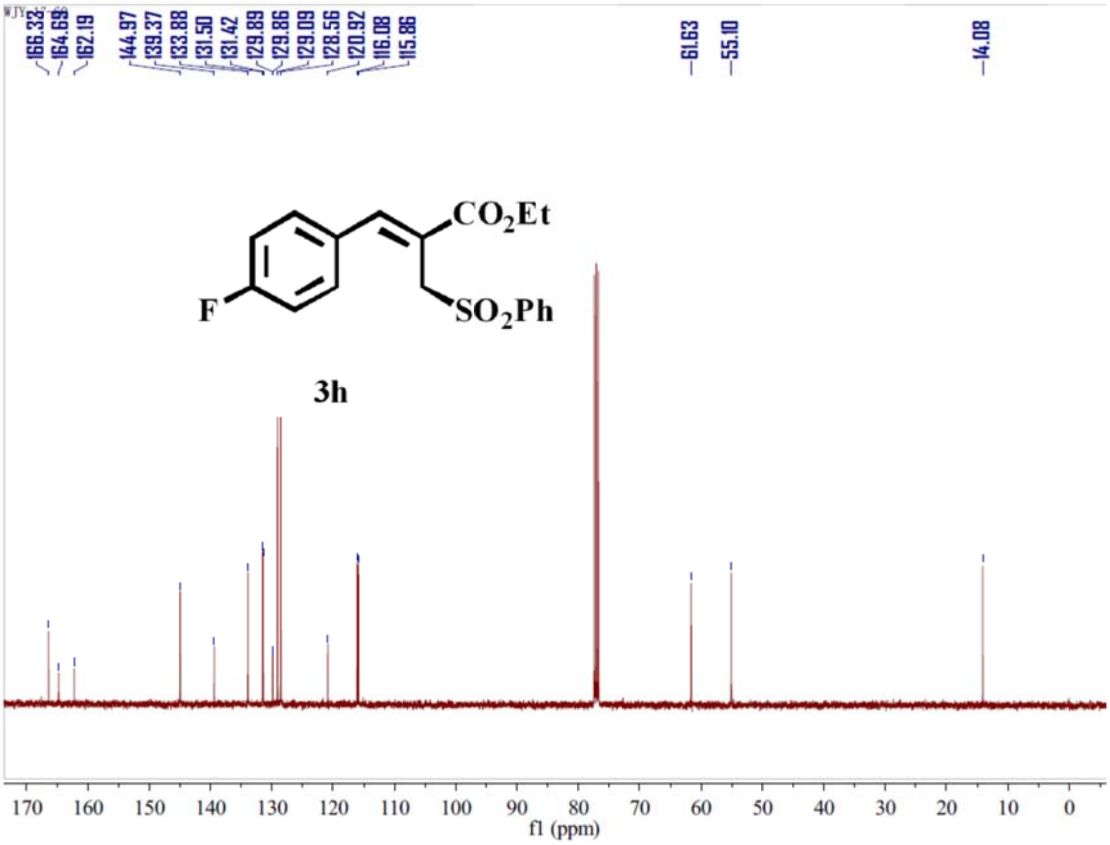

Supplementary Figure 18 | ^{1}H NMR (400 MHz, $CDCl_3$) spectra for compound **3i**.

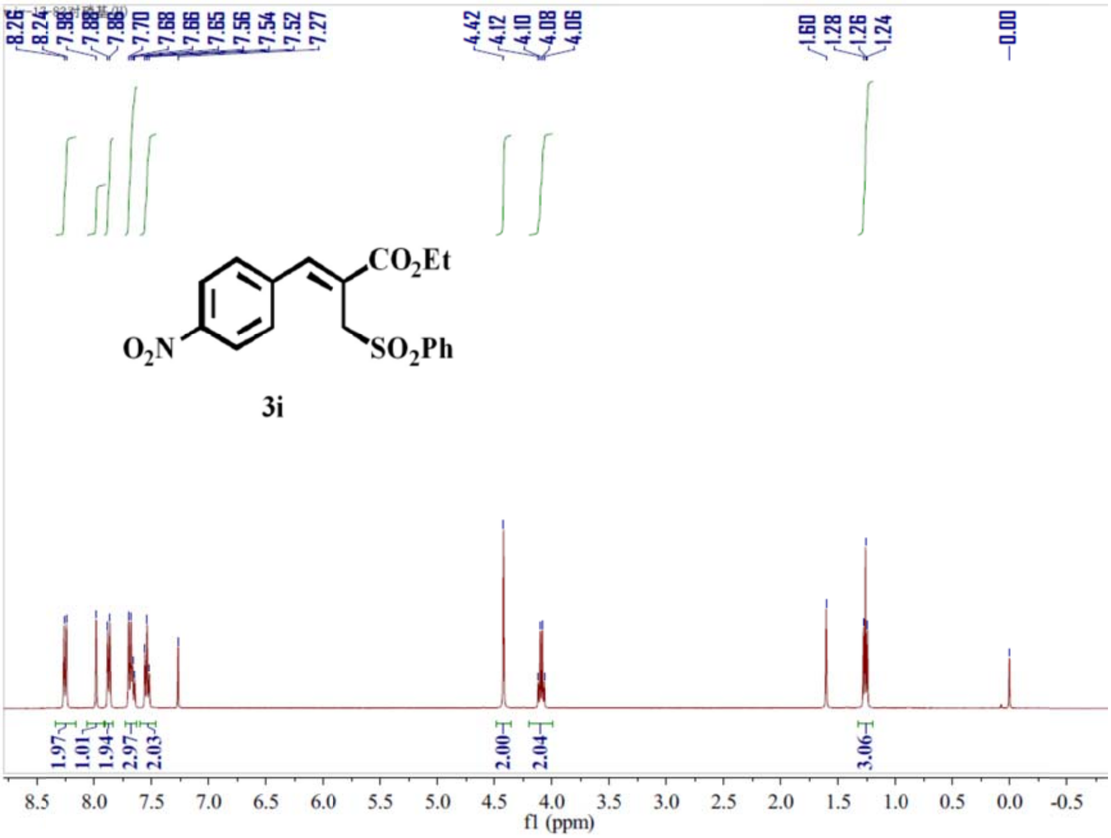

Supplementary Figure 19 | ^{13}C NMR (101 MHz, $CDCl_3$) spectra for compound **3i**.

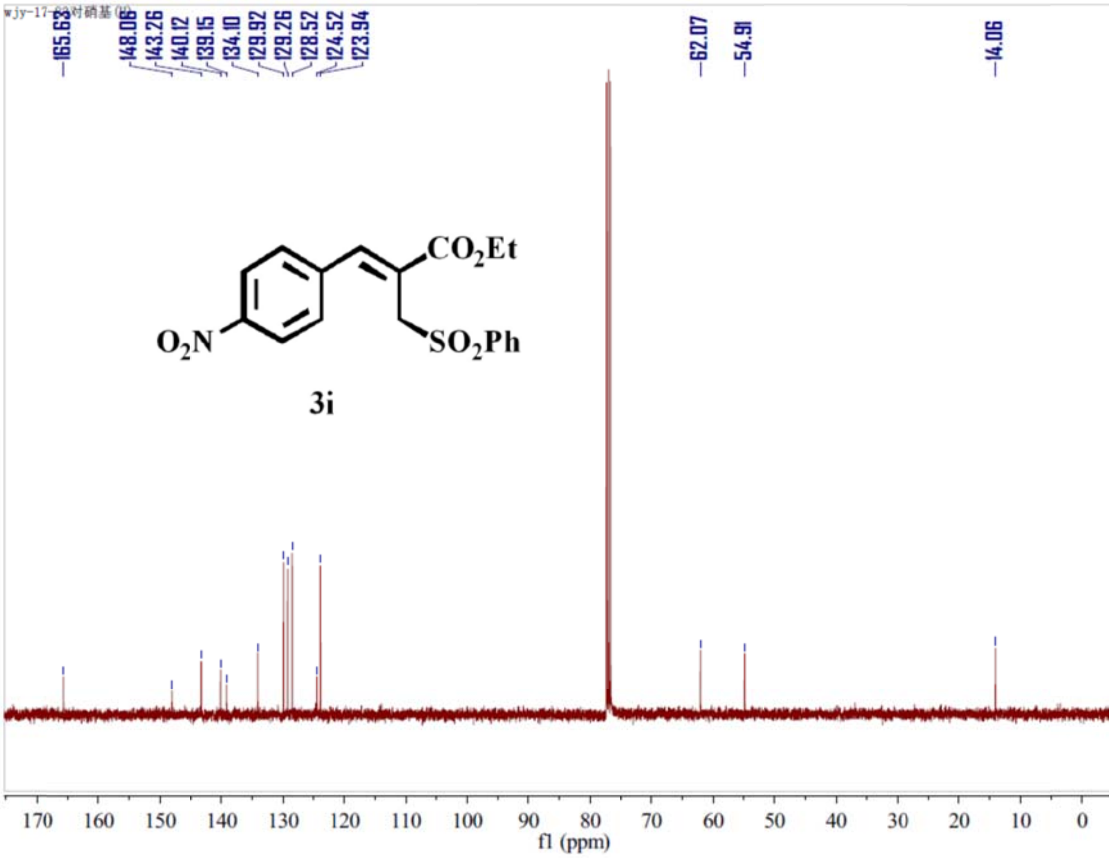

Supplementary Figure 20 | ^{1}H NMR (400 MHz, $CDCl_3$) spectra for compound **3j**.

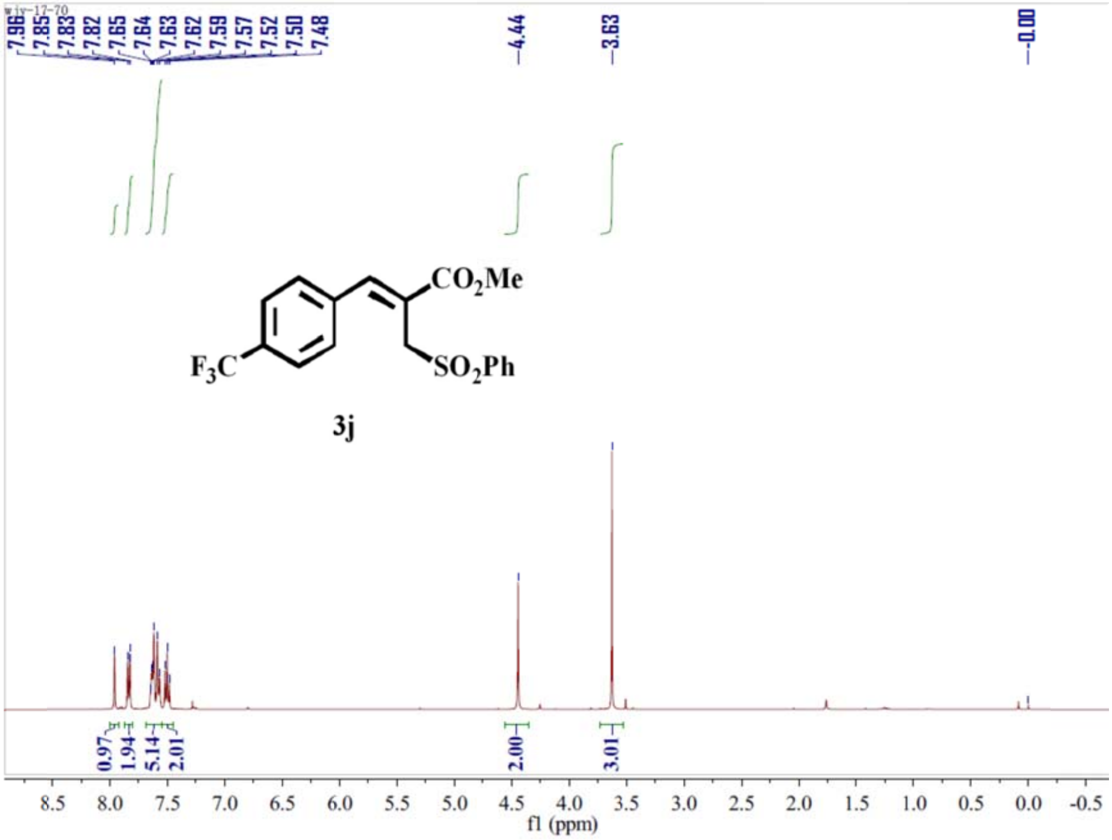

Supplementary Figure 21 | ^{13}C NMR (101 MHz, $CDCl_3$) spectra for compound **3j.**

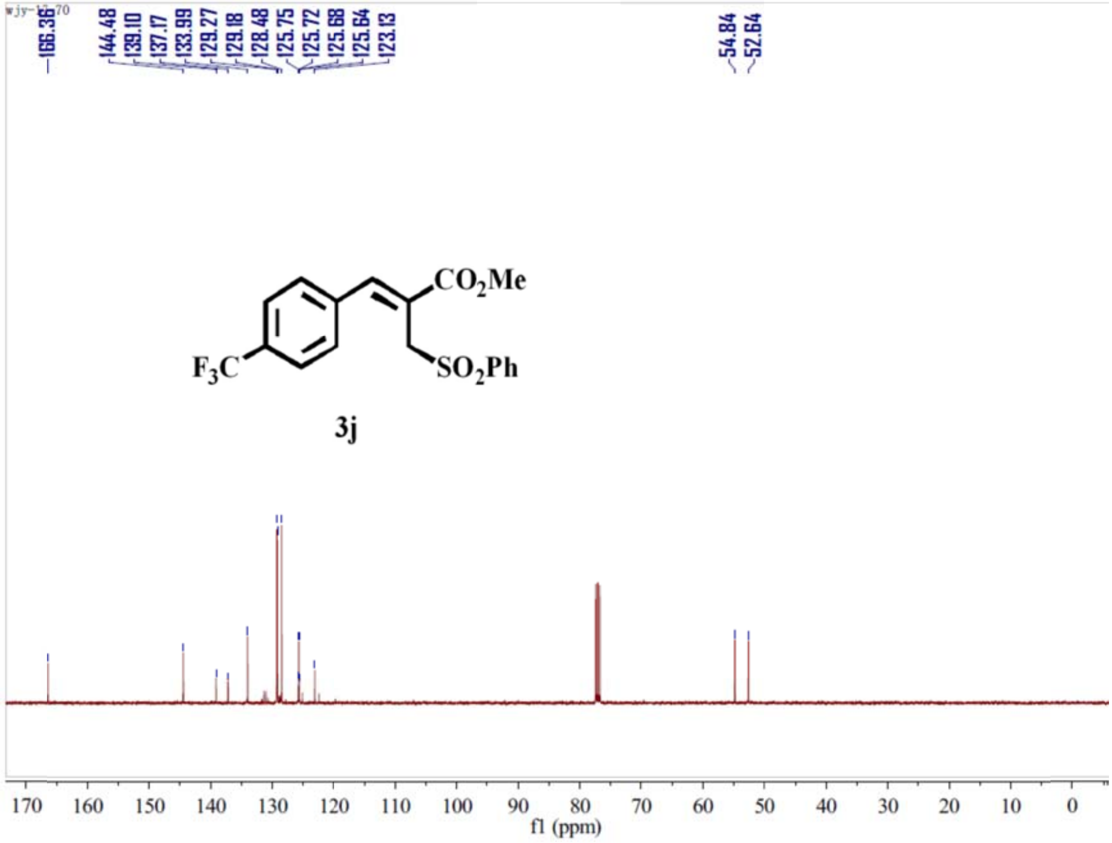

Supplementary Figure 22 | ^{1}H NMR (400 MHz, $CDCl_3$) spectra for compound **3k.**

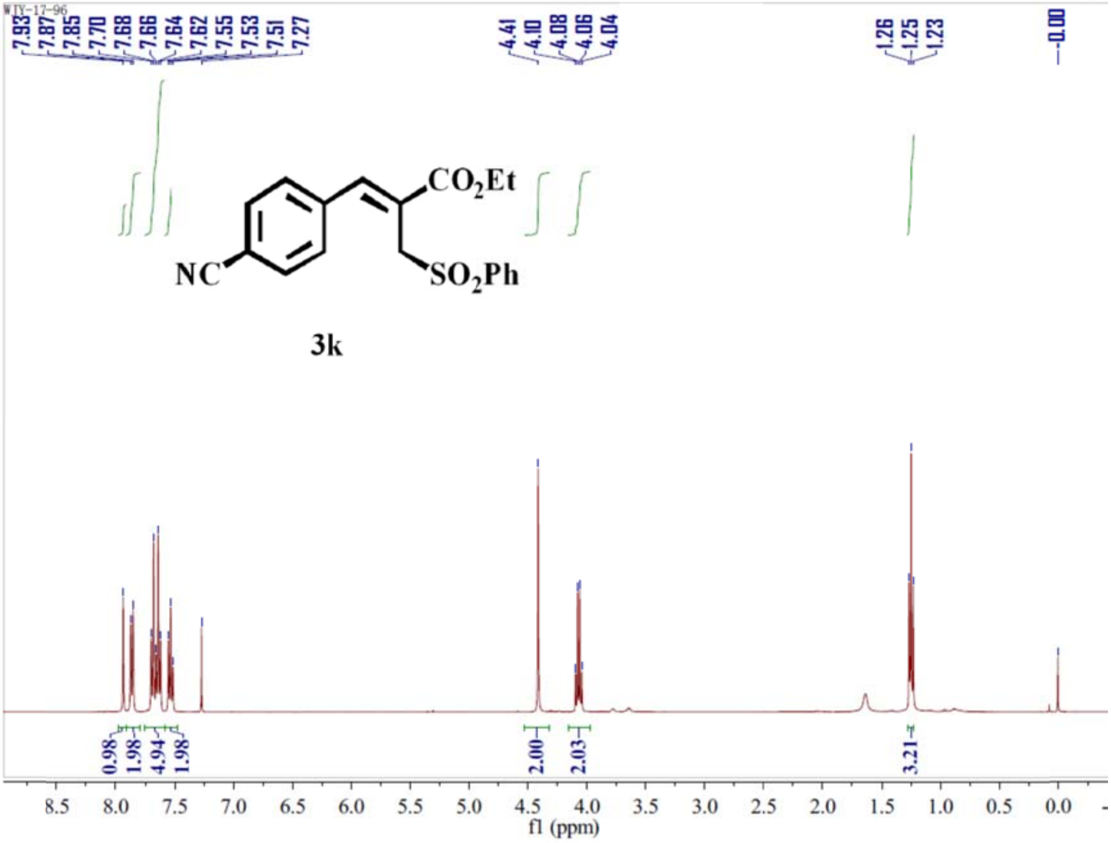

Supplementary Figure 23 | ^{13}C NMR (101 MHz, $CDCl_3$) spectra for compound **3k.**

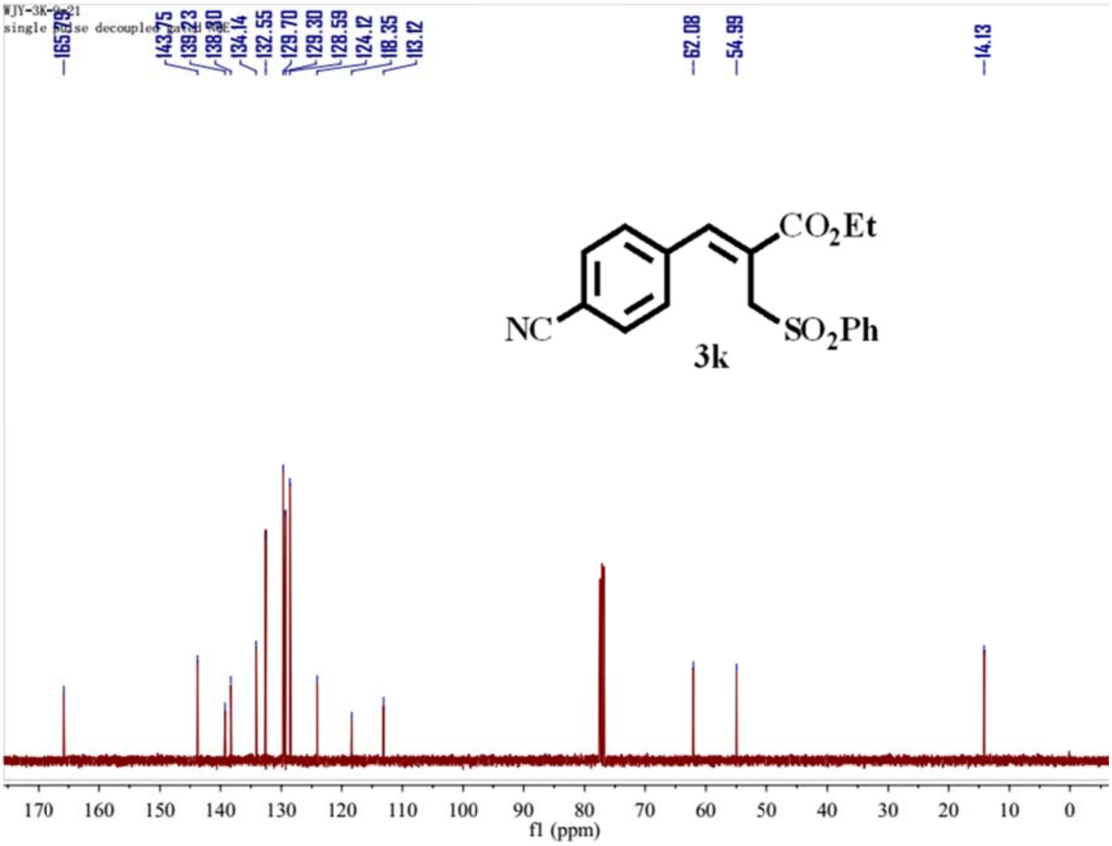

Supplementary Figure 24 | ^{1}H NMR (400 MHz, $CDCl_3$) spectra for compound **3l.**

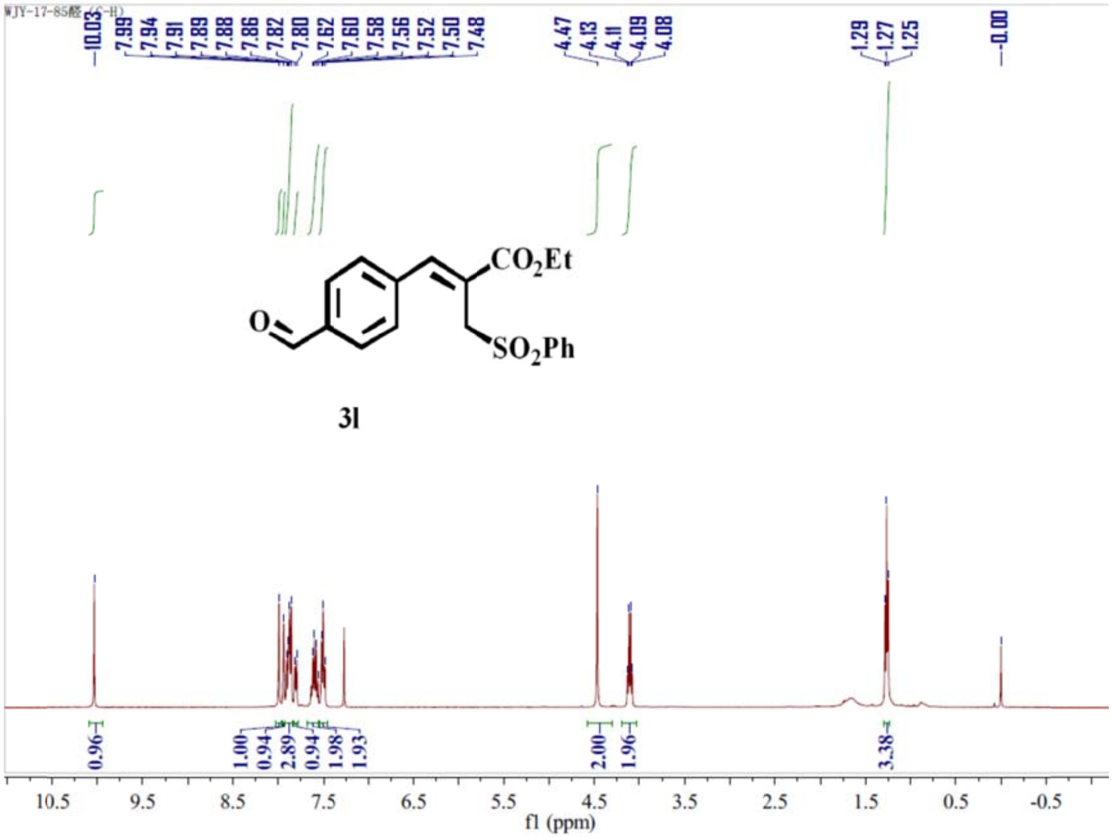

Supplementary Figure 25 | ^{13}C NMR (101 MHz, $CDCl_3$) spectra for compound **3l.**

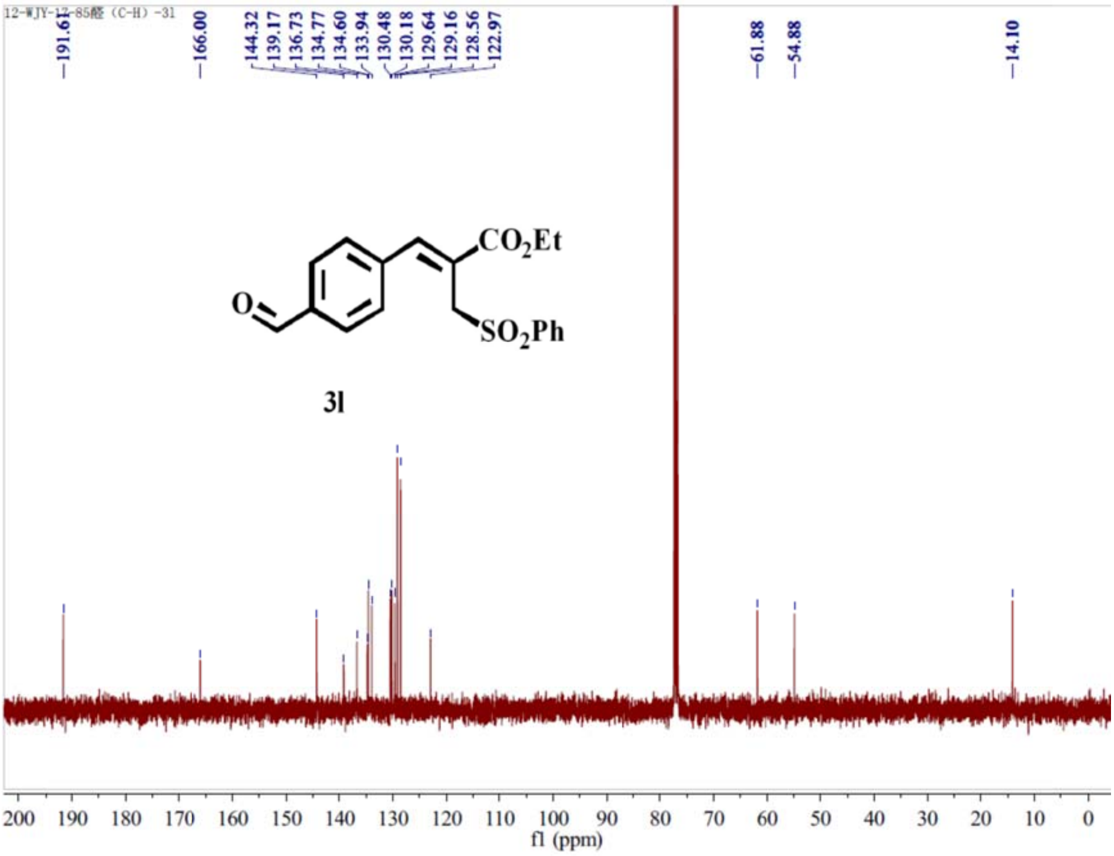

Supplementary Figure 26 | ^{1}H NMR (400 MHz, $CDCl_3$) spectra for compound **3m.**

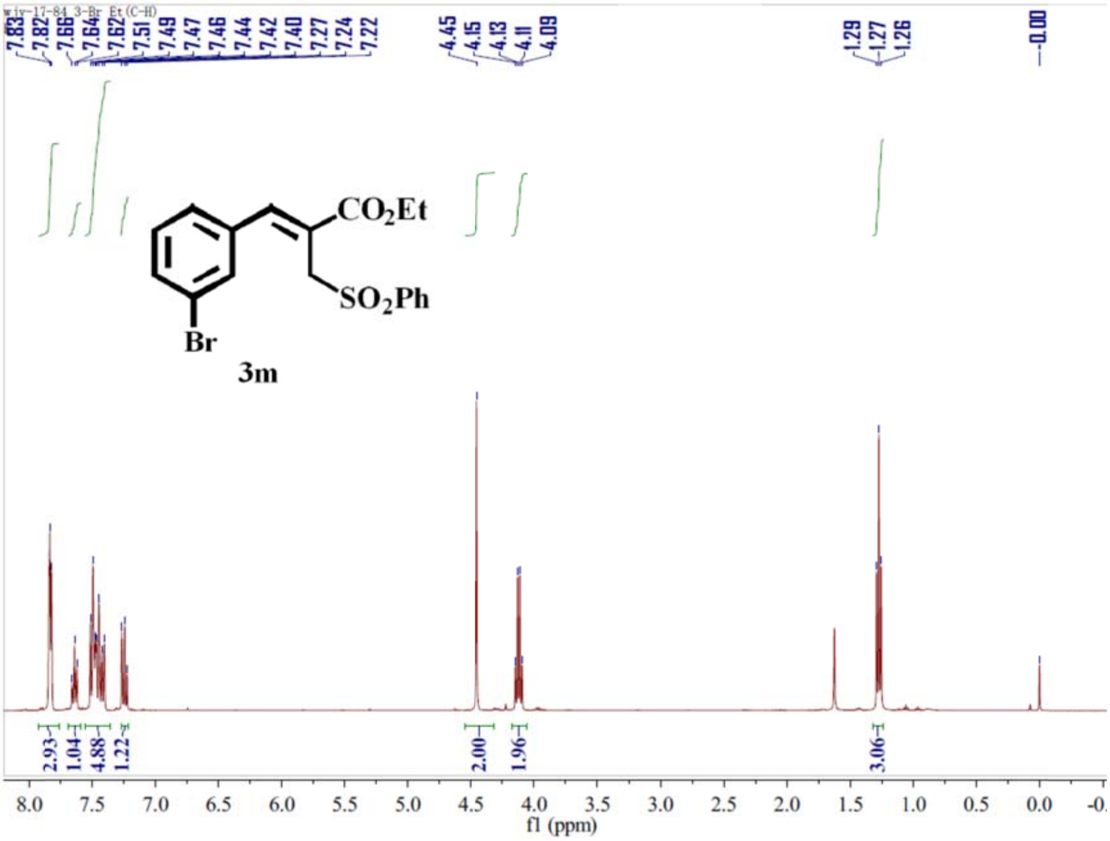

Supplementary Figure 27 | ^{13}C NMR (101 MHz, $CDCl_3$) spectra for compound **3m.**

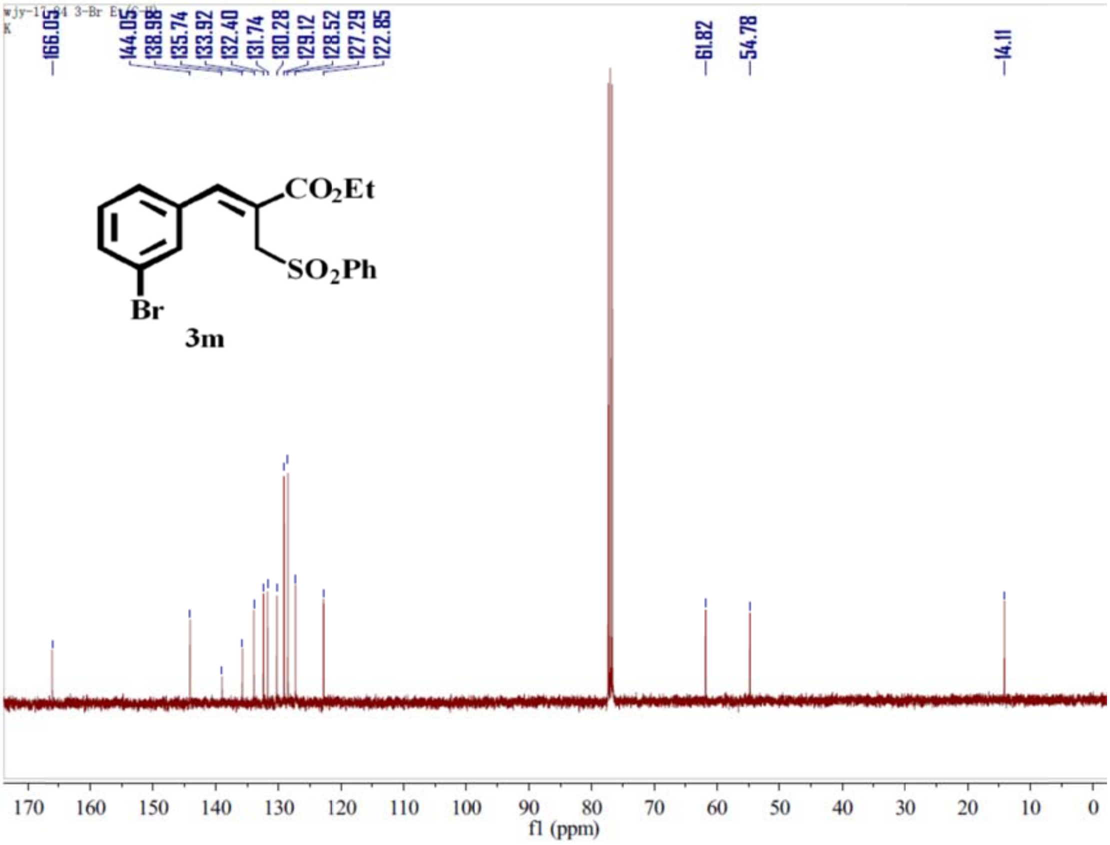

Supplementary Figure 28 | ^{1}H NMR (400 MHz, $CDCl_3$) spectra for compound **3n.**

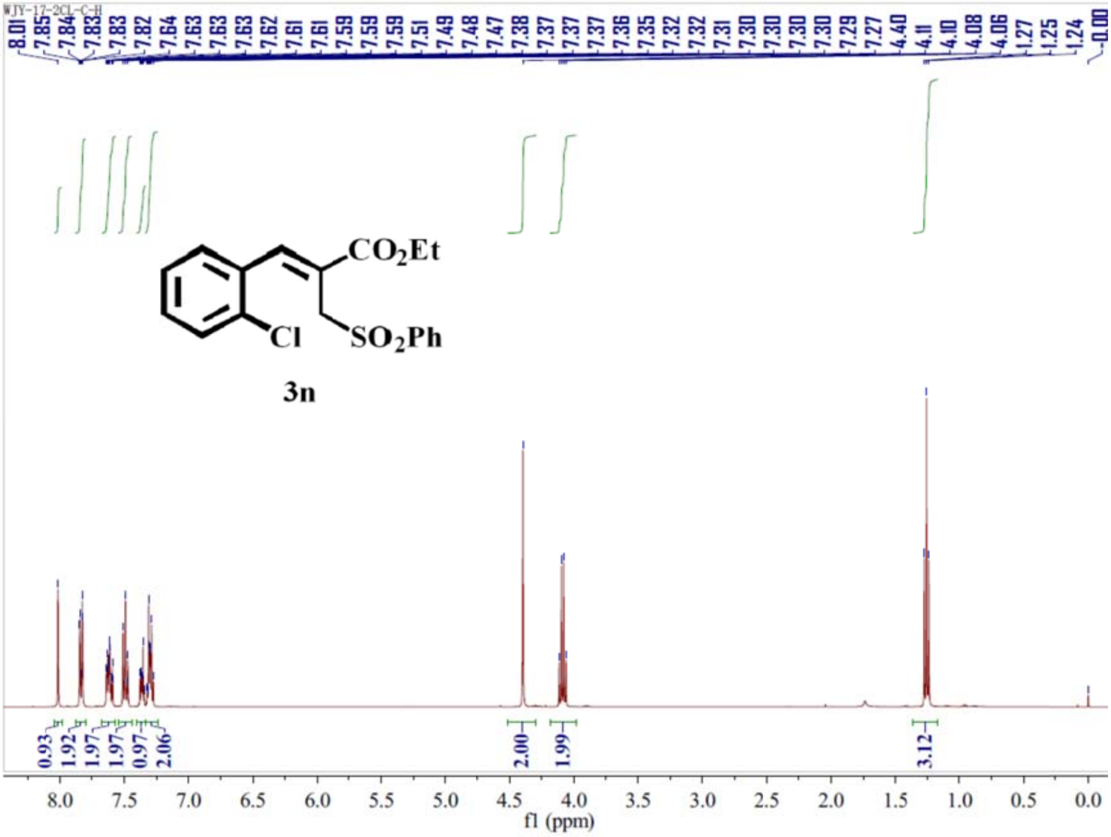

Supplementary Figure 29 | ^{13}C NMR (101 MHz, $CDCl_3$) spectra for compound **3n.**

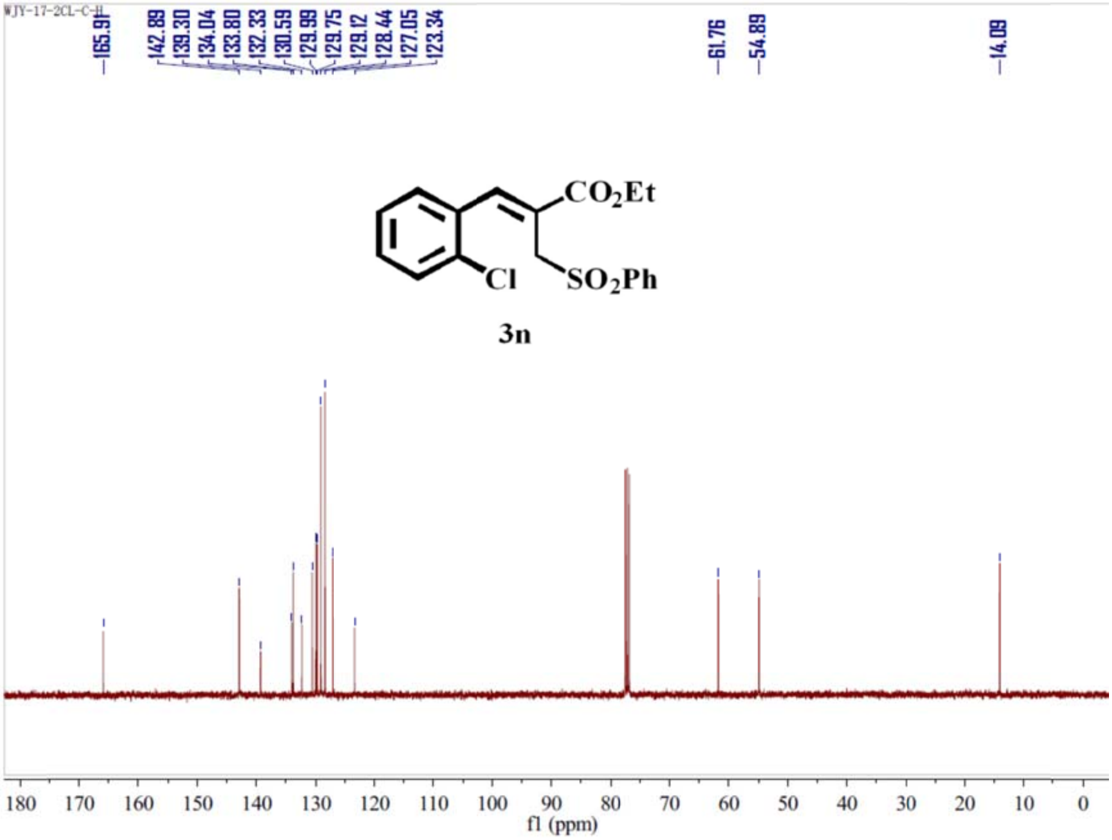

Supplementary Figure 30 | ^{1}H NMR (400 MHz, $CDCl_3$) spectra for compound **3o.**

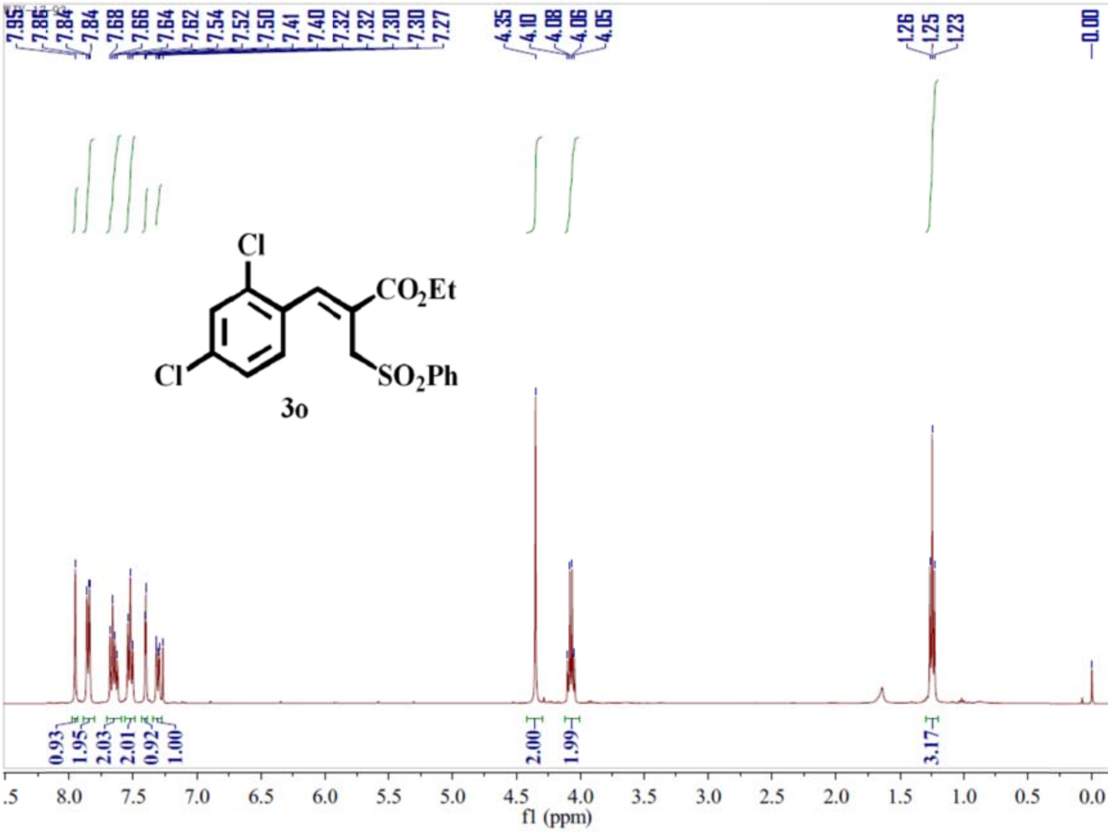

Supplementary Figure 31 | ^{13}C NMR (101 MHz, $CDCl_3$) spectra for compound **3o.**

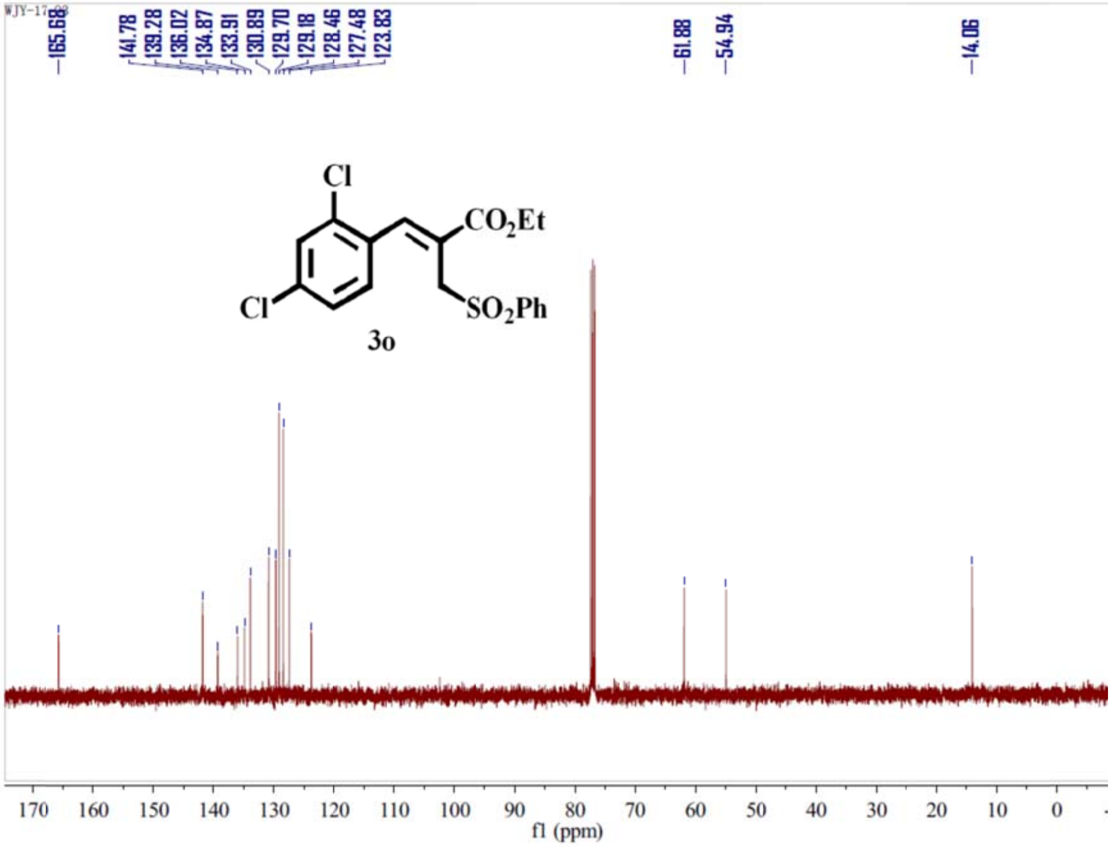

Supplementary Figure 32 | ^{1}H NMR (400 MHz, $CDCl_3$) spectra for compound **3p.**

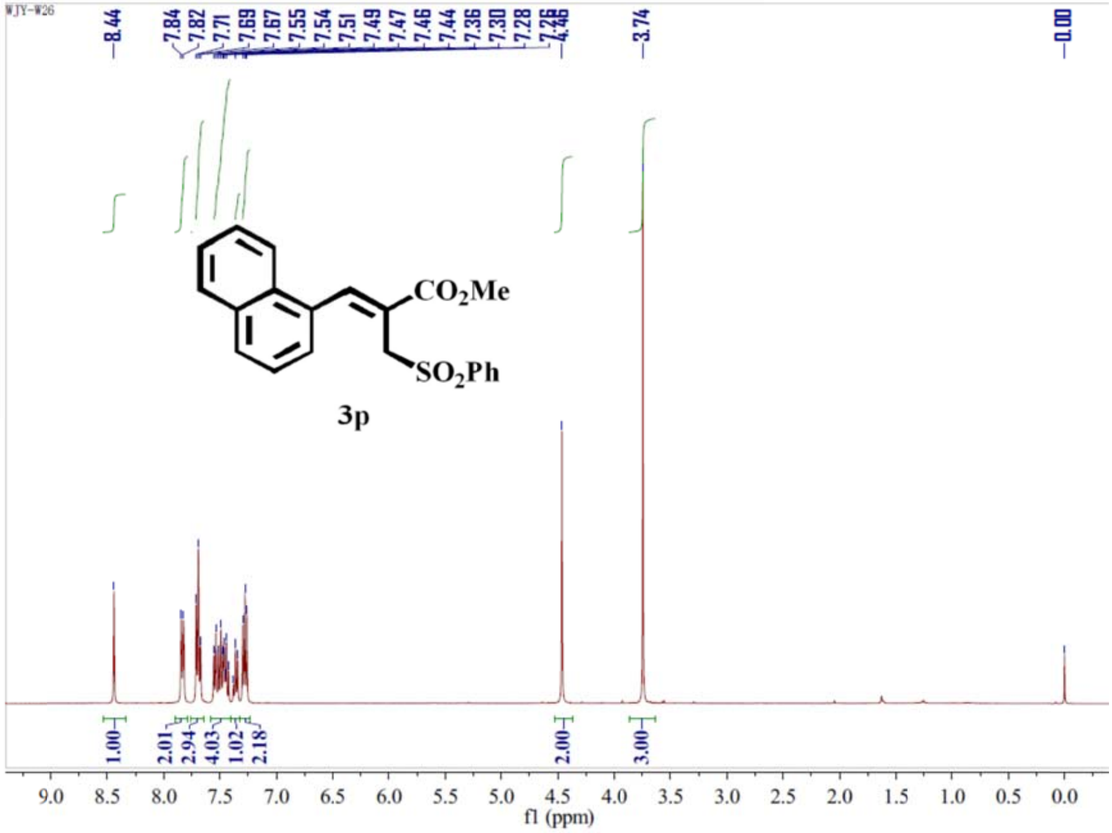

Supplementary Figure 33 | ^{13}C NMR (101 MHz, $CDCl_3$) spectra for compound **3p.**

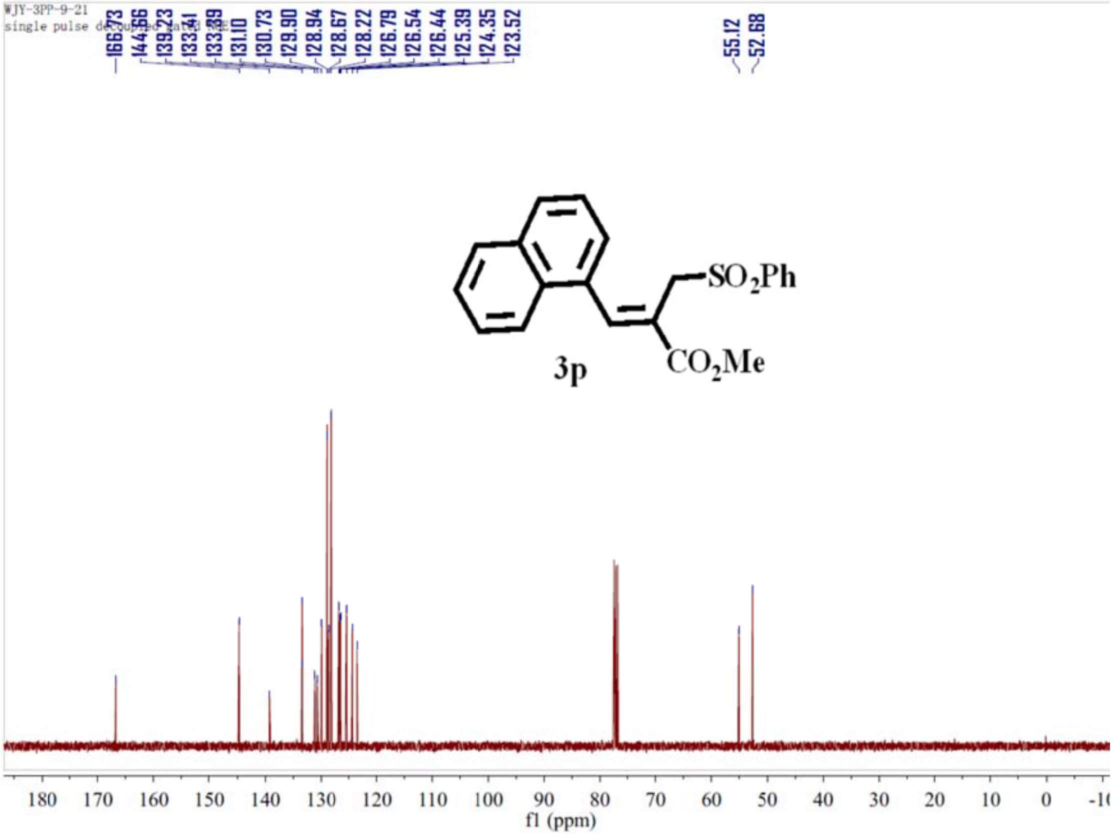

Supplementary Figure 34 | ^{1}H NMR (400 MHz, $CDCl_3$) spectra for compound **3q.**

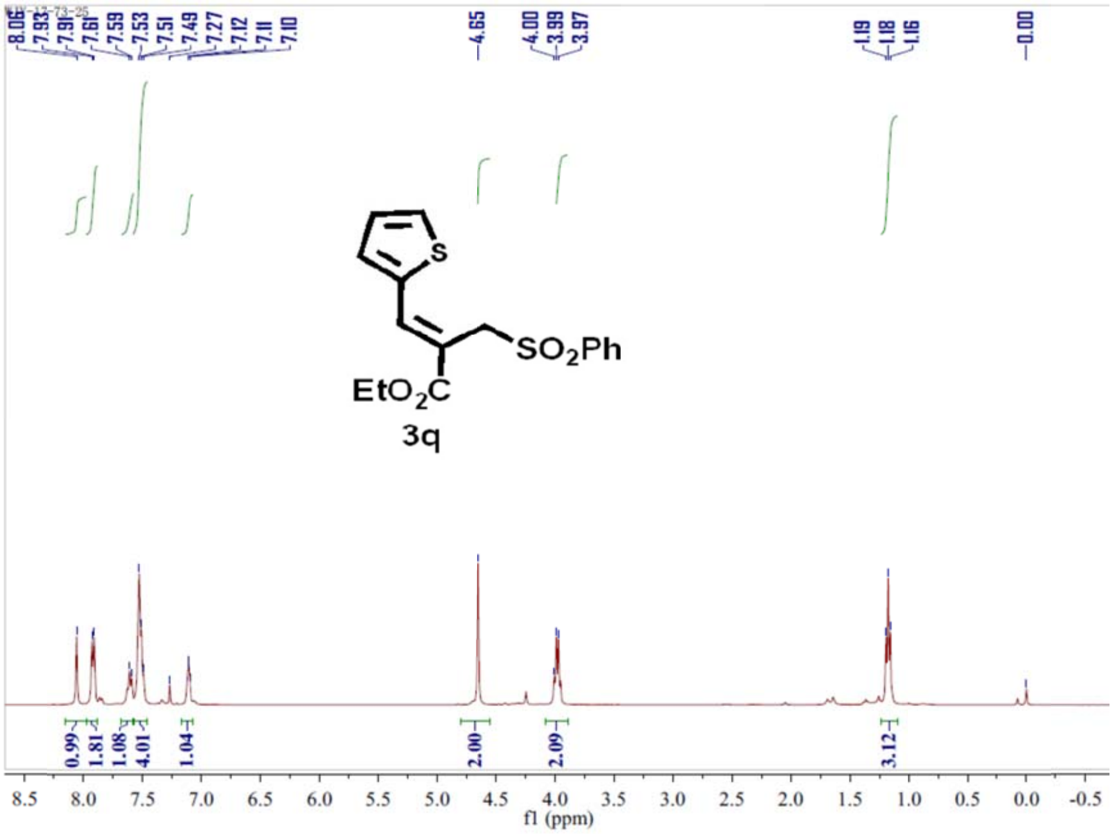

Supplementary Figure 35 | ^{13}C NMR (101 MHz, $CDCl_3$) spectra for compound **3q.**

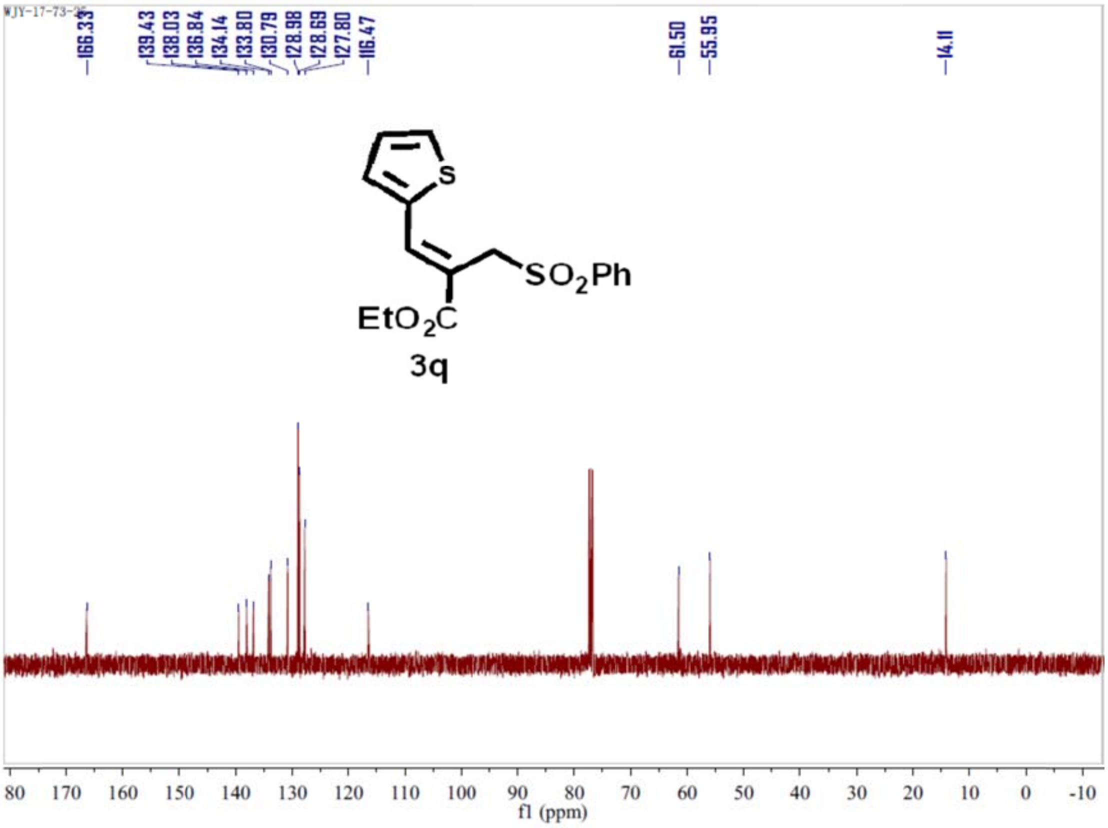

Supplementary Figure 36 | ^{1}H NMR (400 MHz, $CDCl_3$) spectra for compound **3r.**

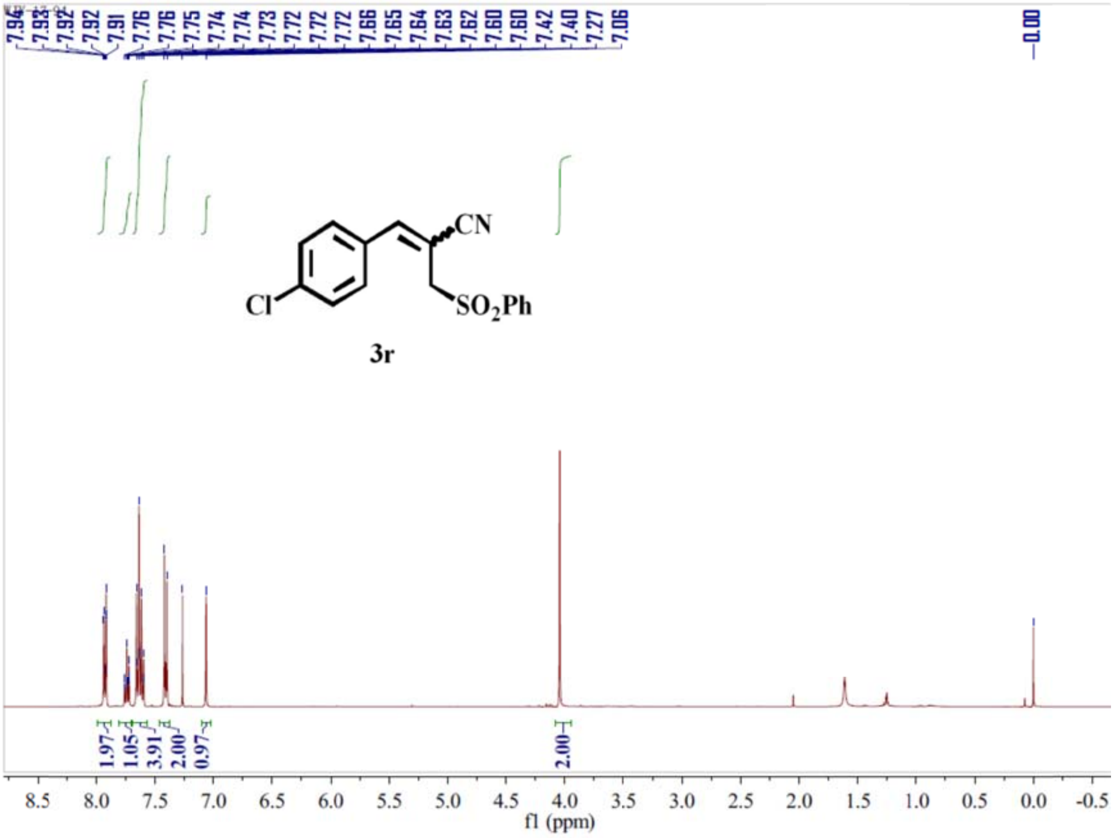

Supplementary Figure 37 | ^{13}C NMR (101 MHz, $CDCl_3$) spectra for compound **3r.**

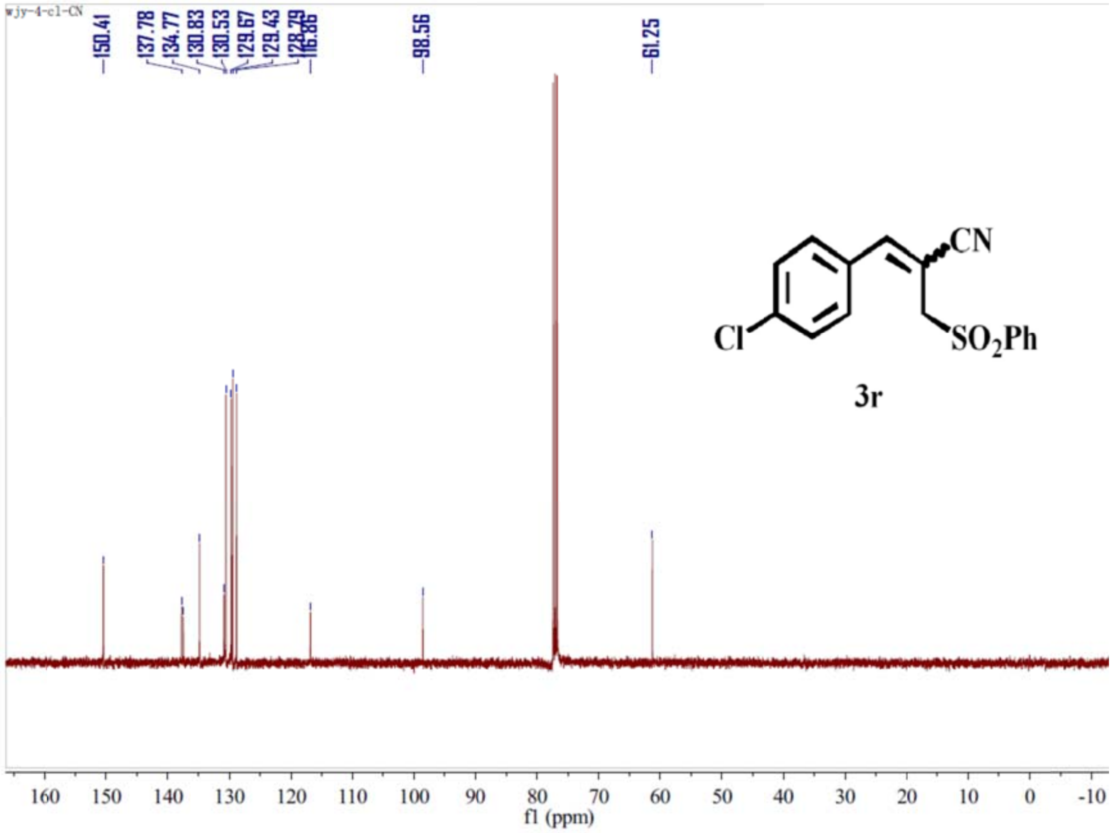

Supplementary Figure 38 | ^{1}H NMR (400 MHz, $CDCl_3$) spectra for compound **3s.**

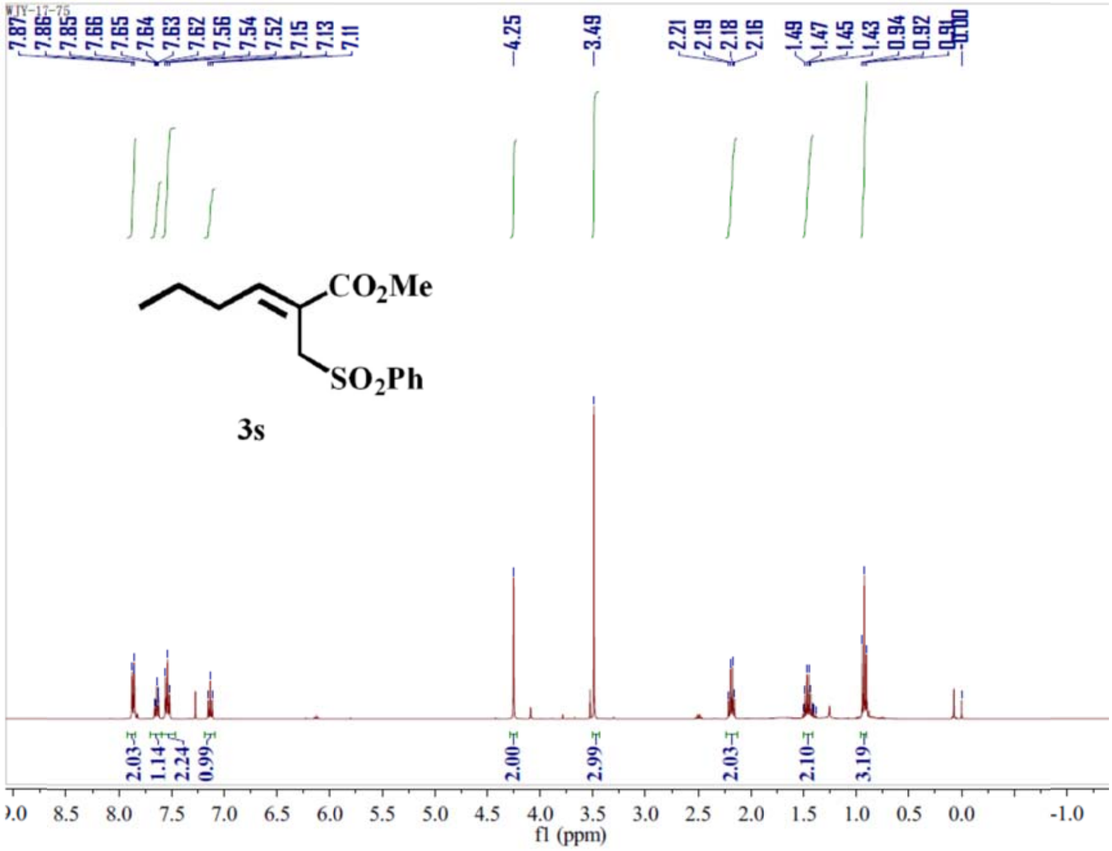

Supplementary Figure 39 | ^{13}C NMR (101 MHz, $CDCl_3$) spectra for compound **3s.**

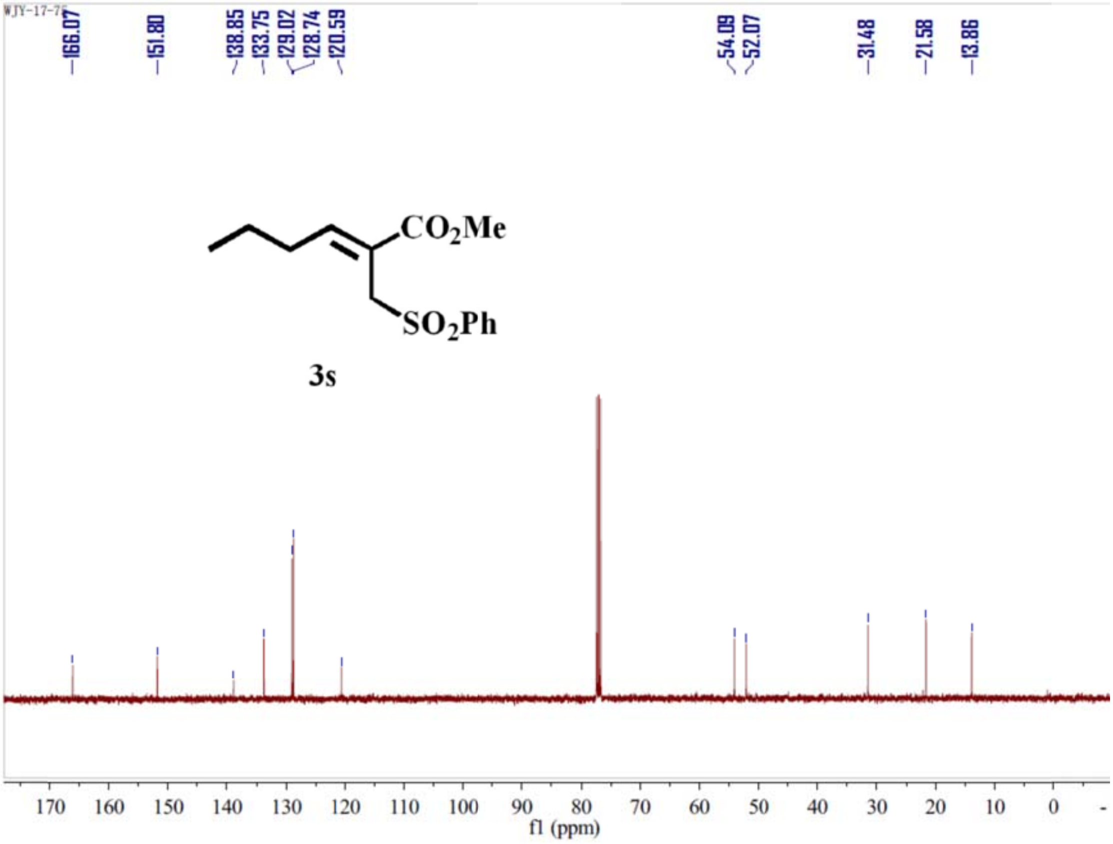

Supplementary Figure 40 | ^{1}H NMR (400 MHz, $CDCl_3$) spectra for compound **3t.**

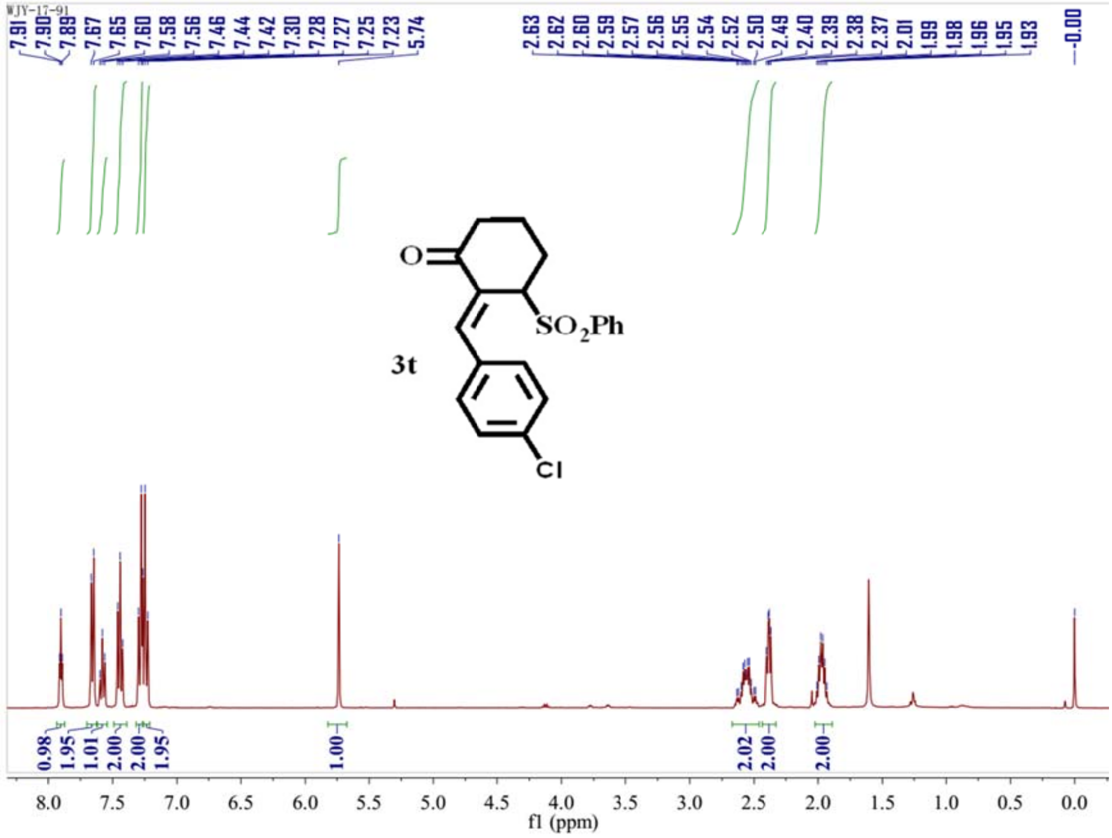

Supplementary Figure 41 | ^{13}C NMR (101 MHz, $CDCl_3$) spectra for compound **3t.**

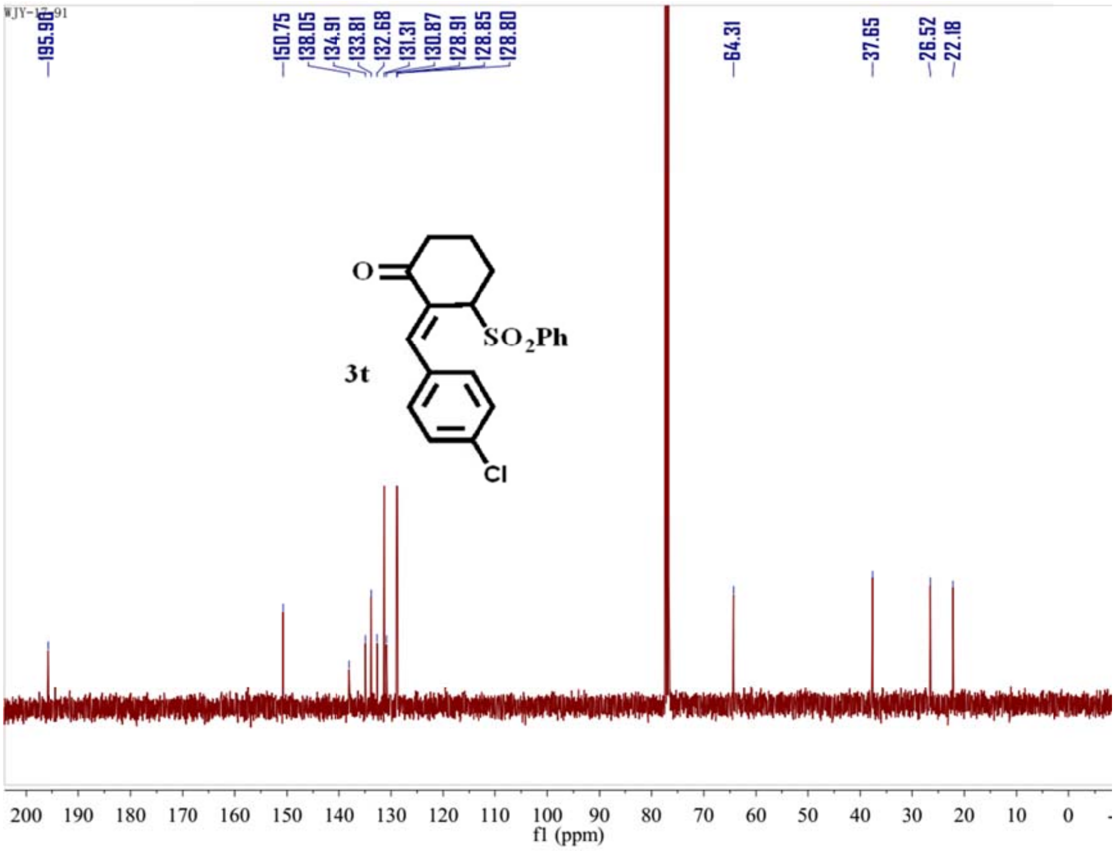

Supplementary Figure 42 | ^1H NMR (400 MHz, $CDCl_3$) spectra for compound **3u.**

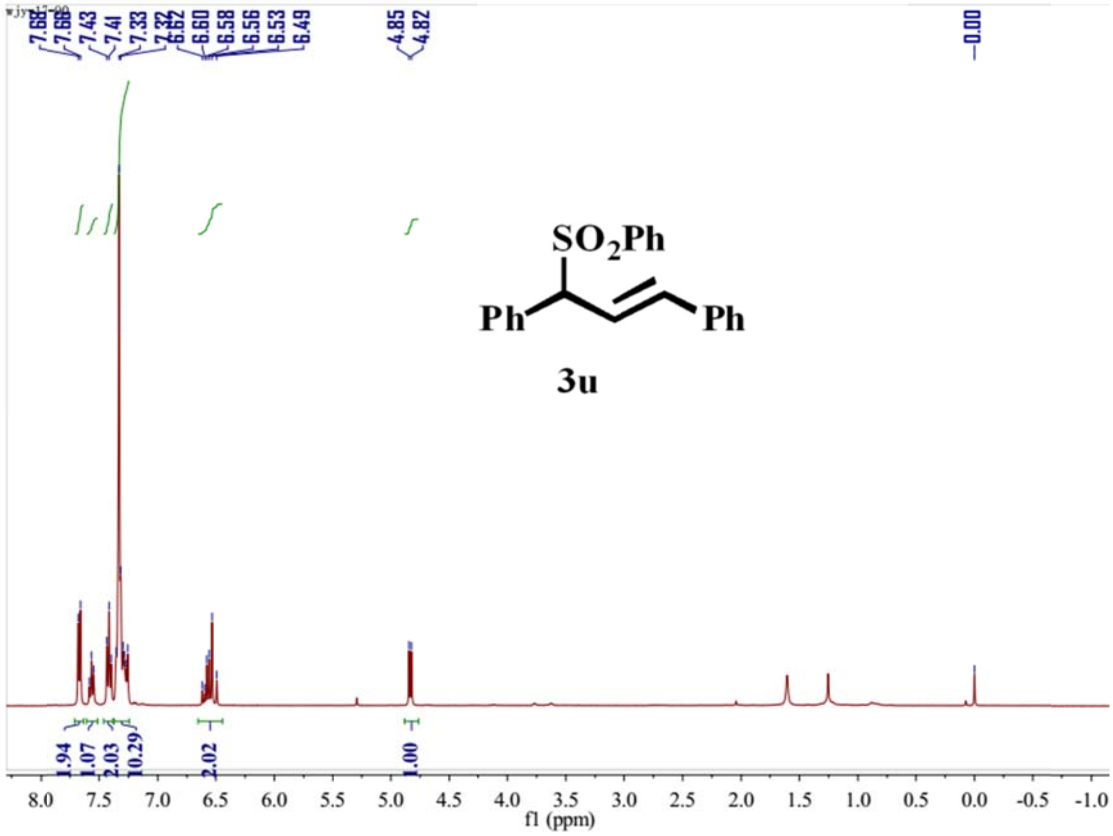

Supplementary Figure 43 | ^{13}C NMR (101 MHz, $CDCl_3$) spectra for compound **3u.**

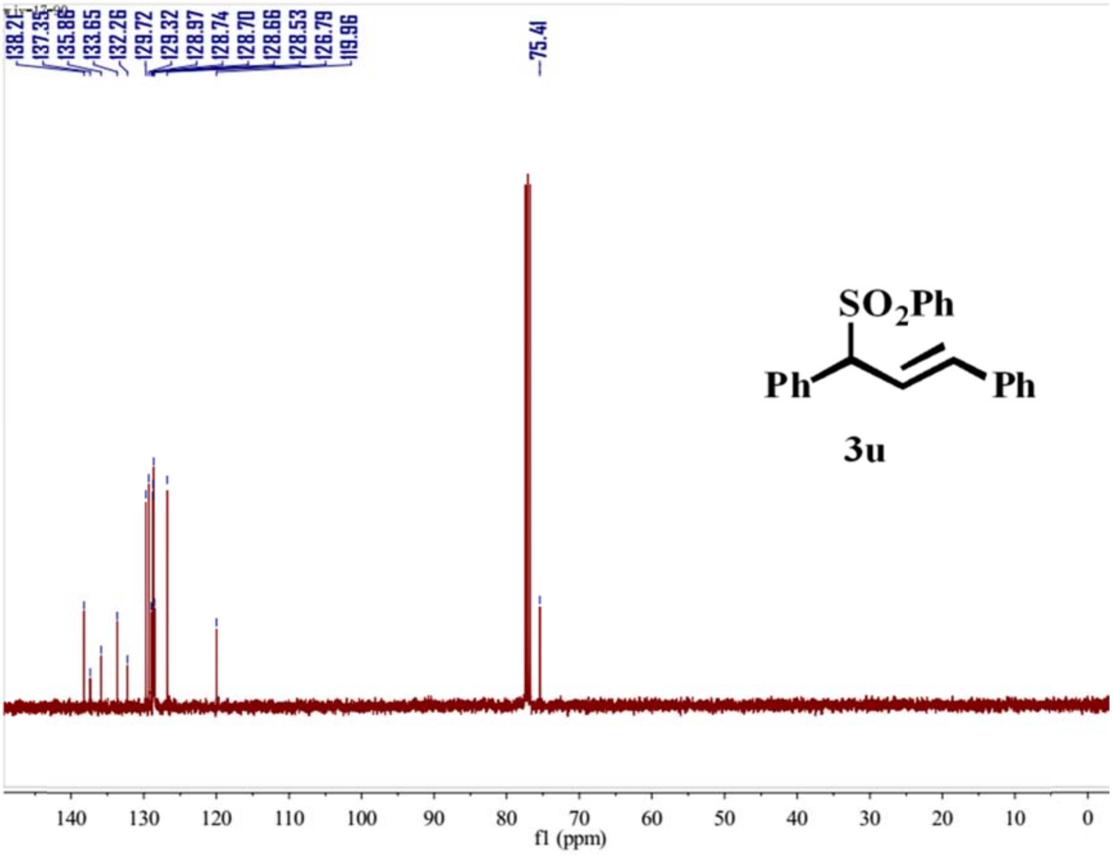

Supplementary Figure 44 | ^{1}H NMR (400 MHz, $CDCl_3$) spectra for compound **3v.**

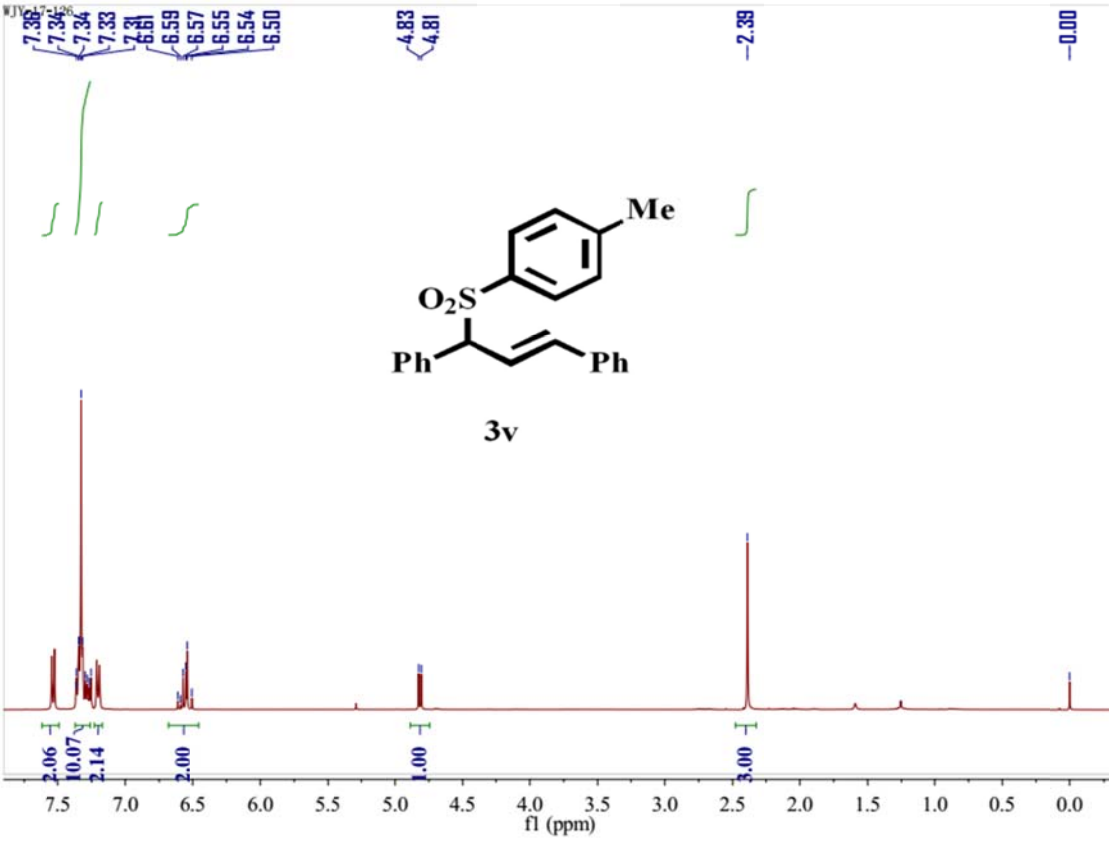

Supplementary Figure 45 | ^{13}C NMR (101 MHz, $CDCl_3$) spectra for compound **3v.**

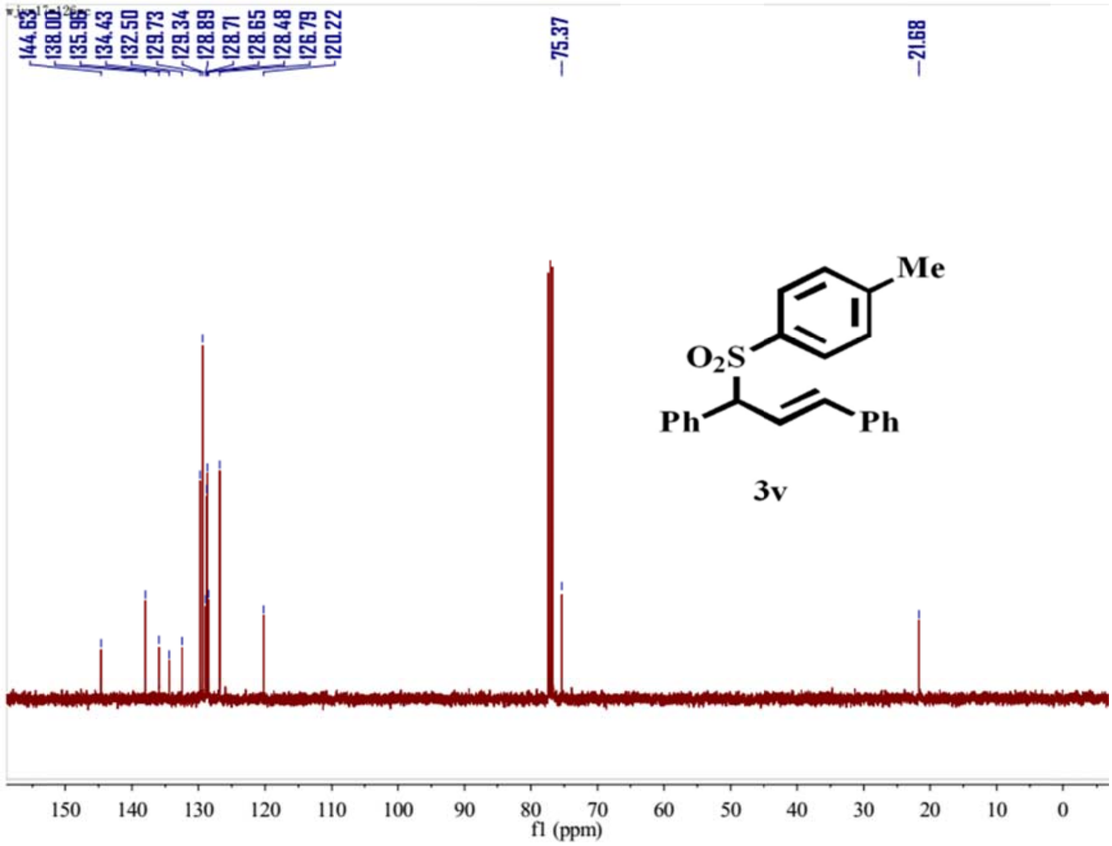

Supplementary Figure 46 | ^{1}H NMR (400 MHz, $CDCl_3$) spectra for compound **3w.**

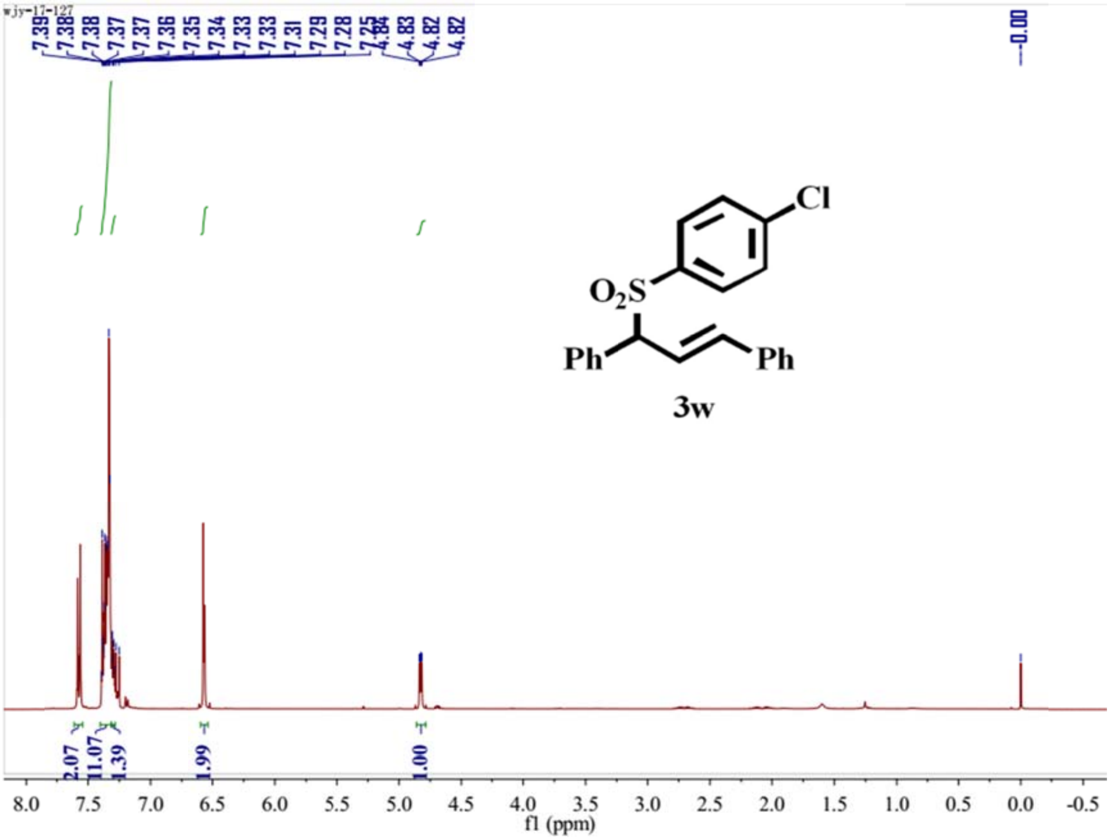

Supplementary Figure 47 | ^{13}C NMR (101 MHz, $CDCl_3$) spectra for compound **3w.**

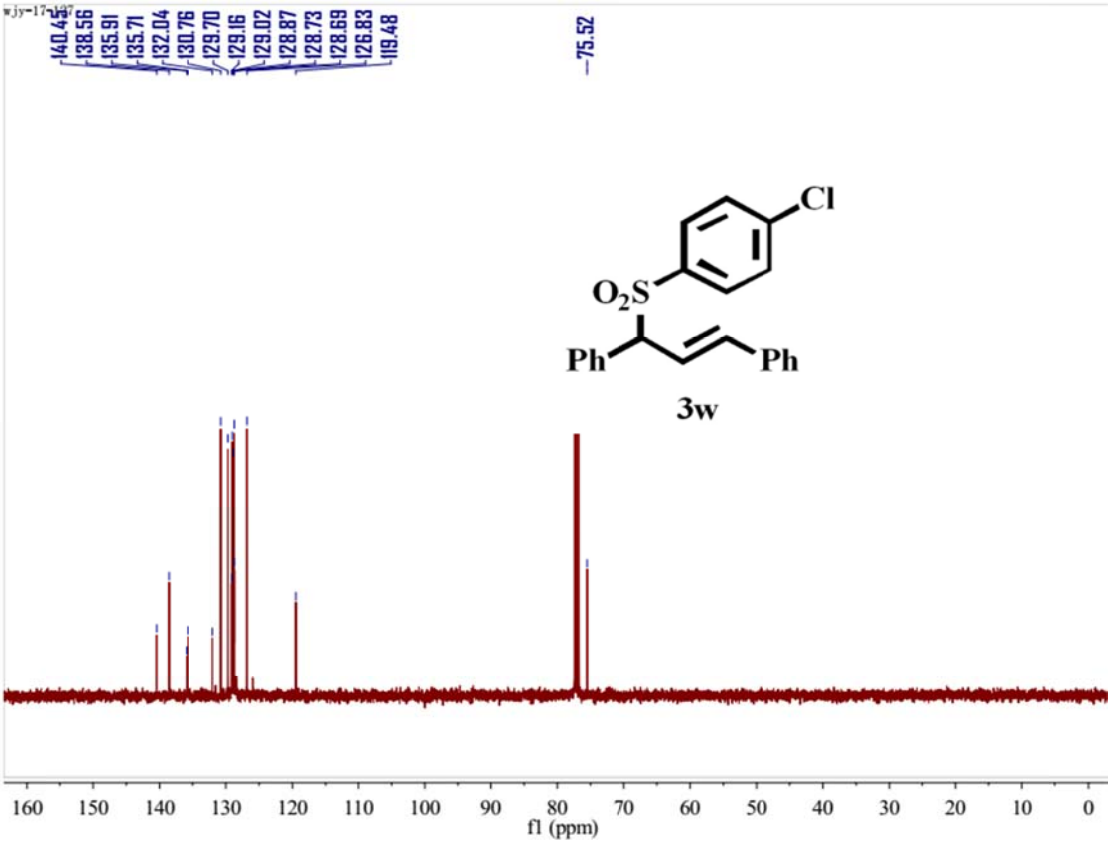

Supplementary Figure 48 | ^{1}H NMR (400 MHz, $CDCl_3$) spectra for compound **3x.**

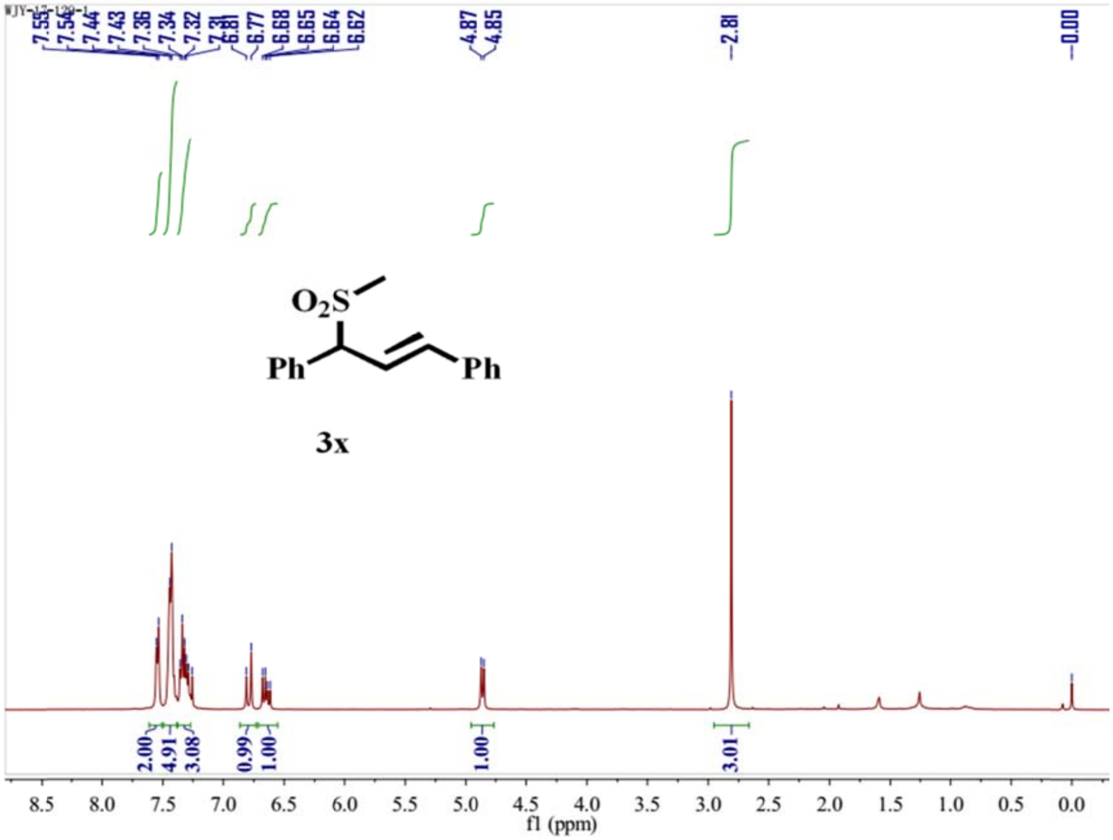

Supplementary Figure 49 | ^{13}C NMR (101 MHz, $CDCl_3$) spectra for compound **3x.**

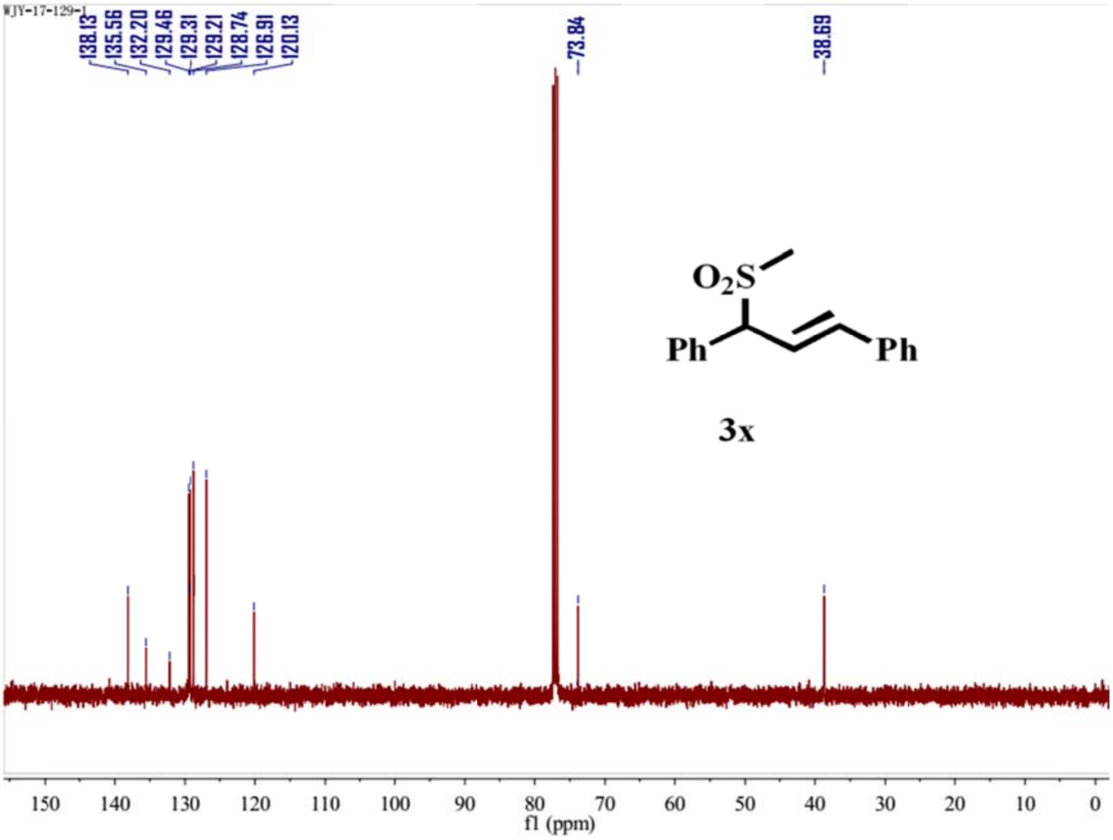

Supplementary Figure 50| ^{1}H NMR (400 MHz, $CDCl_3$) spectra for compound **3y.**

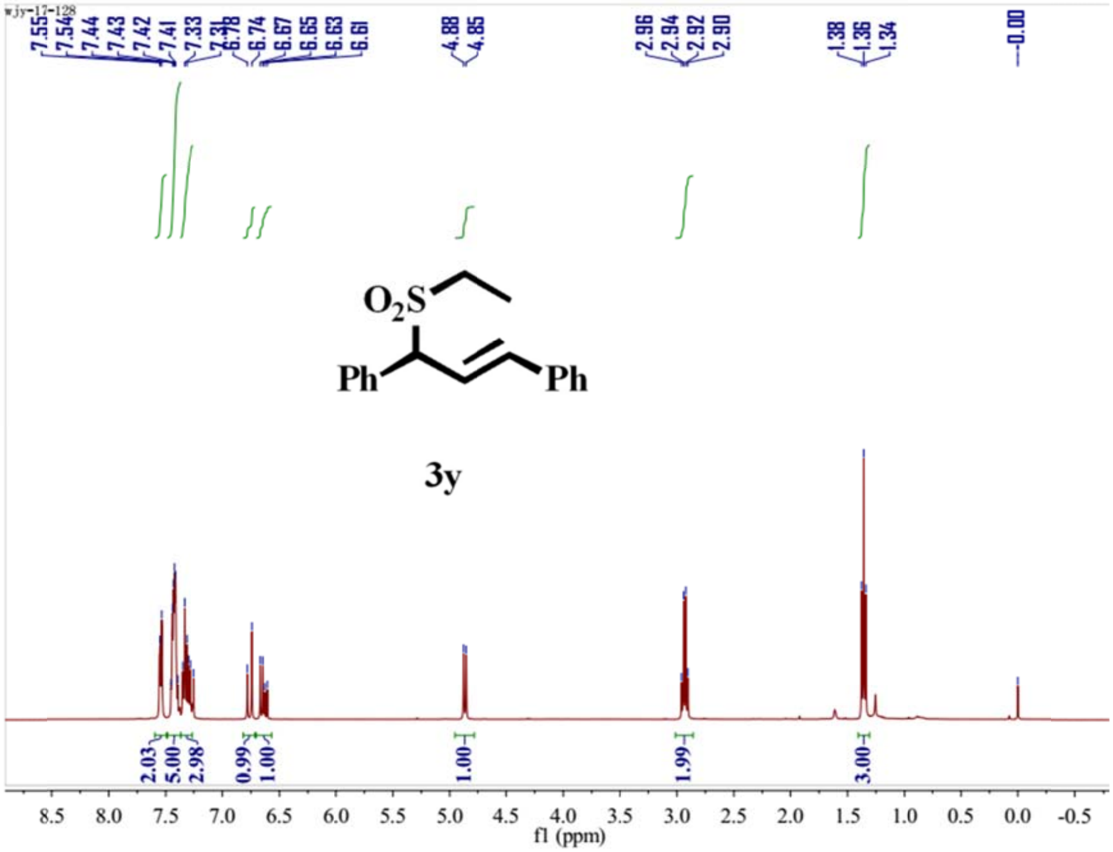

Supplementary Figure 51 | ^{13}C NMR (101 MHz, $CDCl_3$) spectra for compound **3y.**

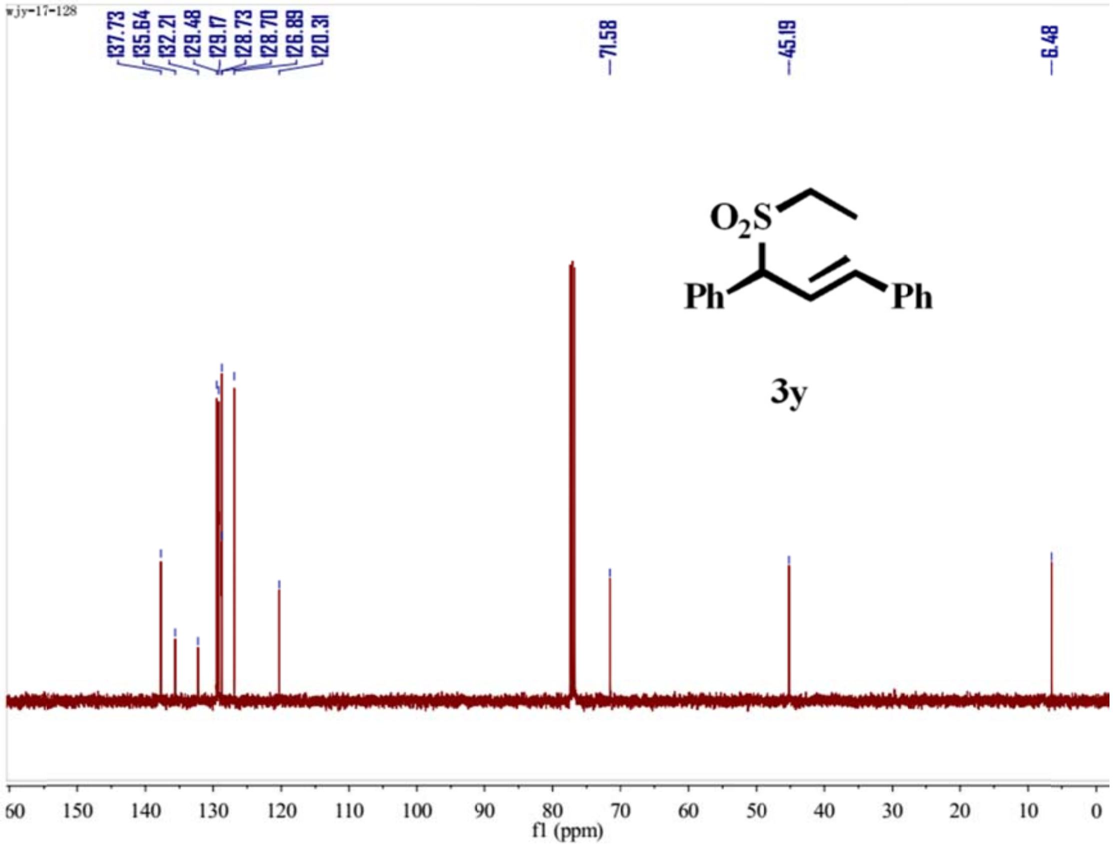

Supplementary Figure 52| ^{1}H NMR (400 MHz, $CDCl_3$) spectra for compound **3z.**

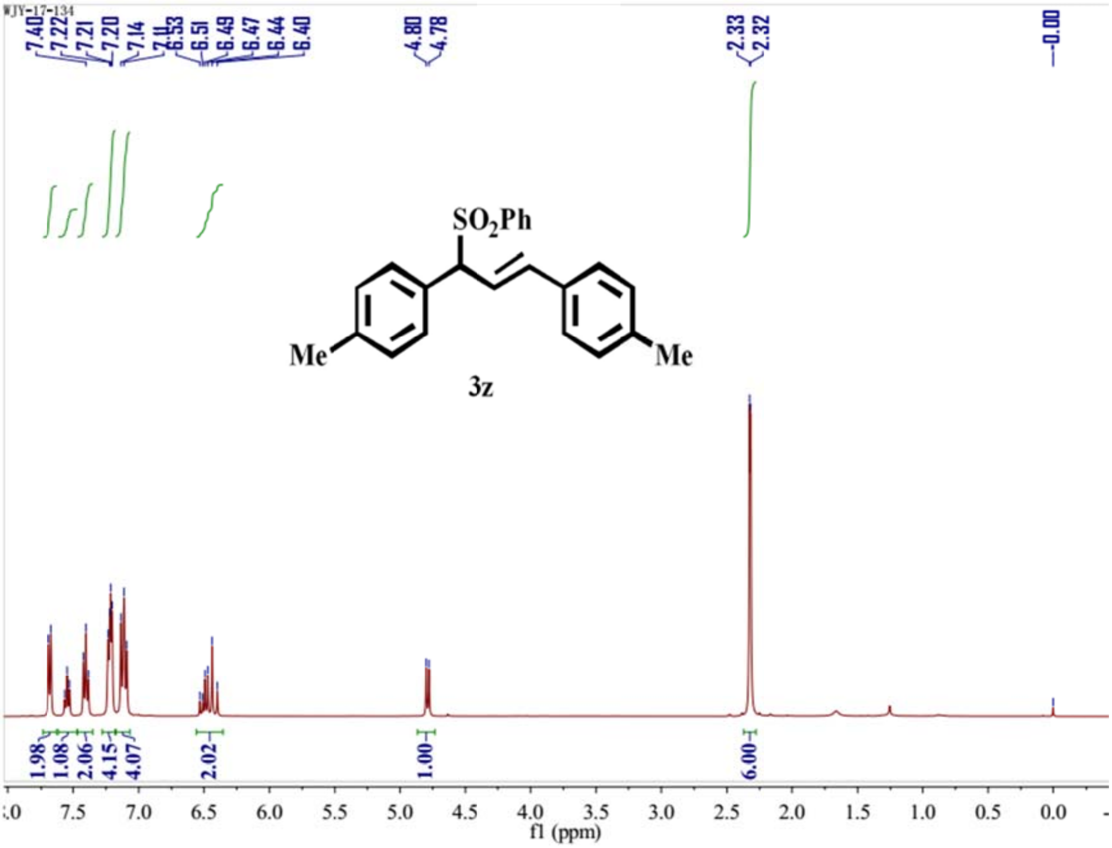

Supplementary Figure 53 | ^{13}C NMR (101 MHz, $CDCl_3$) spectra for compound **3z.**

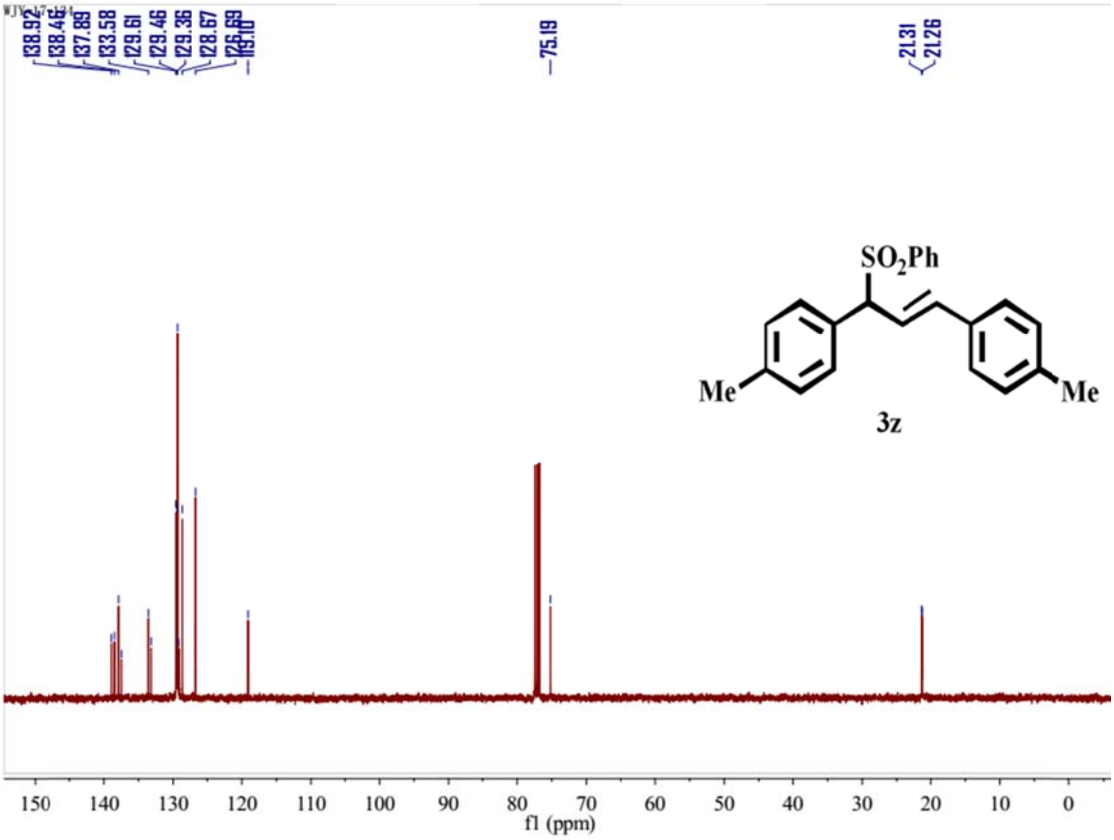

Supplementary Figure 54| ^{1}H NMR (400 MHz, $CDCl_3$) spectra for compound **3aa.**

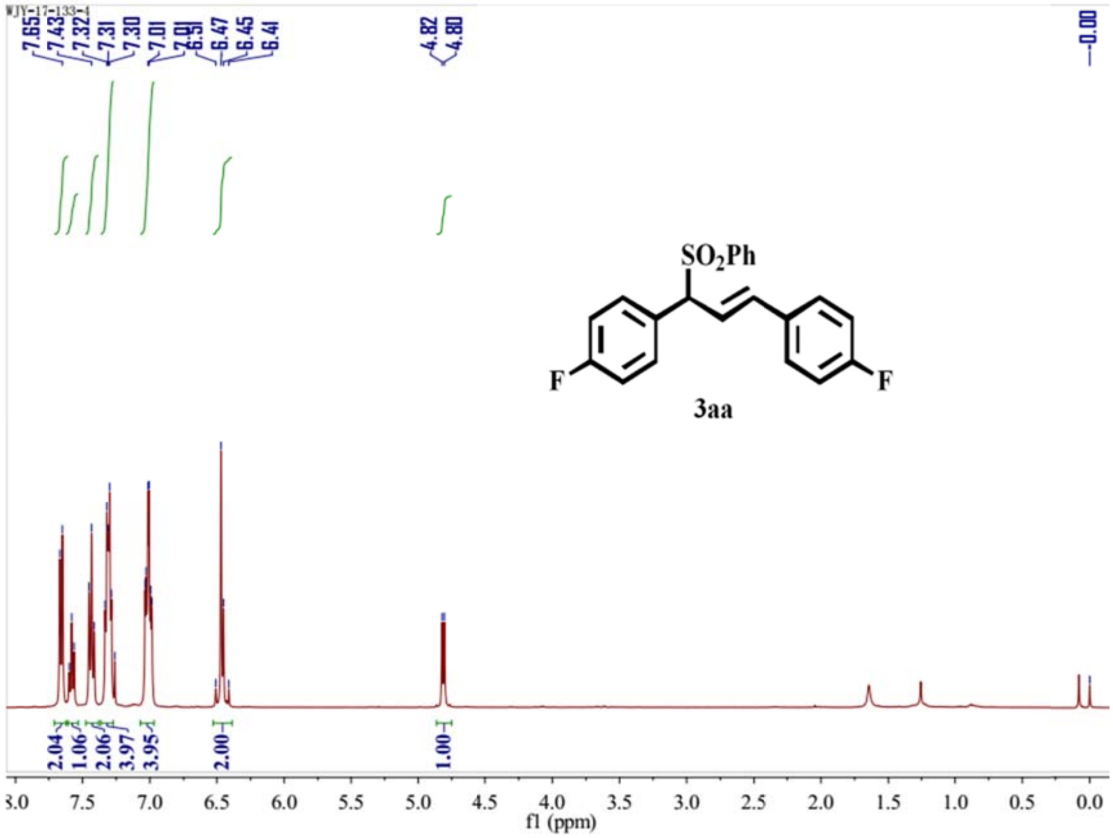

Supplementary Figure 55 | ^{13}C NMR (101 MHz, $CDCl_3$) spectra for compound **3aa.**

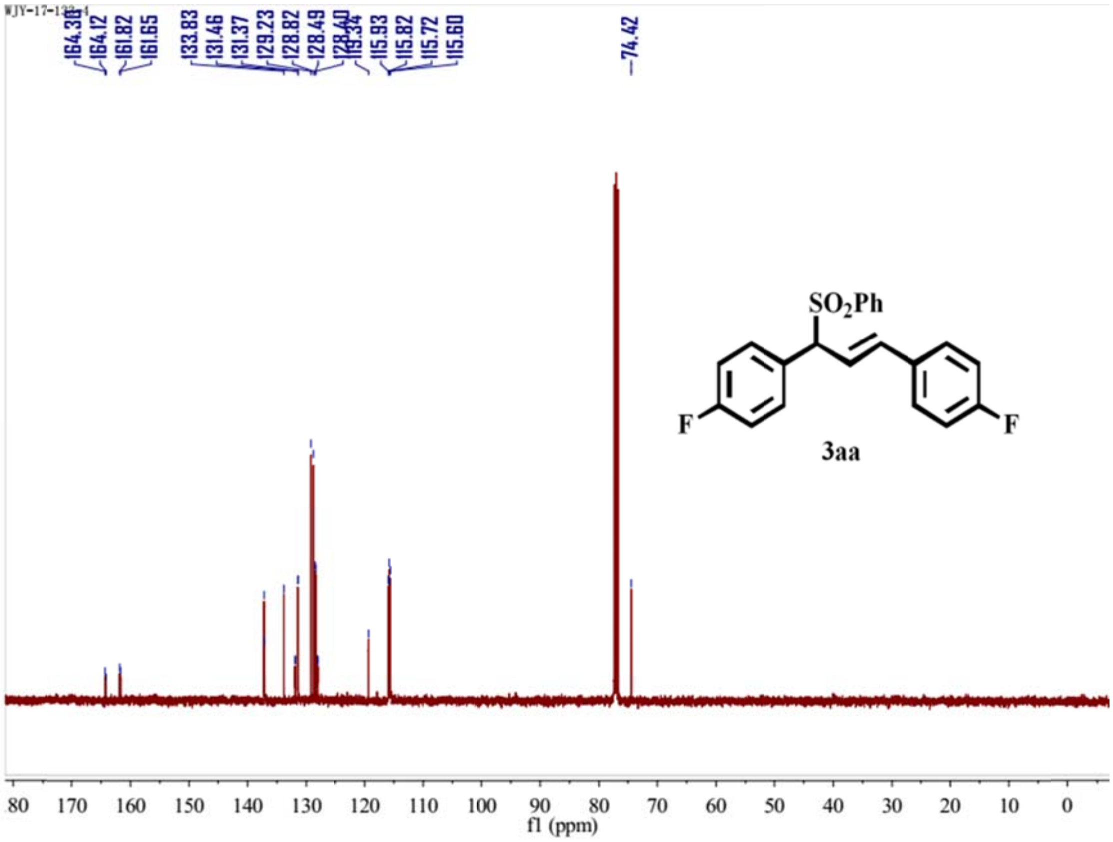

Supplementary Figure 56| ^{1}H NMR (400 MHz, $CDCl_3$) spectra for compound **3ab.**

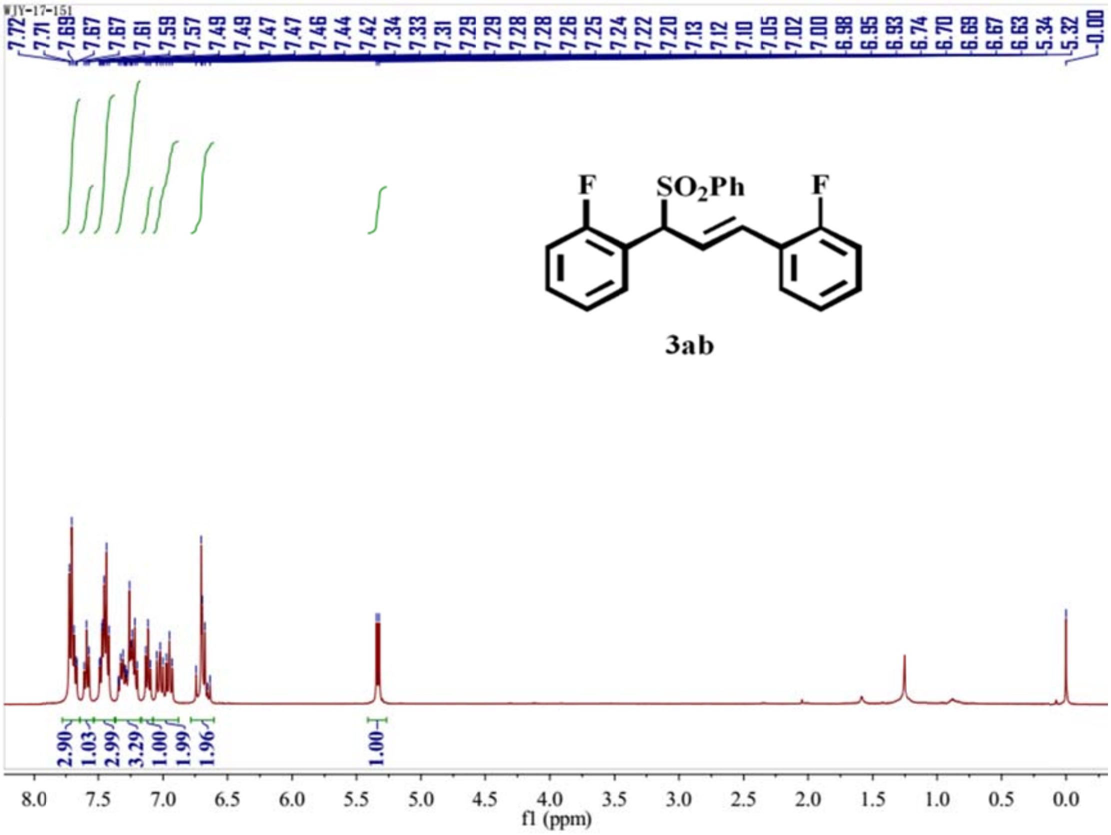

Supplementary Figure 57 | ^{13}C NMR (101 MHz, $CDCl_3$) spectra for compound **3ab.**

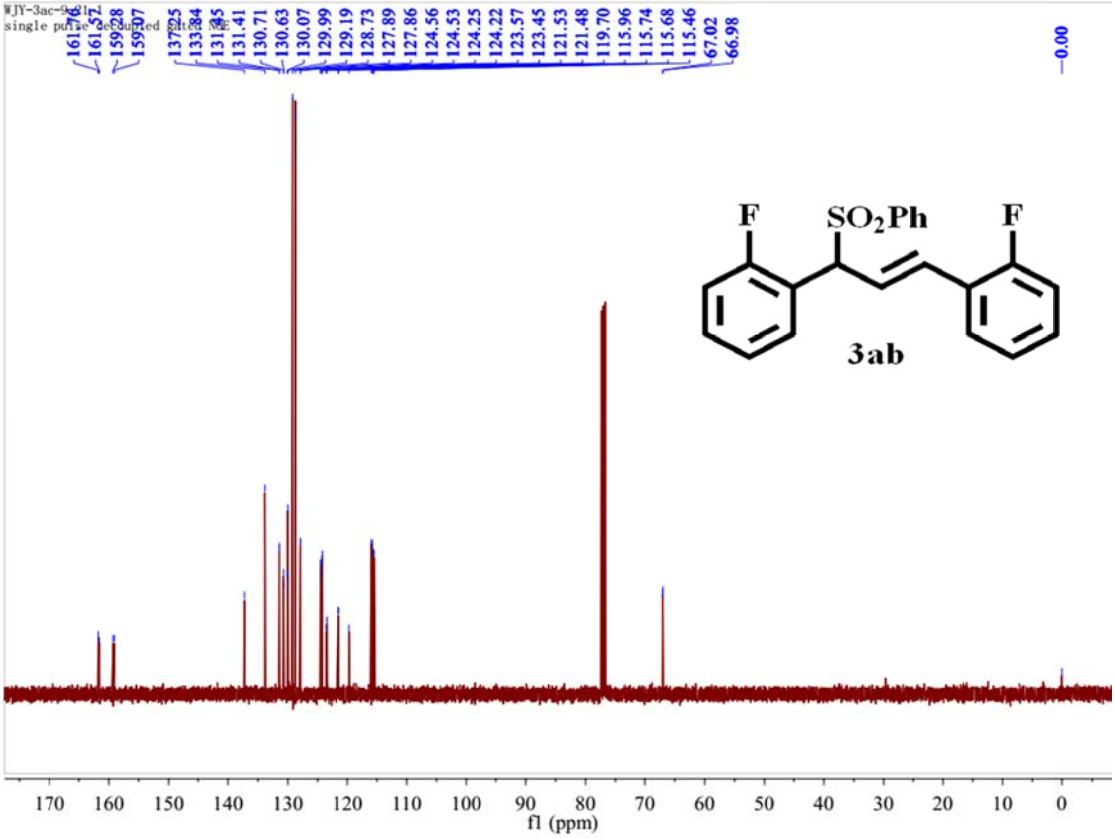

Supplementary Figure 58| ^{1}H NMR (400 MHz, $CDCl_3$) spectra for compound **3ac.**

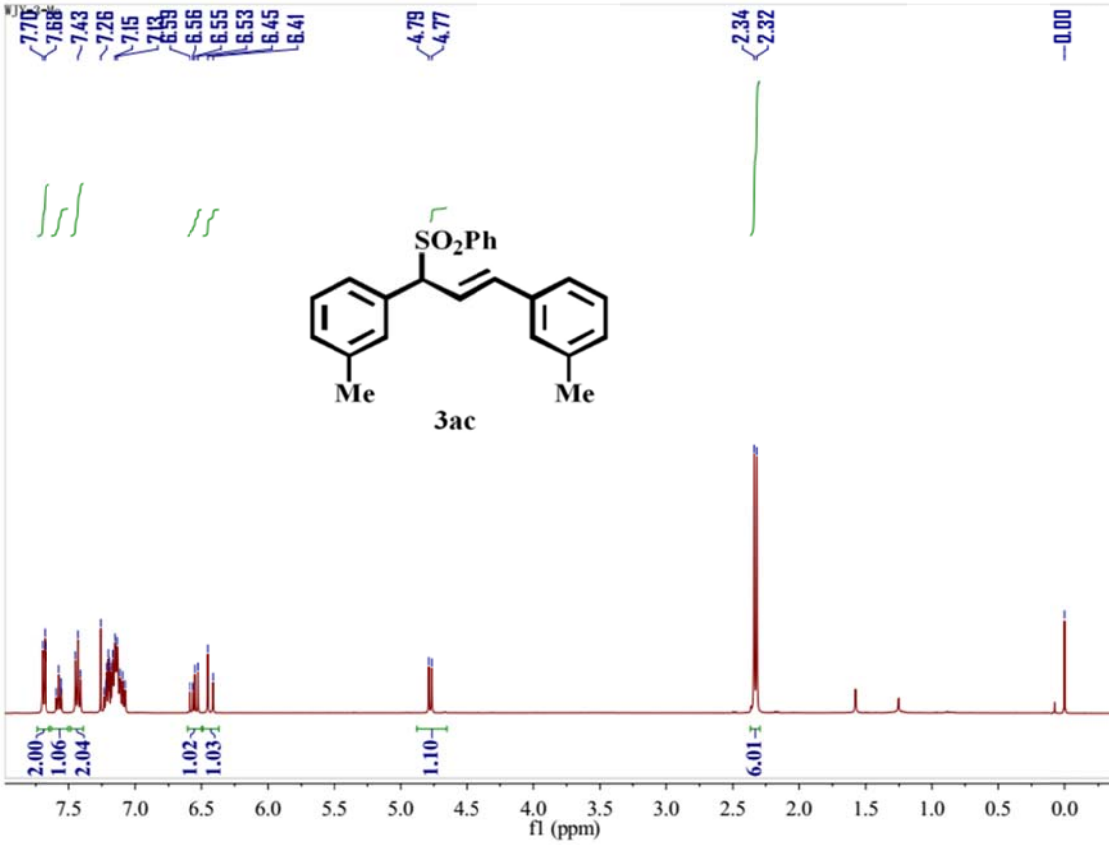

Supplementary Figure 59 | ^{13}C NMR (101 MHz, $CDCl_3$) spectra for compound **3ac.**

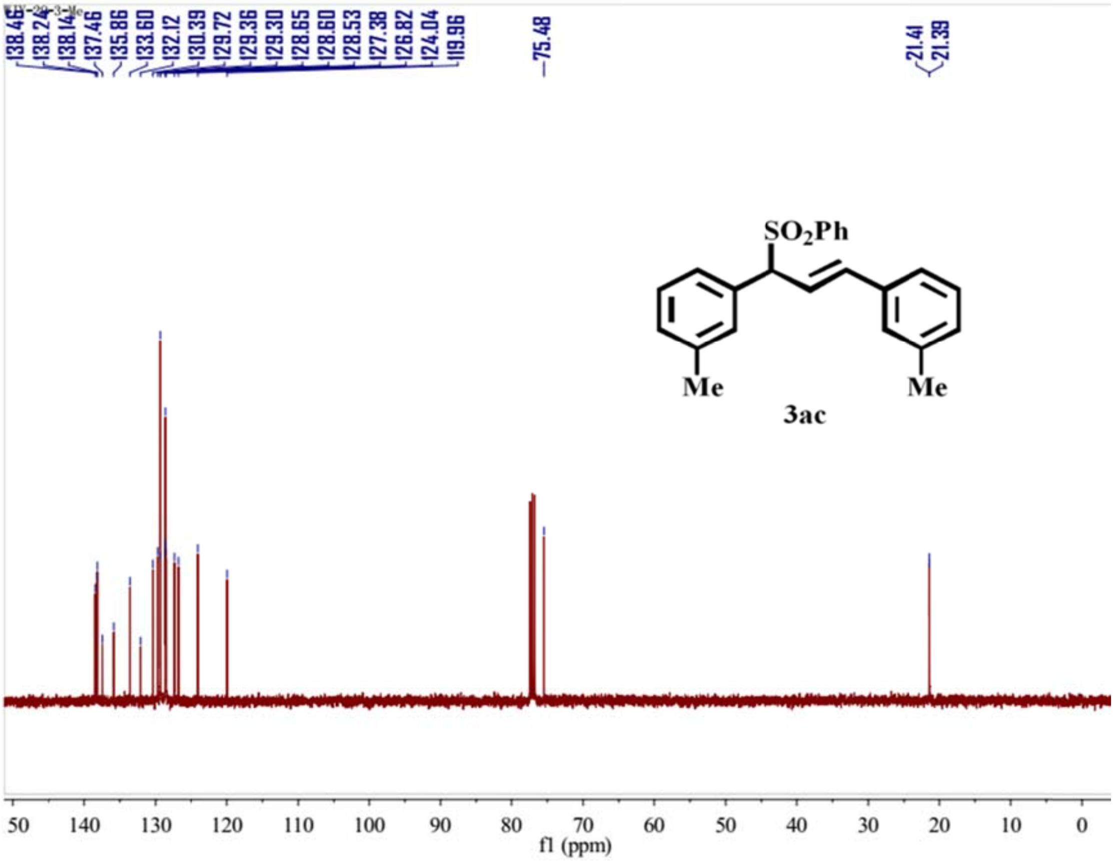

Supplementary Figure 60| ^{1}H NMR (400 MHz, $CDCl_3$) spectra for compound **3ad.**

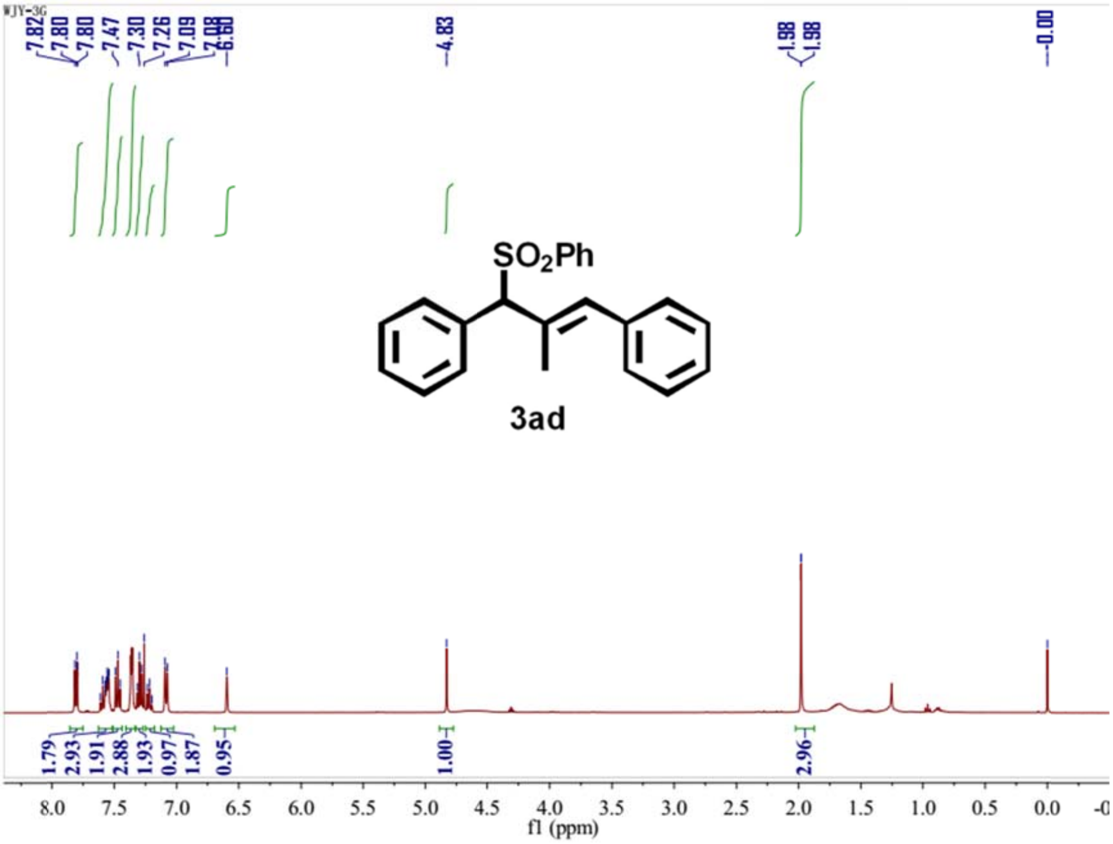

Supplementary Figure 61 | ^{13}C NMR (101 MHz, $CDCl_3$) spectra for compound **3ad.**

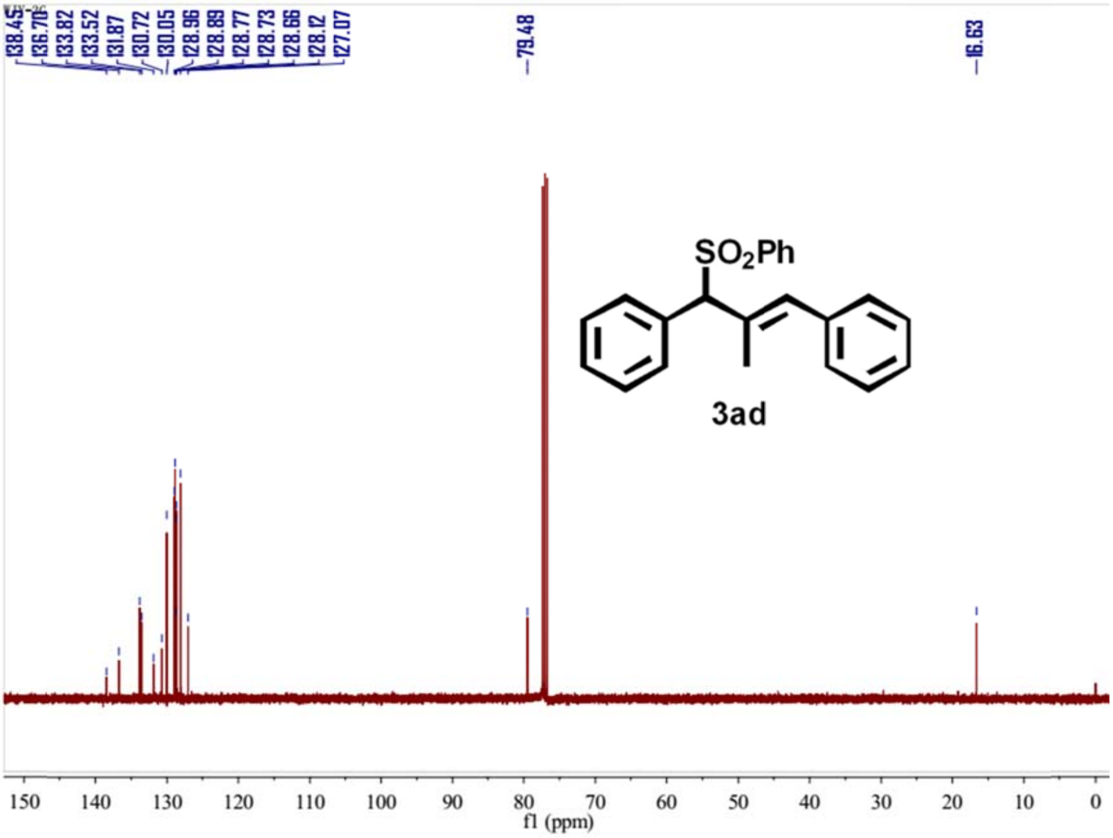

Supplementary Figure 62| ^{1}H NMR (400 MHz, $CDCl_3$) spectra for compound **3ae.**

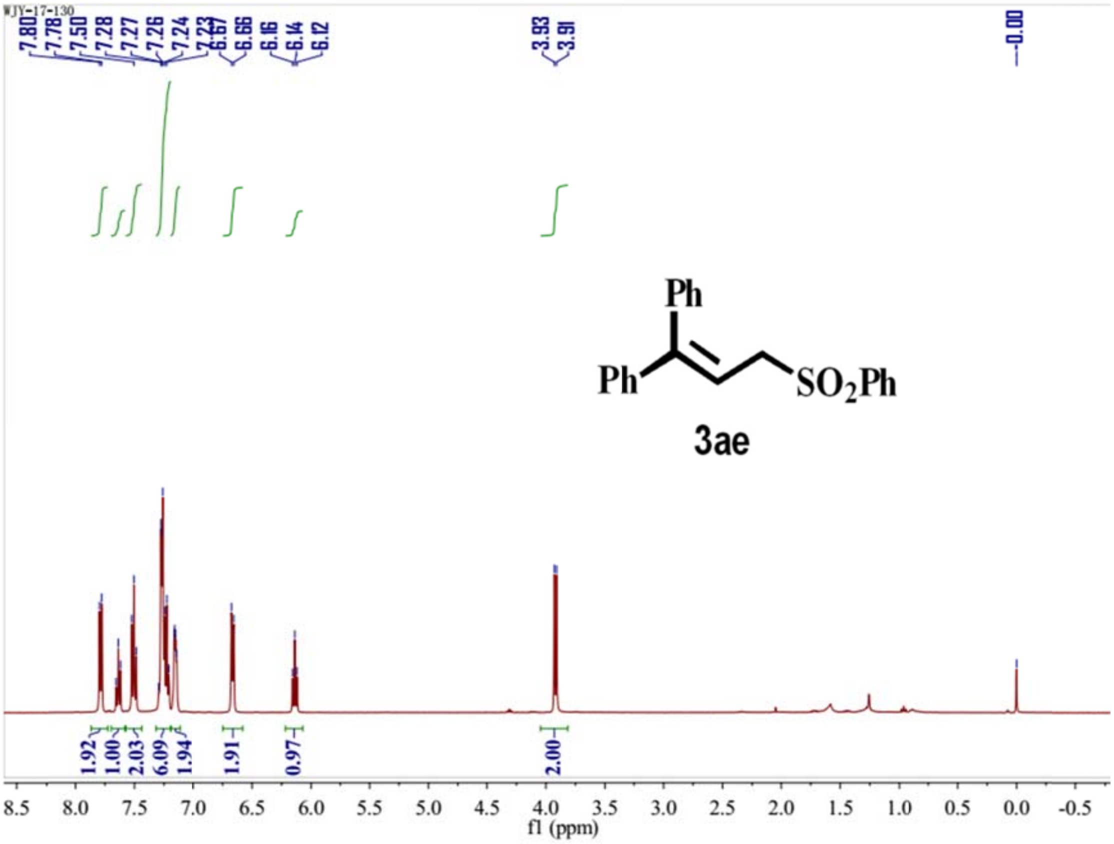

Supplementary Figure 63 | ^{13}C NMR (101 MHz, $CDCl_3$) spectra for compound **3ae.**

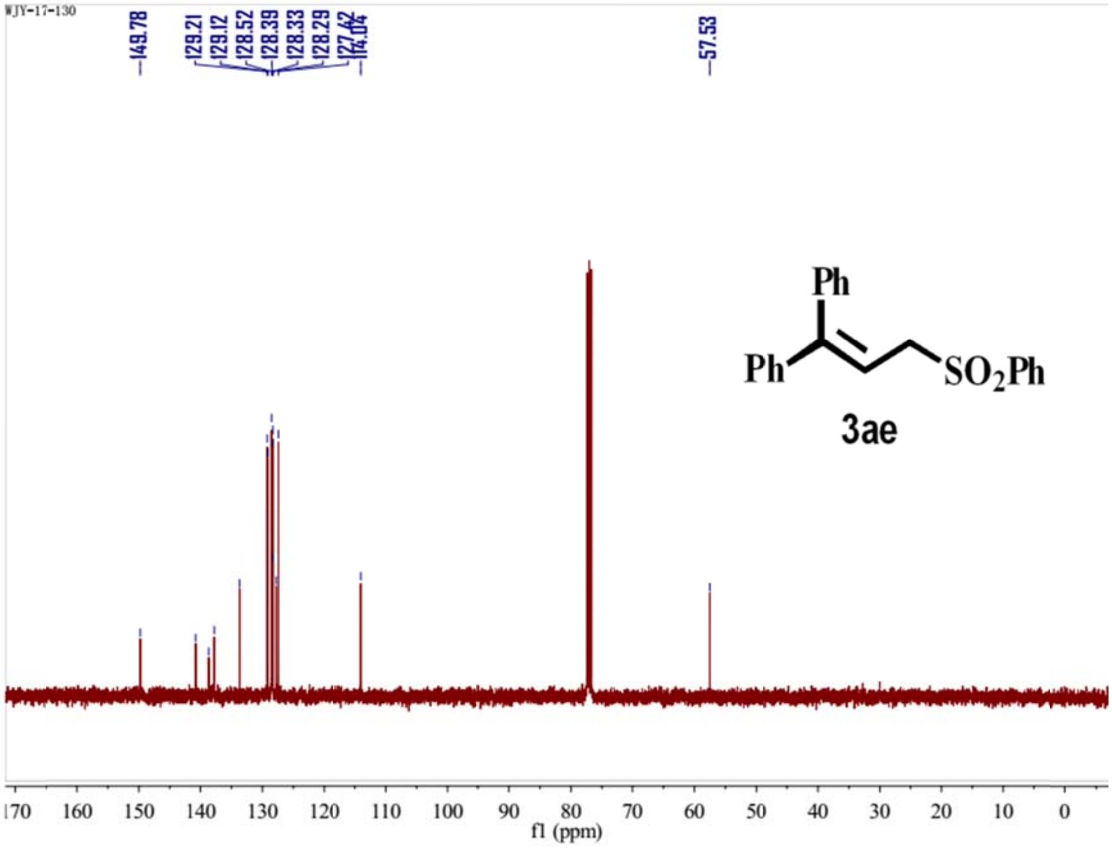

Supplementary Figure 64| ^{1}H NMR (400 MHz, $CDCl_3$) spectra for compound **3af.**

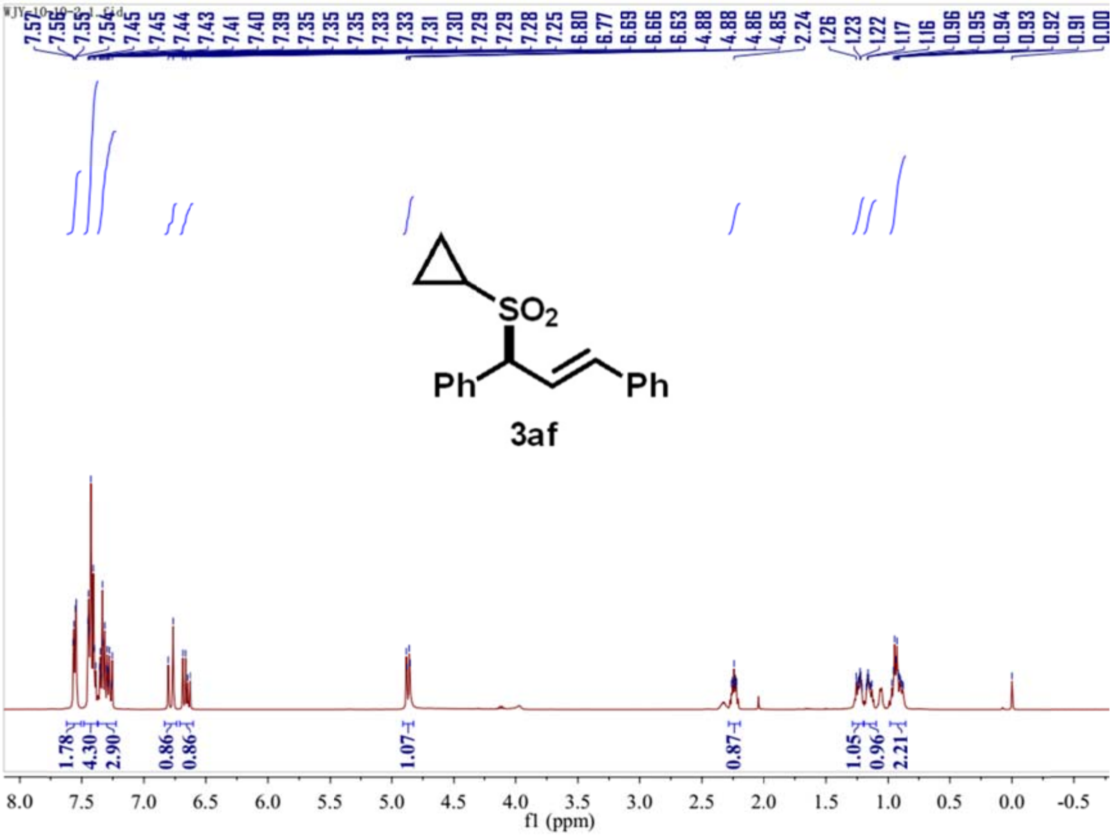

Supplementary Figure 65 | ^{13}C NMR (101 MHz, $CDCl_3$) spectra for compound **3af.**

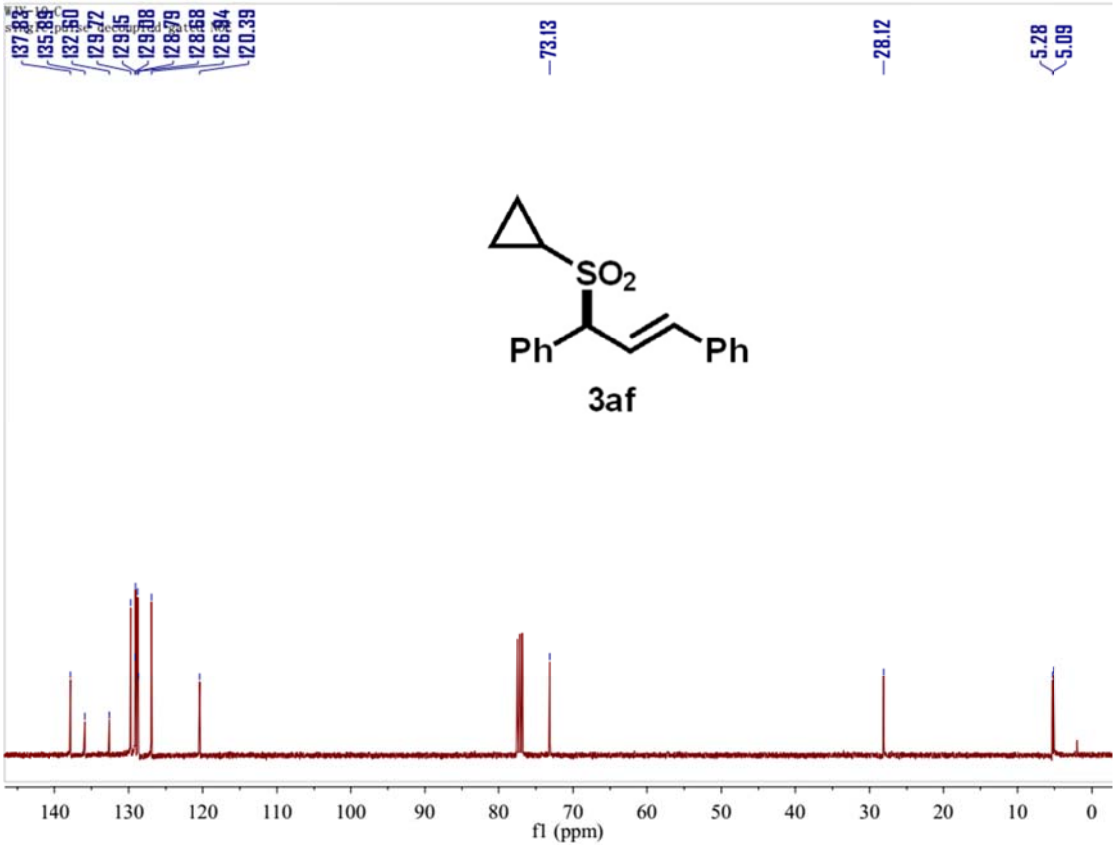

Supplementary Figure 66| ^{1}H NMR (400 MHz, $CDCl_3$) spectra for compound **3ag.**

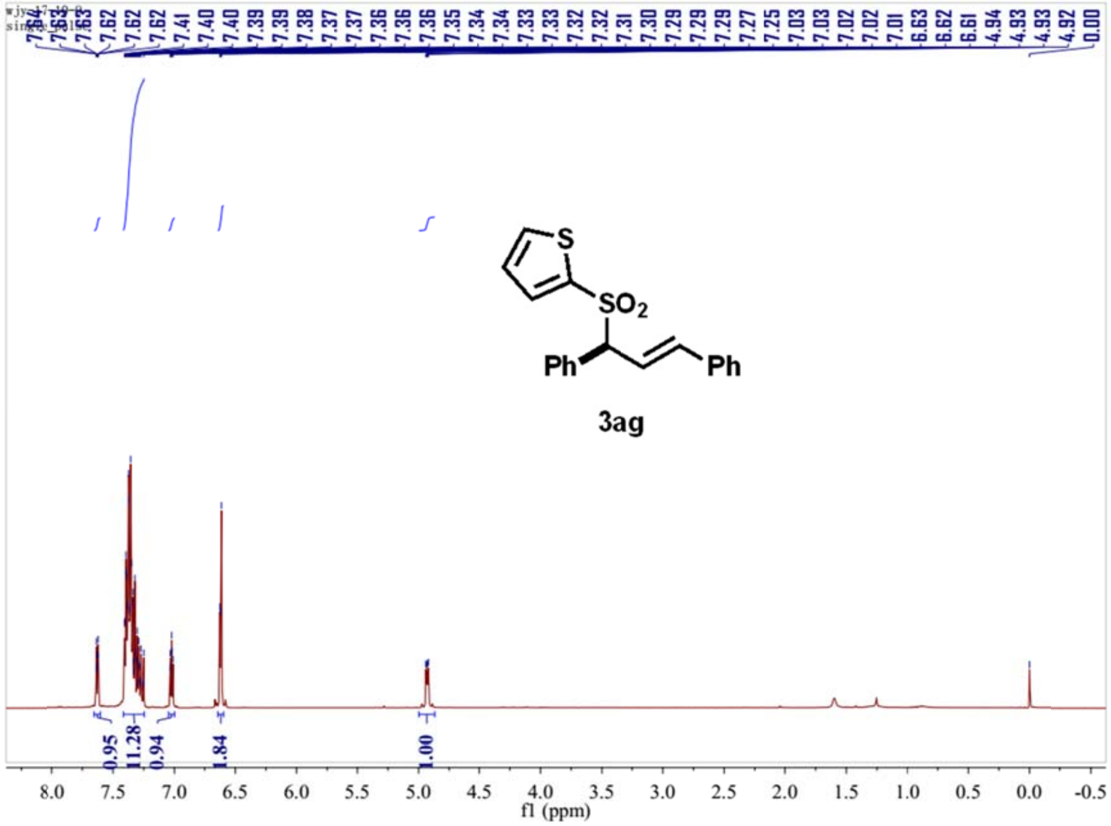

Supplementary Figure 67 | ^{13}C NMR (101 MHz, $CDCl_3$) spectra for compound **3ag.**

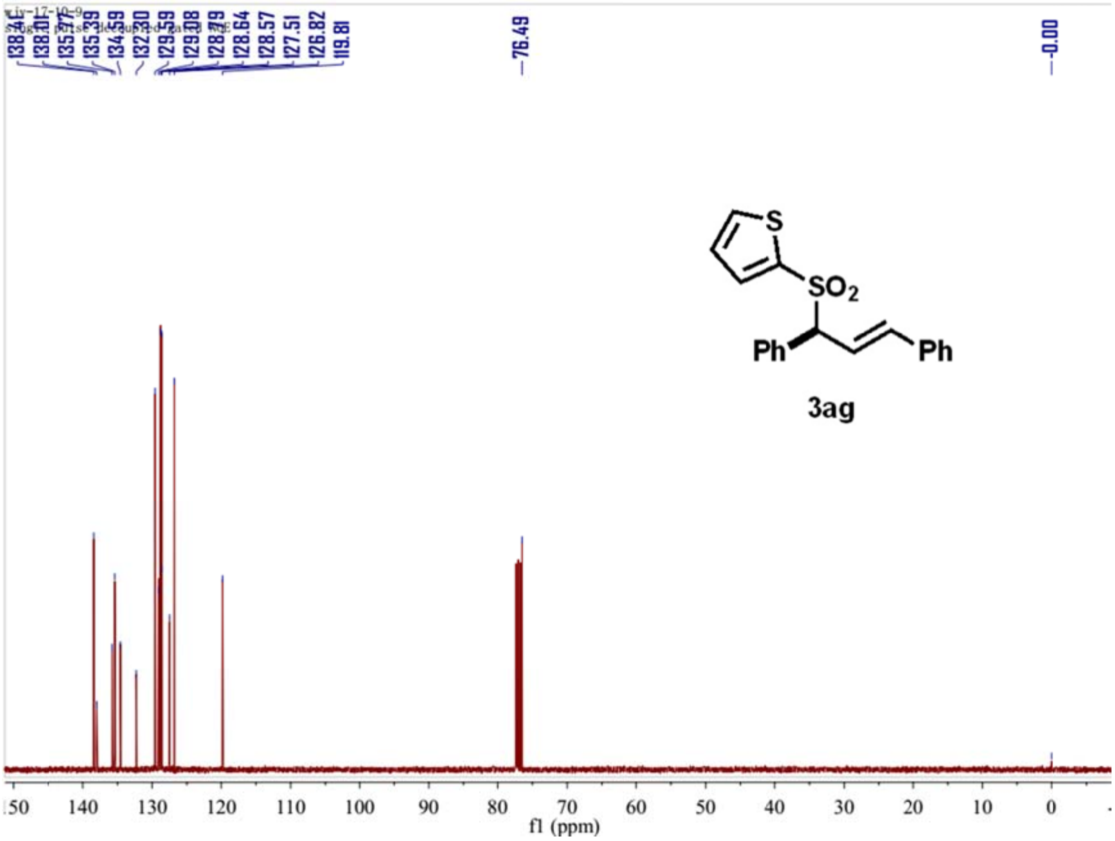

Supplementary Figure 68| ^{1}H NMR (400 MHz, $CDCl_3$) spectra for compound **3ah.**

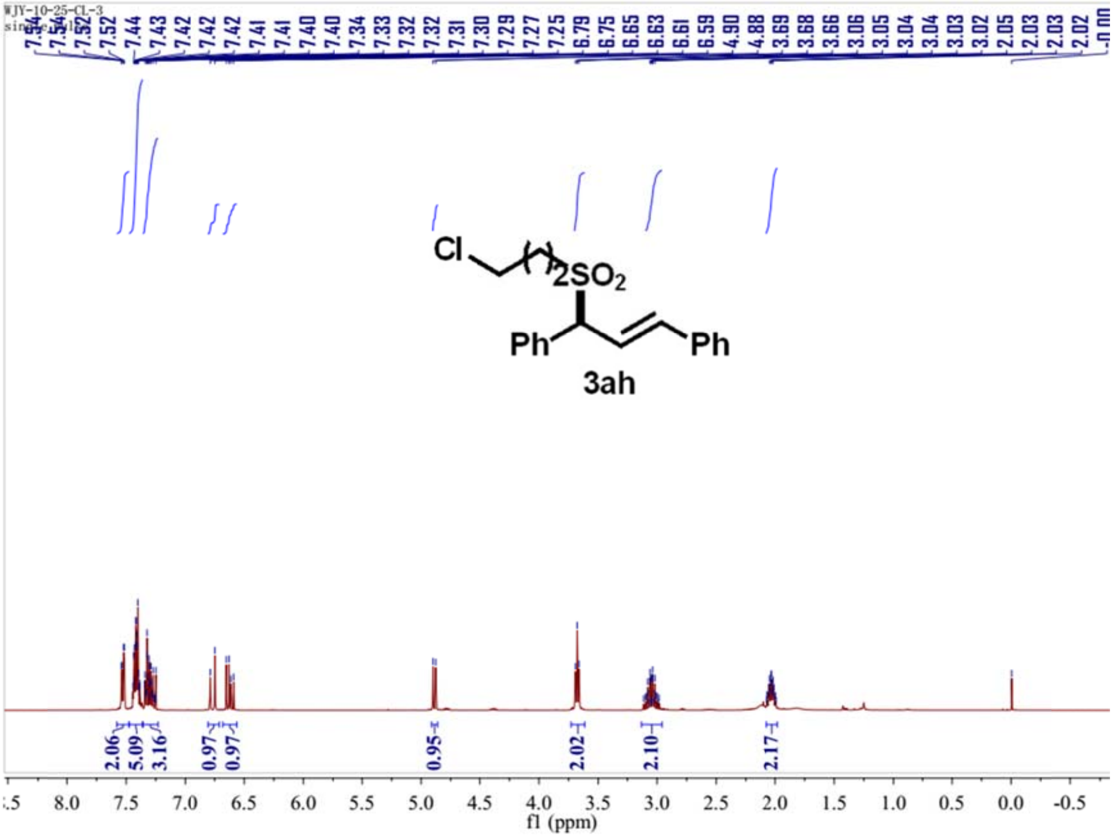

Supplementary Figure 69 | ^{13}C NMR (101 MHz, $CDCl_3$) spectra for compound **3ah.**

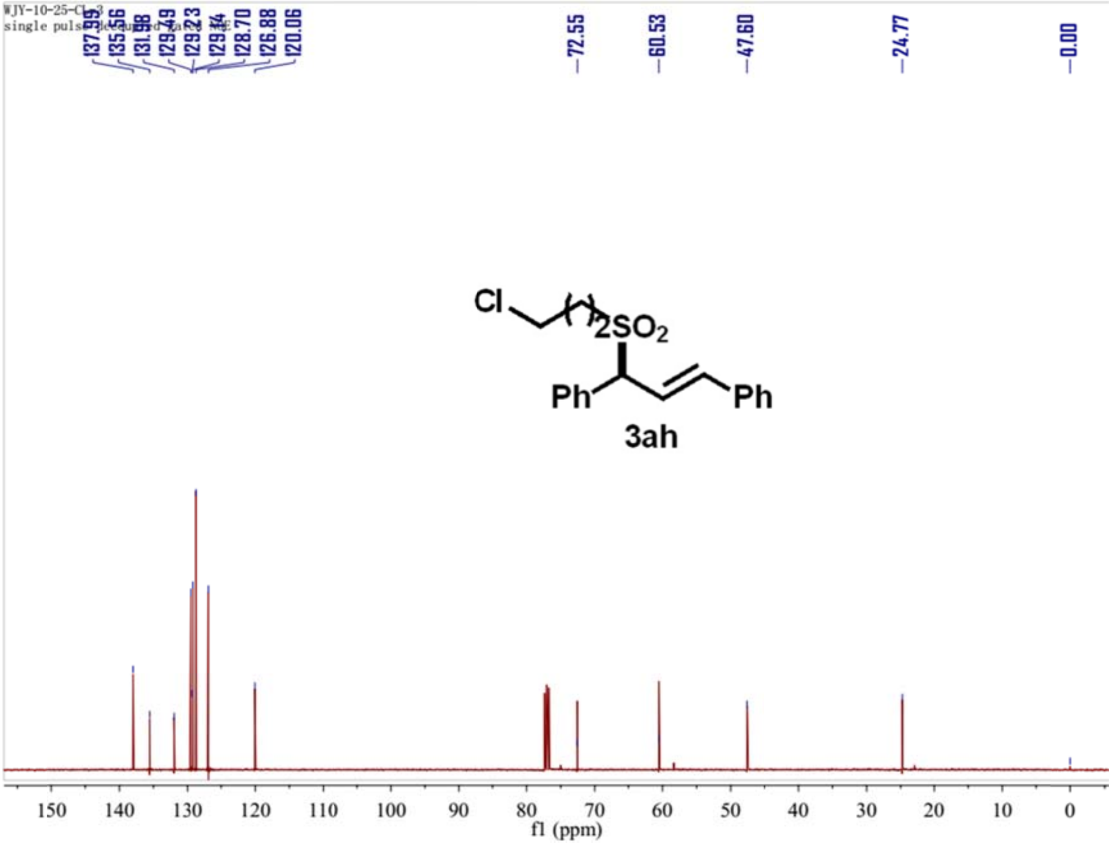

Supplementary Figure 70| ^{1}H NMR (400 MHz, $CDCl_3$) spectra for compound **3ai.**

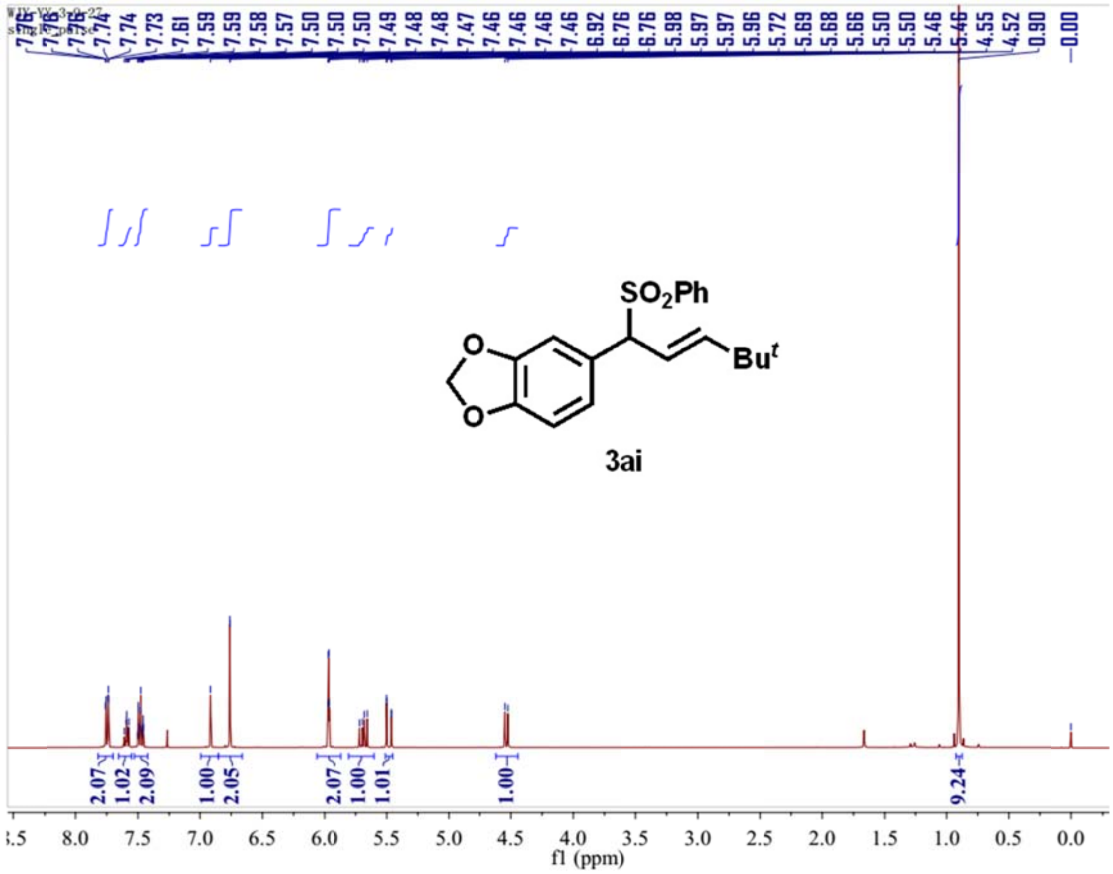

Supplementary Figure 71 | ^{13}C NMR (101 MHz, $CDCl_3$) spectra for compound **3ai.**

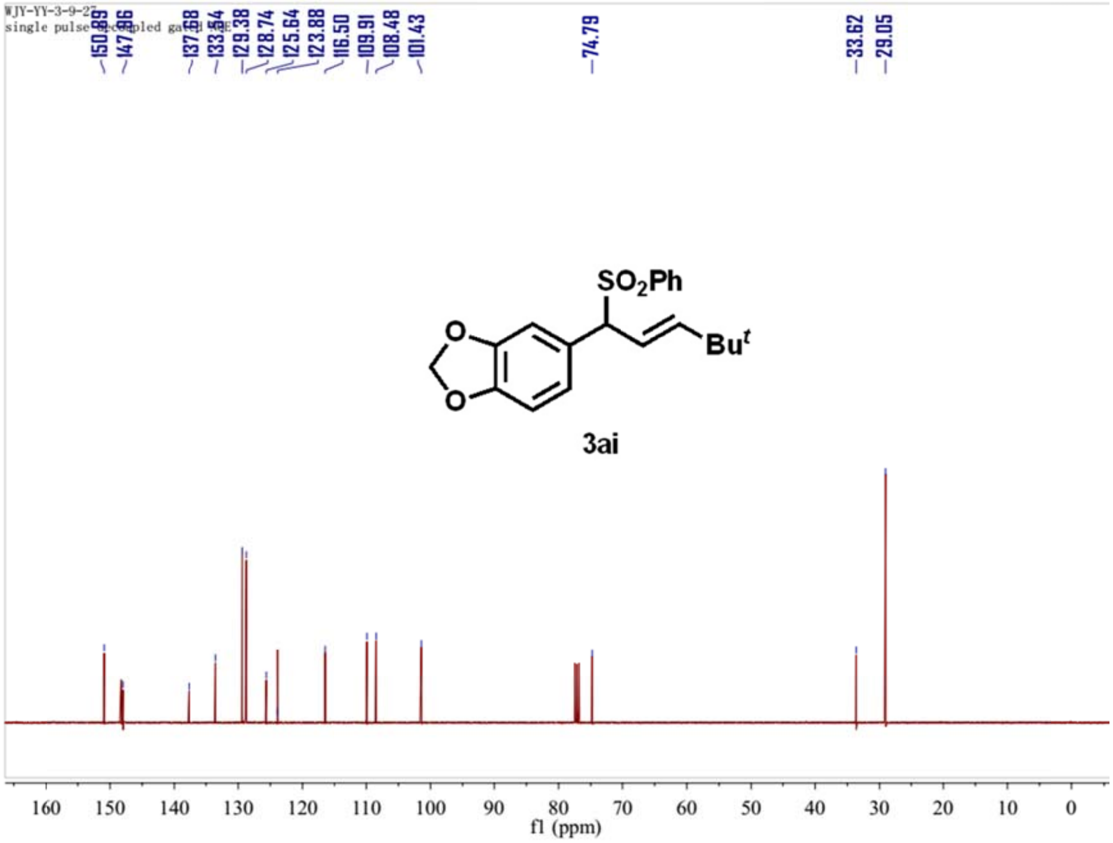

Supplementary Figure 72| ^{1}H NMR (400 MHz, DMSO-D6) spectra for compound **4.**

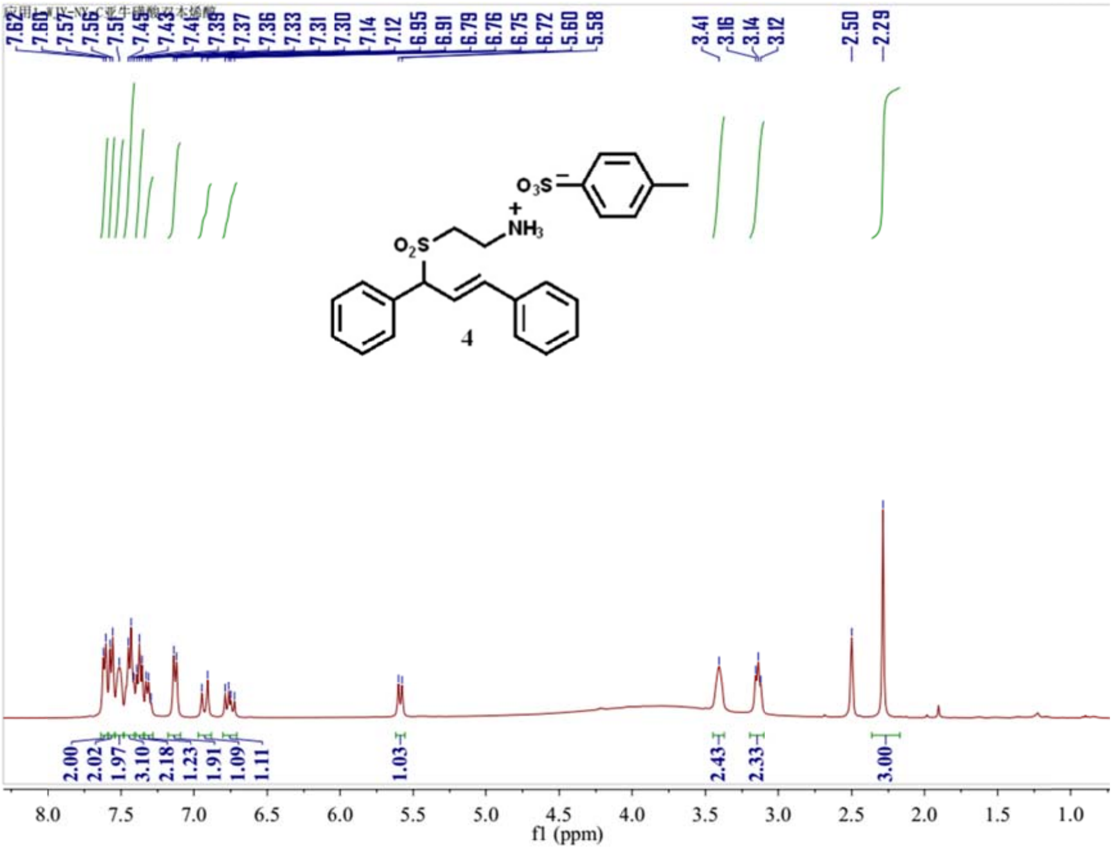

Supplementary Figure 73 | ^{13}C NMR (101 MHz, DMSO-D6) spectra for compound **4.**

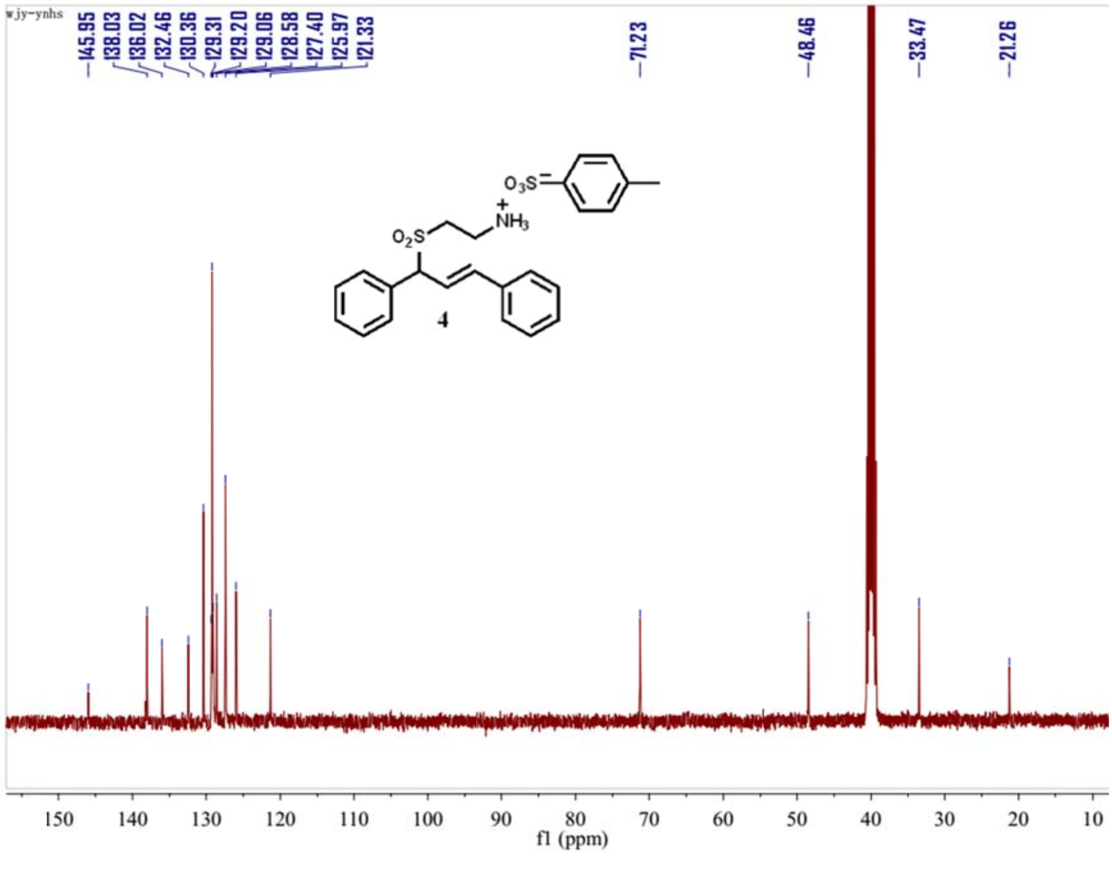

Supplementary Figure 74| ^{1}H NMR (400 MHz, DMSO-D6) spectra for compound **5.**

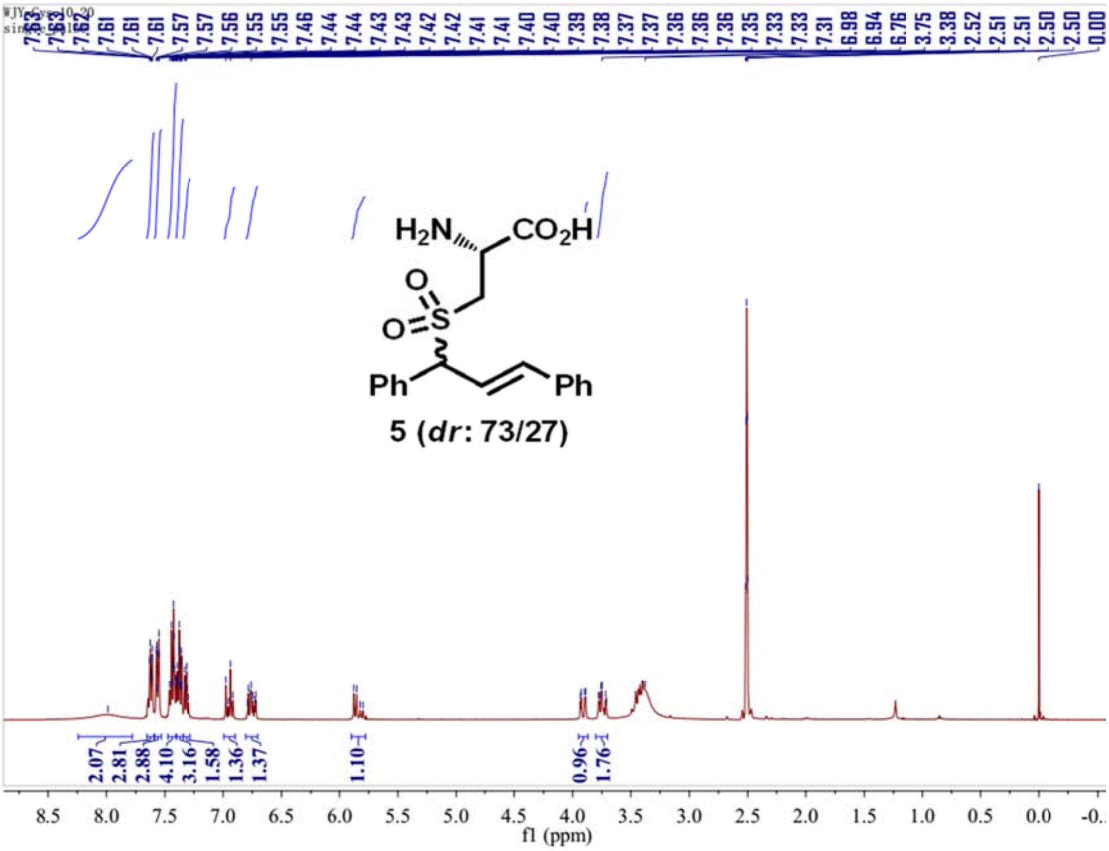

Supplementary Figure 75 | ^{13}C NMR (101 MHz, DMSO-D6) spectra for compound **5.**

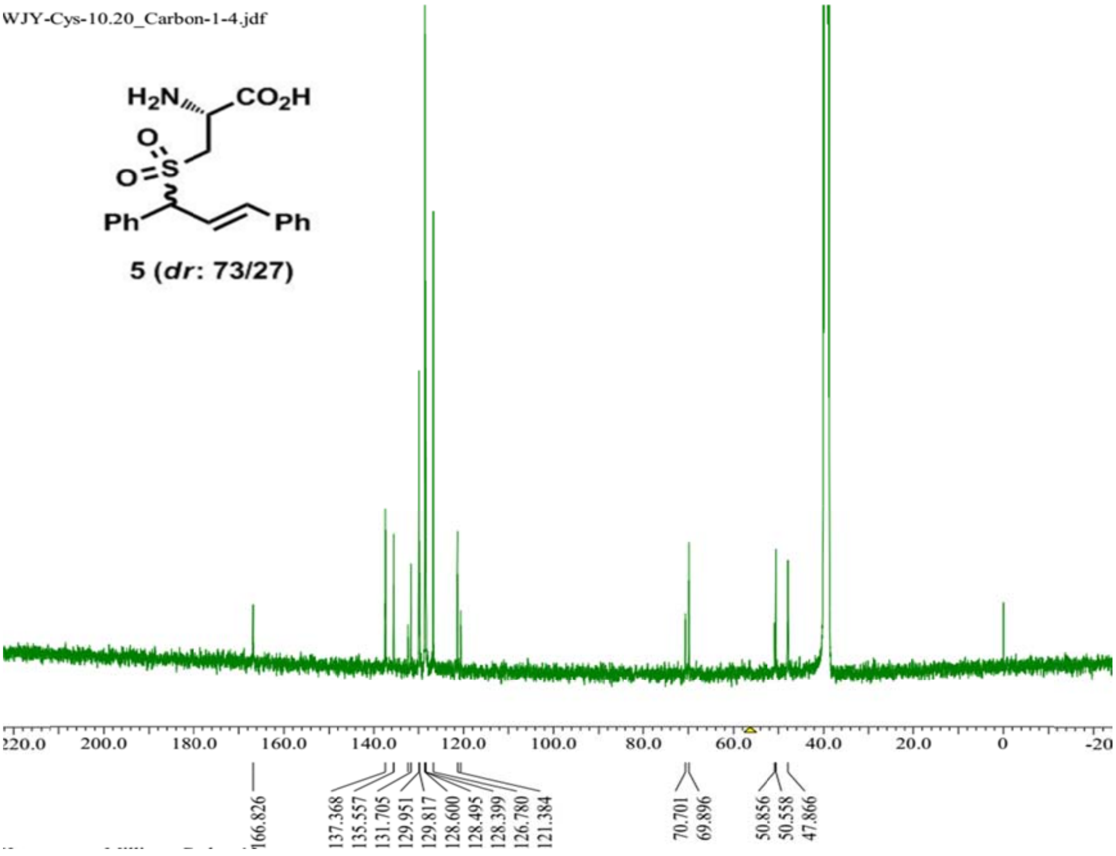

Supplementary Figure 76| ^{1}H NMR (400 MHz, $CDCl_3$) spectra for compound **6.**

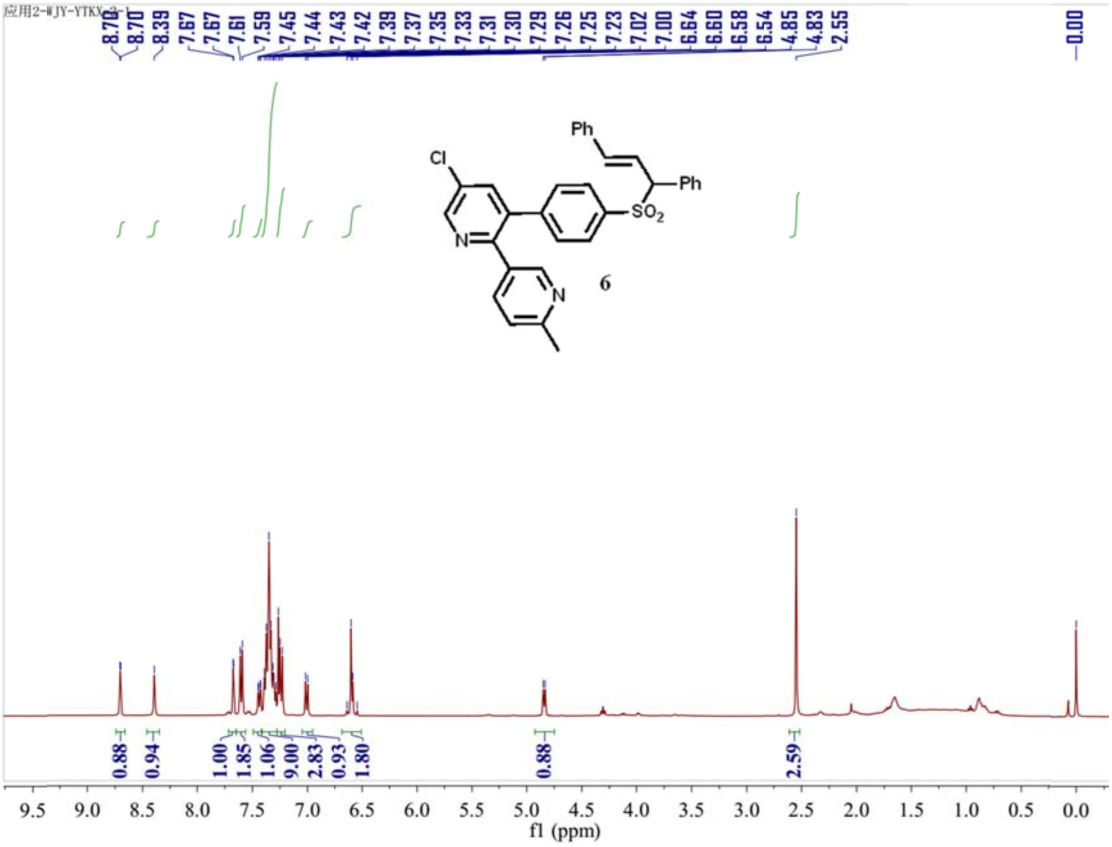

Supplementary Figure 77 | ^{13}C NMR (101 MHz, $CDCl_3$) spectra for compound **6.**

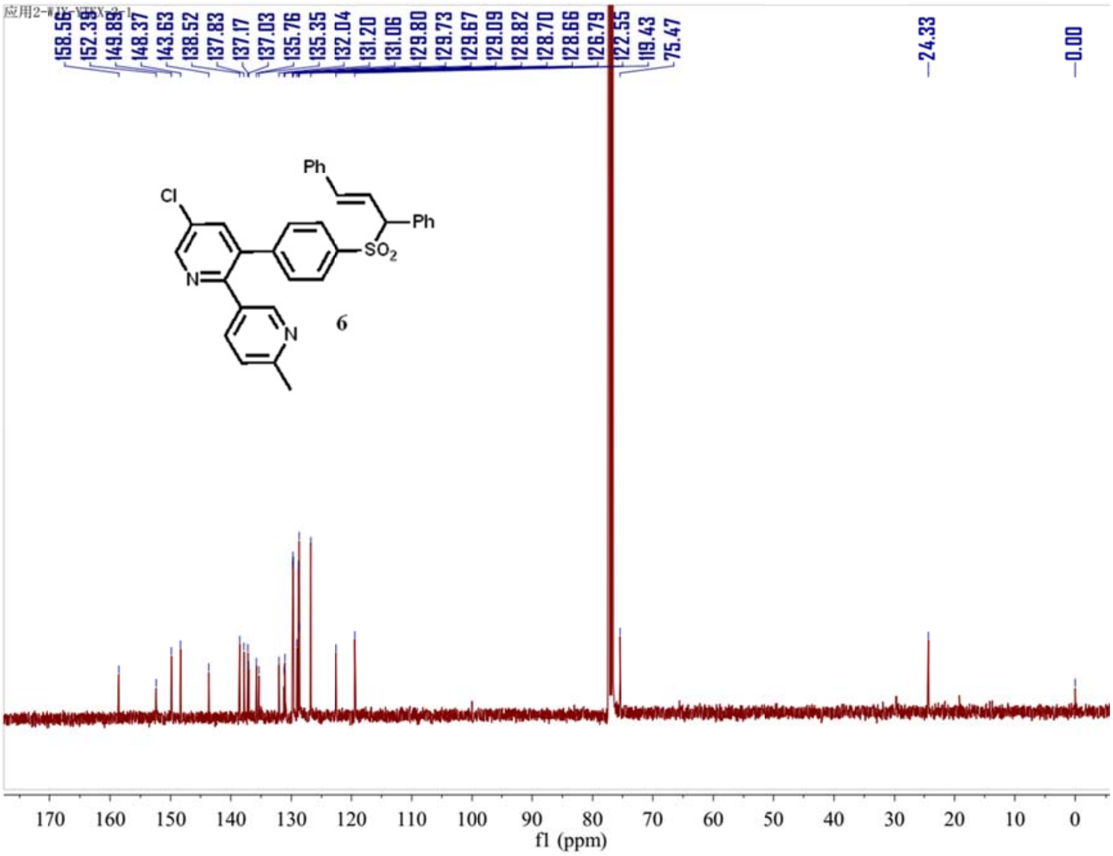

Supplementary Figure 78 | X-ray-3a.

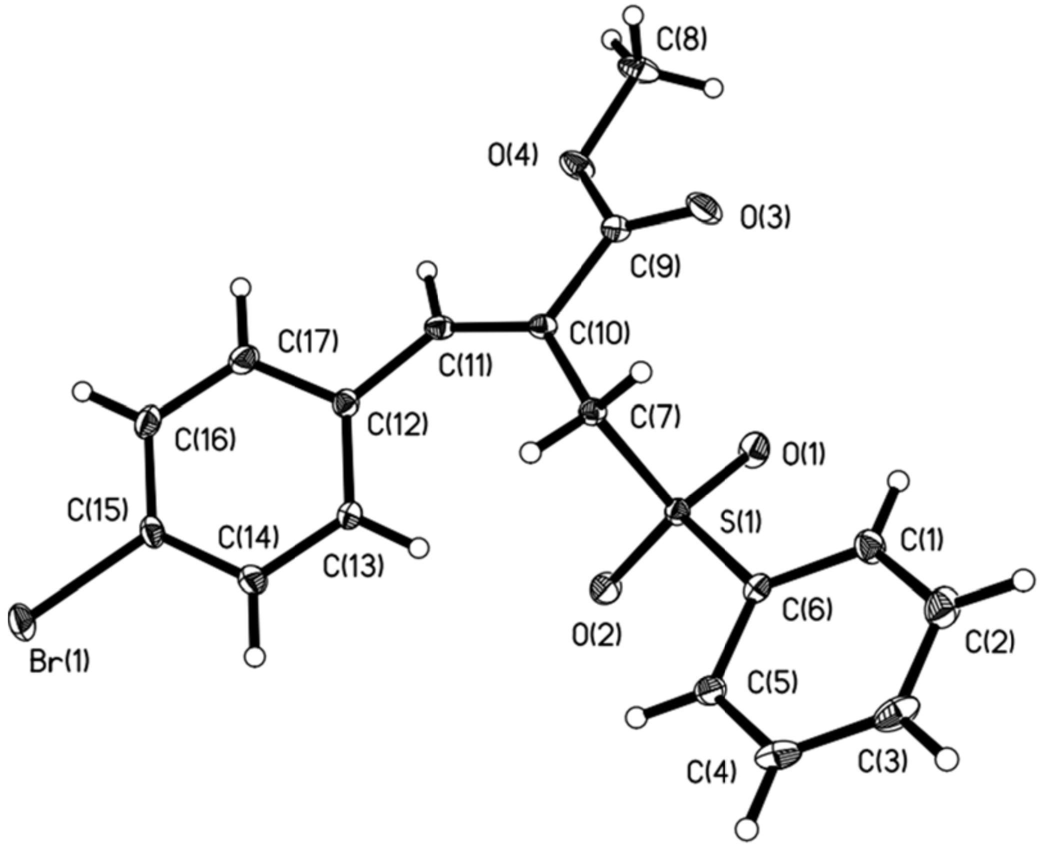

CCDC-1573430 (**3a**) contains the supplementary crystallographic data for this paper. These data can be obtained free of charge from The Cambridge Crystallographic Data Centre via www.ccdc.cam.ac. ac.uk/data_request/cif.

Supplementary References

1. Yuan, Z. *et al*. Highly Selective Pd-Catalyzed Intermolecular Fluorosulfonylation of Styrenes. *J. Am. Chem. Soc.* **137**, 2468-2471(2015).
2. Feng, J. Lu, X, Kong, A. & Han X. A highly regio- and stereo-selective [3+2] annulation of allylic compounds and 2-substituted 1,1-dicyanoalkenes through a catalytic carbon–phosphorus ylide reaction. Tetrahedron **63**, 6035-6041 (2007).
3. Gauthier. Jr, D. R. Yoshikawa, N. A General, One-Pot Method for the Synthesis of Sulfinic Acids from Methyl Sulfones. *Org. Lett.* **18**, 5994-5997(2016).
4. Gaussian 09, Revision A.02, M. J. Frisch, G. W. Trucks, H. B. Schlegel, G. E. Scuseria, M. A. Robb, J. R. Cheeseman, G. Scalmani, V. Barone, B. Mennucci, G. A. Petersson, H. Nakatsuji, M. Caricato, X. Li, H. P. Hratchian, A. F. Izmaylov, J. Bloino, G. Zheng, J. L. Sonnenberg, M. Hada, M. Ehara, K. Toyota, R. Fukuda, J. Hasegawa, M. Ishida, T. Nakajima, Y. Honda, O. Kitao, H. Nakai, T. Vreven, J. A. Montgomery, Jr., J. E. Peralta, F. Ogliaro, M. Bearpark, J. J. Heyd, E. Brothers, K. N. Kudin, V. N. Staroverov, R. Kobayashi, J. Normand, K. Raghavachari, A. Rendell, J. C. Burant, S. S. Iyengar, J. Tomasi, M. Cossi, N. Rega, J. M. Millam, M. Klene, J. E. Knox, J. B. Cross, V. Bakken, C. Adamo, J. Jaramillo, R. Gomperts, R. E. Stratmann, O. Yazyev, A. J. Austin,

R. Cammi, C. Pomelli, J. W. Ochterski, R. L. Martin, K. Morokuma, V. G. Zakrzewski, G. A. Voth, P. Salvador, J. J. Dannenberg, S.
